# Benzimidazol-2-ylidene ruthenium complexes for C–N bond formation through alcohol dehydrogenation

**DOI:** 10.55730/1300-0527.3606

**Published:** 2023-09-30

**Authors:** Zahid NAWAZ, Nevin GÜRBÜZ, Muhammad Naveed ZAFAR, Namık ÖZDEMIR, Bekir ÇETİNKAYA, İsmail ÖZDEMİR

**Affiliations:** 1 Department of Chemistry, Quaid-i-Azam University, Islamabad, Pakistan; 2 Catalysis Research and Application Center, İnönü University, Malatya, Turkiye; 3 Department of Chemistry, Faculty of Science and Arts, İnönü University, Malatya, Turkiye; 4 Drug Application and Research Center, İnönü University, Malatya, Turkiye; 5 Department of Mathematics and Science Education, Faculty of Education, Ondokuz Mayıs University, Samsun, Turkiye; 6 Department of Chemistry, Faculty of Science, Ege University, İzmir, Turkiye

**Keywords:** Benzimidazol*-*2-ylidenes, ruthenium complexes, amine alkylation, C-N bond formation, mild conditions

## Abstract

A low temperature hydrogen borrowing approach to generate secondary amines using benzimidazole-based *N*-heterocyclic carbene (BNHC) ruthenium complexes is reported. A series of the piano-stool complexes of the type [(η^6^-*p*-cymene)(BNHC)RuCl_2_] (**1a**–**g**) were synthesized via one-pot reaction of the NHC salt precursor, Ag_2_O, and [RuCl_2_(p-cymene)]_2_ and characterized using conventional spectroscopic techniques. The geometry of two precursors, [(η^6^-*p*-cymene)(^Me4Bn^Me_2_BNHC^CH2OxMe^)RuCl_2_] (**1f**) and [(η^6^-*p*-cymene)(^Me5Bn^Me_2_BNHC^CH2OxMe^)RuCl_2_] (**1g**), was studied by single crystal X-ray diffraction. These catalysts were found to dehydrogenate alcohols efficiently at temperatures as low as 50 °C to allow Schiff-base condensation and subsequent imine hydrogenation to afford secondary amines. Notably, this ruthenium-based procedure enables the *N*-alkylation of aromatic and heteroaromatic primary amines with a wide range of primary alcohols in excellent yields of up to 98%. The present methodology is green and water is liberated as the sole byproduct.

## 1. Introduction

Amines, organic derivatives of ammonia, are extensively found in bioactive molecules and medicines [1]. Amines are the key precursor in the manufacture of a number of relevant therapeutics medicines [2–4]. Conventionally, the most common methods for producing alkylated amines involve alkyl halides [5] or stoichiometric reducing agents, which are used for reduction of imines formed between carbonyls and amines [6,7]. The toxicity of the alkylating and reducing reagents and the generation of huge volumes of undesired byproducts are all significant disadvantages of these reactions. To address these difficulties, catalytic techniques have been devised including Buchwald–Hartwig amination [8], hydroamination [9,10], and hydroaminomethylation [11] as well as hydrogen borrowing or hydrogen autotransfer (HB/HA) methodologies [12].

In the HB/HA procedure, first dehydrogenation of the alcohol produces the equivalent aldehyde, which then undergoes reductive amination to produce the required amine. Because the alcohol functions as the hydrogen donor, an additional hydrogen source is not required in this approach. Furthermore, because a variety of alcohol derivatives are easily available from renewable feedstocks, this technology is particularly well suited for the valorization of biomass or biomass-derived building blocks. The HB/HA technique is the most attractive methodology for their synthesis [13–15]. These reactions are notable for being not only ecologically friendly, but also atom efficient, with only water as a byproduct. Grigg [16] and Watanabe [17] independently described the first examples of amine alkylation with alcohols via hydrogen borrowing while employing the homogeneous ruthenium catalysts [(PPh_3_)_4_RhH] and [(PPh_3_)_3_RuCl_2_]. Since that time, several noble metal-based Ru [18–21], Pd [22–24], Ir [25–27], and Pt [28] complexes and nonnoble metal-based Mn [29] Co [30] Ni [31], and Fe [32] complexes have been used. Heterogeneous catalysts [33], biocatalysts [34,35], and chiral catalysts [36,37] have also been used. Importantly, many of the catalysts that have previously been described for this reaction require relatively high temperatures of 100 °C or greater and high catalytic loading [38–43], but some other complexes have comparative working conditions for this reaction [18,44].

**Scheme 1:**
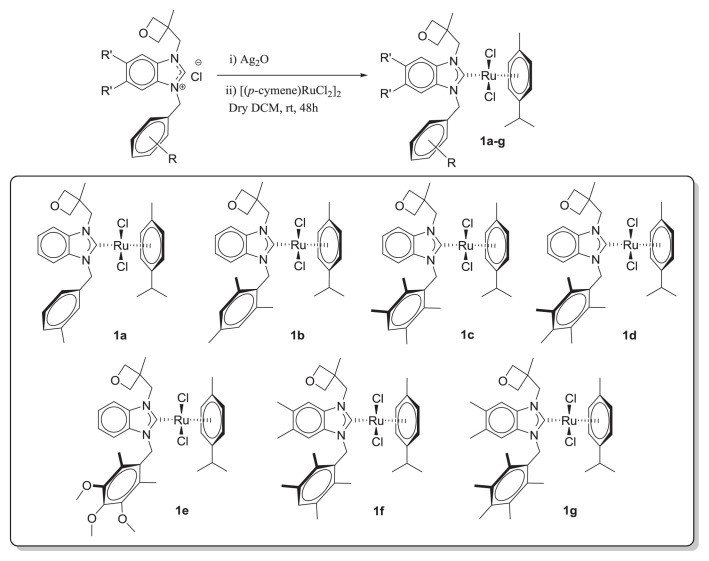
Synthesis of ruthenium *p*-cymene BNHC complexes.

*N*-Heterocyclic carbene (NHC) ligands have become a common alternative to phosphine ligands in homogeneous catalysis over the last 30 years [45–48], especially in combination with ruthenium salts [44,49–52]. We recently described the synthesis of benzimidazolium salts (the precursors to benzimidazole-based NHC (BNHC) ligands) and their silver(I) complexes, which were determined to be active catalysts for carboxylation of epoxides to generate carbonates [53] and aldehyde–amine–alkyne coupling. The preparation and identification of new ruthenium(II) complexes having the general formula [(**η**^6^-*p*-cymene)(BNHC)RuCl_2_] (**1a-g**) are described in the present paper (Scheme 1). The hydrogen borrowing approach was used to test these complexes as catalysts for the *N*-alkylation of anilines and amine-substituted heterocycles with a variety of alcohols.

## 2. Experimental section

### 2.1. Materials and methods

All metal complex preparation methods and catalytic reactions were performed using normal Schlenk procedures. Reagents were bought from commercial sources and were not purified prior to use. The melting point of the produced compounds was determined using open capillary tubes in an Electrothermal 9200 melting point device. A PerkinElmer Spectrum 100 spectrometer with a range of 4000–400 cm^−1^ was utilized for FT-IR analysis. NMR spectra were obtained using a Bruker Ascend 400 Avance III HD, which operated at 400 MHz (^1^H) and 100 MHz (^13^C) using tetramethyl silane as an internal reference. NMR experiments were conducted in high-quality 5-mm Young NMR tubes. Chemical shifts (*δ*) and coupling constants (*J*) are expressed in parts per million (ppm) and hertz (Hz). ^13^C chemical shifts are given relative to deuterated solvents (=77.16 ppm for CDCl_3_). ^1^H NMR spectra are referenced to residual protonated solvents (=7.26 ppm for CDCl_3_).

### 2.2. General synthetic methodologies used for the synthesis of benzimidazol-2-ylidene ruthenium complexes, 1a-g

Complexes [(η^6^-*p*-cymene)(BNHC)RuCl_2_] were synthesized in a one-step process through transmetalation. Dimeric complex of ruthenium [RuCl_2_(*p*-cymene)]_2_ (0.19 mmol) was added to Ag(I)–BNHC complexes (0.383 mmol) in situ without isolation and the mixture was stirred at 25 °C in dichloromethane (DCM) for 48 h. Orange–brown complexes **1a**, **1b**, **1c**, **1d**, **1e**, **1f**, and **1g** of ruthenium carbene were isolated in good yields of 42.5%–80%. Data regarding the ^1^H and ^13^C NMR spectra are given in Tables 1 and 2.

**1a**. Yield: 63%; orange–brown solid: mp 172–174 °C.
**1b**. Yield: 55%; orange–brown solid: mp 172–174 °C.
**1c**. Yield: 67%; orange–brown solid: mp 180–182 °C.
**1d**. Yield: 78%; orange–brown solid: mp 180–182 °C.
**1e**. Yield: 48%; orange–brown solid: mp 298.5–298.7 °C.
**1f**. Yield: 80%; light brown solid: mp 145–148 °C.
**1g**. Yield: 42.5%; dark brown solid: mp 242–243 °C.

#### 2.2.1. X-ray crystallography

X-ray measurements were performed with a STOE IPDS II diffractometer at room temperature using graphite-monochromated MoK**α** radiation by applying the *w*-scan method. Data collection and cell refinement were carried out using X-AREA, while data reduction was applied using X-RED32. The structure was solved by direct methods with SIR2019 [54] and refined by means of the full-matrix least-squares calculations on *F*^2^ using SHELXL-2018 [55]. All H atoms were located in difference maps and then treated as riding atoms, fixing the bond lengths at 0.98, 0.93, 0.97, and 0.96 Å for methine CH, aromatic CH, CH_2_, and CH_3_ atoms, respectively. The displacement parameters of the H atoms were fixed at *U*_iso_(H) = 1.2 *U*_eq_ (1.5 *U*_eq_ for CH_3_). Crystal data, data collection, and structure refinement details are given in Table 3. The molecular graphic was generated using OLEX2 [56].

### 2.3. A general approach: *N*-alkylation of amines with alcohols

At room temperature, compound **1e** (1 mol %), KO^t^Bu (75 mol %), alcohols (1 mmol), and amine (1 mmol) were added to a 15-mL reaction tube in a glove box. The tube was then closed and taken out of the glove box. The reaction mixture was then heated at 120 °C for 12 h with degassed toluene (3 mL). After cooling to room temperature, the reaction mixture was diluted with ethyl acetate, filtered, and vacuum dried. The product was purified using a suitable mixture of petroleum ether and ethyl acetate in column chromatography over silica gel (300–400 mesh) (80:1).

### 2.4. A general approach: aniline *N*-methylation with methanol

In a glove box, amine (1 mmol), MeOH (2 mL), **1e** (1 mol %), and KO^t^Bu were introduced into a 15-mL sealing tube (75 mol %). The tube was then removed from the glove box and sealed with a screw cap. At 110 °C, the reaction mixture was agitated for 12 h. The liquid was diluted with ethyl acetate and filtered through a short pad of silica after cooling to room temperature (2 cm in a Pasteur pipette). Ethyl acetate was used to wash the silica. The crude residue was refined by column chromatography (SiO_2_, petroleum ether:ethyl acetate = 80:1) after the filtrate had evaporated.

## 3. Results and discussion

### 3.1. Preparation of ruthenium(II) complexes

Starting with previously described benzimidazolium salts [53,57], the addition of Ag_2_O followed by [(*p*-cymene)RuCl_2_]_2_ in dry dichloromethane resulted in the formation of the corresponding [(**η**^6^-*p*-cymene)(^R^BNHC^CH2OxMe^)RuCl_2_] (**1a**–**g**) compounds after 48 h at ambient temperature (Scheme 1). A large band was observed in the FT-IR spectra of the free ligands in the 1572–1556 cm^−1^ range, which corresponds to the vibration of the C=N bonds in ligands. In the ruthenium complex, these bands shifted to the 1461–1486 cm^−1^ range, which clearly indicated the shifting of double bond (C=N) character to single bond character **υ**_(NCN)_. The ^1^H NMR spectra of these complexes revealed that the characteristic downfield NC*H*N signal of the salts had disappeared. The methine proton of the *p*-cymene group was located as a septet between 2.97 and 3.02 ppm for the respective complexes, while the methyl protons of the *p*-cymene appeared at 1.15–1.21 ppm. In the ^13^C NMR spectra of complexes **1a**–**g**, the carbene carbon attached to ruthenium gave characteristic signals in the range of 184.9–190.3 ppm (see Tables 1 and 2 and Sup Inf).

### 3.2. Structural analysis

The molecular structures of **1f** and **1g** with complete atom numbering are displayed in the Figure, while important bond distances and angles are listed in Tables 3 and 4. Both structures consist of a BNHC ligand coordinated to a ruthenium center, which also features a *p*-cymene and two chloride ligands in the coordination sphere. Compound **1f** crystallizes in triclinic space group *P*–1 with two molecules in the unit cell, while **1g** crystallizes in orthorhombic space group *Pbca* with eight molecules in the unit cell.

In the structures, the BNHC ligand is coordinated to Ru(II) in a monodentate manner via a neutral carbenic carbon, while the arene ring of *p*-cymene is coordinated to the metal ion in an ***η***^6^-fashion. The complexes can be identified as characteristic three-legged piano stool complexes with a pseudooctahedral geometry that is common for ruthenium half-sandwich arene complexes. Furthermore, the geometry around the metal atoms may be regarded as a tetrahedron with considerable trigonal distortion, if bonding to the p-cymene centroid is considered.

Defining Cg as the centroid of the arene ring, the Ru–Cg distance is 1.7098(11) Å in **1f** and 1.7058(17) Å in **1g**, while the Cl1–Ru1–Cg, Cl2–Ru1–Cg and C1–Ru1–Cg angles are 124.38(4), 127.60(4), and 123.23(7)° in **1f**, and 122.90(7), 126.94(7), and 124.93(12)° in **1g**, respectively. The Cl1–Ru1–Cl2, Cl1–Ru1–C1 and Cl2–Ru1–C1 angles are smaller than the ideal tetrahedral angle (109.47°), which is compensated for by extension of the Cg–Ru–*L* (L is Cl1, Cl2, or C1) angles. The ruthenium atom is bound to the arene ring with a mean Ru–C bond distance of 2.21 Å in both complexes. The Ru1–C1 bond distance is 2.074(2) **Å** in **1f** and 2.096(4) **Å** in **1g**, while the Ru–Cl bonds range from 2.4122(7) to 2.4307(11) Å. The structural data of the complexes are consistent with those of previously reported NHC-Ru(II)(*p*-cymene)Cl_2_ complexes [51,58–63].

### 3.3. Optimization of amine alkylation with alcohols

The ability of synthesized (BNHC)Ru complexes that might promote amine alkylation was then evaluated, as shown in Table 5. In the presence of potassium tert-butoxide, 1.0 mol % of ruthenium complex **1e**, which features meta and para methoxy substitution, was fully benzylated 4-methoxy aniline (>99% conversion, entry) after 12 h at 120 °C to generate secondary amine product **A**. KO*^t^*Bu was an efficient base for obtaining high yields. However, conversion was not possible when substituting weaker bases for KO*^t^*Bu, such as K_2_CO_3_ and Na_2_CO_3_. KO*^t^*Bu was required at 75 mol % to achieve satisfactory conversion. Surprisingly, the reaction still reached 98% conversion at a lower temperature of 70 °C (Table 5, entry 2). However, this trial did lead to the observation of imine product **B** (92:8 **A:B** ratio).

Lowering the temperature even further to 50 °C still allowed 96% conversion with lower selectivity for product **A** (88:12, Table 5, entry 3). A more pronounced loss of selectivity was observed when the catalyst loading was lowered to 0.5 mol % (65:35, Table 5, entry 4). Similarly, stopping a 70 °C reaction after 5 h revealed 98% conversion, but incomplete imine hydrogenation. Compounds **1f** and **1g**, featuring significant methyl substitution, were slightly less effective for this reaction (Table 5, entries 6 and 7). When the reaction was carried out in an open-air environment or in water, conversion was significantly reduced (Table 5, entries 8 and 9).

### 3.4. *N*-Alkylation on aniline with substituted primary alcohols

Encouraged by these findings, the scope of aniline *N*-alkylation under mild conditions (70 °C, 12 h) using **1e** was explored. Table 6 illustrates that both electron-rich and electron-deficient benzylic alcohols worked well, yielding alkylated aniline derivatives **2a**–**j** in 55%–94% isolated yield. Catalysis was compatible with several functional groups, including methoxy groups (**2c** and **2f**), halides (**2a** and **2d**), and trifluoromethyl groups (**2i**). Debromination was observed in the case of *para*-bromobenzyl alcohol, but the brominated product was extracted in a reasonable yield (Table 6, 55%). At 90 °C, the sterically hindered *ortho*-methyl benzylic alcohol and *ortho*-methoxy benzylic alcohol still allow monoalkylated amine products **2e** and **2f** in 90% and 87% yield, respectively. Products **2g** and **2h** were obtained in 65% and 71% yield when heterocyclic alcohols such as 2-furylmethanol and 2-thiophenemethanol were utilized as substrates. Using the aliphatic alcohol heptanol afforded aniline derivative **2j** in 75% isolated yield (Table 6). The *N*-alkylation of anilines with secondary alcohols like 1-phenethyl alcohol, cyclohexanol, and isopropyl alcohol, on the other hand, was ineffective, generating only trace amounts of products. This observation is consistent with a mechanism that involves alcohol dehydrogenation to generate an aldehyde intermediate.

### 3.5. *N*-Alkylation of substituted anilines and heterocyclic amines with benzyl alcohol

The scope of amines that could undergo alkylation was then explored (Table 7). The *N*-alkylated products **3a**–**i** were obtained in good yield (81%–92%) from substrates containing either electron-donating or electron-withdrawing substituents. For example, 1,3-benzodioxan-5-amine was treated with benzyl alcohol to produce **3h** in high yield (Table 7, 87%). Heteroaromatic amines like 2-aminopyridine, 3-aminopyridine, and 2-aminopyrimidine were successfully converted into products **3d**–**f** in good yield (Table 7, 81%–86%). The secondary amine morpholine (**3i**), on the other hand, was not tolerated. The *N*-alkylation of *p*-nitroaniline with benzyl alcohol and the *N*-alkylation of aniline with 4-nitrobenzyl alcohol were also not successful, even with a greater catalyst loading (2 mol %) when conducted at 110 °C. These observations indicate that nitro groups are not tolerated by **1e**.

### 3.6. *N*-Methylation of anilines

*N*-Methylamines are commonly employed as intermediates and building blocks in the production of bulk and fine chemicals, as well as materials [64,65]. Due to the higher activation barrier (21 kcal mol^−1^) of methanol dehydrogenation compared to that of higher alcohols, such as ethanol (16 kcal mol^−1^), methanol can be a problematic substrate for the *N*-alkylation of amines [66]. Therefore, the *N*-methylation of amines with methanol was examined to further broaden the scope of **1e** promoted C–N bond formation. To our delight, we were able to successfully *N*-methylate anilines with methanol in the presence of 1.0 mol % of **1e** at 110 °C (Table 8). As indicated in Table 8, the majority of the catalytic reactions were efficient, yielding at least 81% of the desired product (Table 8, **4a**–**g** in 46%–97% yield). When 2-iodoaniline was used, the reaction produced **5g** in moderate isolated yields (46%) and was also dehalogenated. Biologically important motifs like pyridine-2-amine and 3-trifluoromethylaniline were also successfully methylated (Table 8, **4d** and **4e**). Despite the use of copious MeOH and high temperatures, we did not observe dialkylation products in any case. Attempts to obtain *N*-methylate aliphatic amines like benzylamine and *n*-hexylamine, on the other hand, were ineffective, giving just traces of methylated product.

A comparison of the most active catalyst (**1e**) with other reported catalysts is shown in Table 9. It is the best Ru–NHC-based catalyst in terms of low catalyst loading (1 mol %) and low temperature (70 **°C**). Furthermore, most of the literature reports that the hydrogen transfer reaction is used to convert the alcohol into ketone or aldehyde using Ru–NHC complexes. Very few reports of such secondary amine products through the coupling of primary amine and alcohols have been reported using ruthenium–NHC complexes. These Ru–NHC complexes are also applicable for the conversion of highly complicated products like methanol and convert them into secondary amines.

### 3.7. Proposed mechanism

**Scheme 2:**
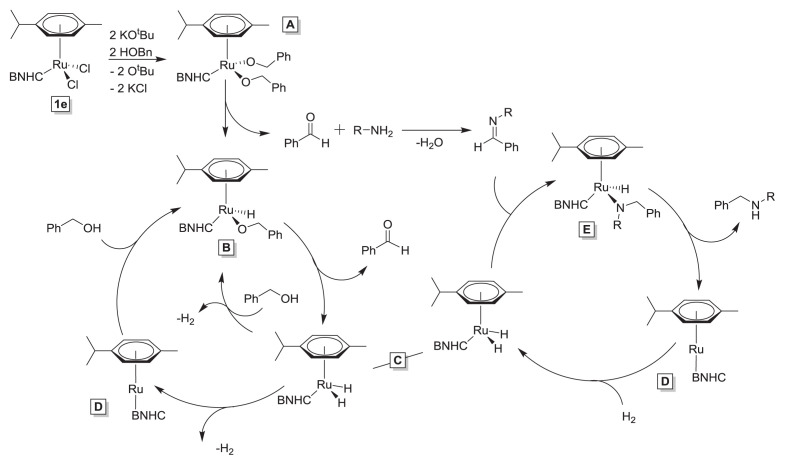
A plausible mechanism for C–N bond formation catalyzed by **1e**.

Given that catalysis requires the presence of KO*^t^*Bu (75 mol % relative to 1.0 mol % of **1e**), it is reasonable to propose that a salt metathesis reaction occurs to form the corresponding bis(*tert*-butoxide) complex, which can undergo σ-bond metathesis with incoming benzyl alcohol to generate intermediate **A** (Scheme 2).

Alternatively, intermediate **A** may be generated directly from **1e** if any KOBn is generated in solution. This intermediate can undergo subsequent β-hydride elimination steps to generate **B** and ultimately dihydride complex **C**. As has been observed for other hydrogen borrowing C–N bond forming reactions, the in situ generation of aldehyde results in Schiff base condensation with any primary amine that is present to generate the corresponding aldimine (this is also the reason why secondary amines do not become alkylated again to yield tertiary amines). This aldimine can insert into dihydride **C** to generate intermediate **E**. At this point, the desired secondary amine product can be liberated in one of two ways. Reductive elimination from **E** can occur to generate intermediate **D** (as shown in Scheme 2) or **E** can undergo σ-bond metathesis with the next equivalent of benzyl alcohol to generate intermediate **B**. If Ru(0) intermediate **D** is formed during the reaction, it quickly reacts with any alcohol or hydrogen that is present to generate the corresponding hydride complex.

## 4. Conclusion

We have described the synthesis and characterization of a series of ruthenium complexes with BNHC proligands that feature a variety of benzyl group substitution patterns. Through a HB/HA mechanism, these compounds were discovered to be highly effective catalysts for the selective monoalkylation of aromatic primary amines. Complex **1e** was the most active of the catalysts tested, and it is one of the most active ruthenium catalysts ever reported for amine alkylation given that it operates efficiently at temperatures as low as 50 °C at low catalyst loadings (1.0 mol %). A wide range of (hetero)aromatic amines and primary alcohols were successfully converted into secondary amines in good to exceptional isolated yields, including physiologically relevant examples. The methylation of primary amines was also achieved using methanol, a transformation that is particularly difficult to demonstrate.

## Supporting Information

### Characterizing data of ruthenium–BNHC complex 1a

#### Dichloro-[1-((3-methyloxetan-3-yl)methyl)-3-(3-methylbenzyl)benzimidazole-2-ylidene](*p*-cymene) ruthenium(II) (1a)

Figure S1 ^1^H NMR spectrum of ruthenium–BNHC complex **1a** (in CDCl_3_, 25 **°**C, TMS, 400 MHz).

Figure S2 ^13^C NMR spectrum of ruthenium–BNHC complex **1a** (in CDCl_3_, 25 **°**C, TMS, 101 MHz).

Figure S3 FT-IR spectrum of ruthenium–BNHC complex **1a**.

### Characterizing data of ruthenium–BNHC complex 1b

#### Dichloro-[1-((3-methyloxetan-3-yl)methyl)-3-(2,4,6-trimethylbenzyl)benzimidazole-2-ylidene](*p*-cymene) ruthenium(II) (1b)

Figure S4 ^1^H NMR spectrum of ruthenium–BNHC complex **1b** (in CDCl_3_, 25 **°**C, TMS, 400 MHz).

Figure S5 ^13^C NMR spectrum of ruthenium–BNHC complex **1b** (in CDCl_3_, 25 **°**C, TMS, 101 MHz).

Figure S6 FT-IR spectrum of ruthenium–BNHC complex **1b**.

### Characterizing data of ruthenium–BNHC complex 1c

#### Dichloro-[1-((3-methyloxetan-3-yl)methyl)-3-(2,3,5,6-tetramethylbenzyl)benzimidazole-2-ylidene](*p*-cymene) ruthenium(II) (1c)

Figure S7 ^1^H NMR spectrum of ruthenium–BNHC complex **1c** (in CDCl_3_, 25 **°**C, TMS, 400 MHz).

Figure S8 ^13^C NMR spectrum of ruthenium–BNHC complex **1c** (in CDCl_3_, 25 **°**C, TMS, 101 MHz).

Figure S9 FT-IR spectrum of ruthenium–BNHC complex **1c**.

### Characterizing data of ruthenium–BNHC complex 1d

#### Dichloro-[1-((3-methyloxetan-3-yl)methyl)-3-(2,3,4,5,6-pentamethylbenzyl)benzimidazole-2-ylidene](*p*-cymene) ruthenium(II) (1d)

Figure S10 ^1^H NMR spectrum of ruthenium–BNHC complex **1d** (in CDCl_3_, 25 **°**C, TMS, 400 MHz).

Figure S11 ^13^C NMR spectrum of ruthenium–BNHC complex **1d** (in CDCl_3_, 25 **°**C, TMS, 101 MHz).

Figure S12 FT-IR spectrum of ruthenium–BNHC complex **1d**.

### Characterizing data of ruthenium–BNHC complex 1e

#### Dichloro-[1-((3-methyloxetan-3-yl)methyl)-3-(3,4,5-trimethoxybenzyl)benzimidazole-2-ylidene](*p*-cymene) ruthenium(II) (1e)

Figure S13 ^1^H NMR spectrum of ruthenium–BNHC complex **1e** (in CDCl_3_, 25 **°**C, TMS, 400 MHz).

Figure S14 ^13^C NMR spectrum of ruthenium–BNHC complex **1e** (in CDCl_3_, 25 **°**C, TMS, 101 MHz).

Figure S15 FT-IR spectrum of ruthenium–BNHC complex **1e**.

### Characterizing data of ruthenium–BNHC complex 1f

#### Dichloro-[(5,6-dimethyl-1-((3-methyloxetan-3-yl)methyl)-3-(2,3,5,6-tetramethylbenzyl)benzimidazole-2-ylidene](*p*-cymene) ruthenium(II) (1f)

Figure S16 ^1^H NMR spectrum of ruthenium–BNHC complex **1f** (in CDCl_3_, 25 **°**C, TMS, 400 MHz).

Figure S17 ^13^C NMR spectrum of ruthenium–BNHC complex **1f** (in CDCl_3_, 25 **°**C, TMS, 101 MHz).

Figure S18 FT-IR spectrum of ruthenium–BNHC complex **1f**.

### Characterizing data of ruthenium–BNHC complex 1g

#### Dichloro-[(5,6-dimethyl-1-((3-methyloxetan-3-yl)methyl)-3-(2,3,4,5,6-pentamethylbenzyl)benzimidazole-2-ylidene](*p-*cymene) ruthenium(II) (1g)

Figure S19 ^1^H NMR spectrum of ruthenium–BNHC complex **1g** (in CDCl_3_, 25 **°**C, TMS, 400 MHz).

Figure S20 ^13^C NMR spectrum of ruthenium–BNHC complex **1g** (in CDCl_3_, 25 **°**C, TMS, 101 MHz).

Figure S21 FT-IR spectrum of ruthenium–BNHC complex **1g**.

### Characterizing data of the tested (2a–h) substituted benzyl alcohol with aniline by the complex 1e

#### *N*-(4-chlorobenzyl)aniline (2a)

^1^H NMR (400 MHz, CDCl_3_) δ 7.32 (s, 4H), 7.20 (dd, *J* = 8.4, 7.5 Hz, 2H), 6.76 (t, *J* = 7.3 Hz, 1H), 6.66–6.57 (m, 2H), 4.32 (s, 2H), 4.05 (s, 1H).

^13^C NMR (101 MHz, CDCl_3_) δ 147.9, 138.0, 132.9, 129.3, 128.7, 117.8, 112.9, 47.6.

Figure S22 ^1^H NMR and ^13^C NMR spectrum of 2**a** (in CDCl_3_, 25 **°**C, TMS, 400 MHz).

#### *N*-(4-methylbenzyl)aniline (2b)

^1^H NMR (400 MHz, CDCl_3_) δ 7.76 (d, *J* = 7.4 Hz, 2H), 7.68 (d, *J* = 11.1 Hz, 4H), 7.21 (t, *J* = 7.2 Hz, 1H), 7.13 (d, *J* = 7.7 Hz, 2H), 4.77 (s, 2H), 4.31 (s, 1H), 2.85 (s, 3H). ^13^C NMR (101 MHz, CDCl_3_) δ 148.7, 137.3, 136.8, 129.7, 128.0, 117.9, 113.3, 48.5, 21.6.

Figure S23 ^1^H NMR and ^13^C NMR spectrum of **2b** (in CDCl_3_, 25 **°**C, TMS, 400 MHz).

#### *N*-(4-methoxybenzyl)aniline (2c)

^1^H NMR (400 MHz, CDCl_3_) δ 7.33 (d, *J* = 8.6 Hz, 2H), 7.26–7.17 (m, 2H), 6.93 (dd, *J* = 9.2, 2.4 Hz, 2H), 6.76 (t, *J* = 7.3 Hz, 1H), 6.67 (d, *J* = 7.7 Hz, 2H), 4.28 (s, 2H), 3.98 (s, 1H), 3.83 (s, 3H). ^13^C NMR (101 MHz, CDCl_3_) δ 158.9, 148.3, 131.5, 129.3, 128.8, 117.5, 114.1, 112.9, 55.3, 47.8.

Figure S24 ^1^H NMR and ^13^C NMR spectrum of **2c** (in CDCl_3_, 25 **°**C, TMS, 400 MHz).

#### *N*-(4-bromobenzyl)aniline (2d)

^1^H NMR (400 MHz, CDCl_3_) δ 7.43 (dd, *J* = 6.8, 1.5 Hz, 2H), 7.21 (d, *J* = 7.3 Hz, 2H), 7.15 (tt, *J* = 7.2, 1.7 Hz, 2H), 6.74–6.68 (m, 1H), 6.62–6.53 (m, 2H), 4.25 (s, 2H), 3.92 (s, 1H). ^13^C NMR (101 MHz, CDCl_3_) δ 145.9, 136.0, 130.9, 127.3, 126.7, 115.8, 110.9, 45.6.

Figure S25 ^1^H NMR and ^13^C NMR spectrum of **2d** (in CDCl_3_, 25 **°**C, TMS, 400 MHz).

#### *N*-(2-methylbenzyl)aniline (2e)

^1^H NMR (400 MHz, CDCl_3_) δ 7.25 (dt, *J* = 14.9, 7.9 Hz, 5H), 7.13 (d, *J* = 7.3 Hz, 1H), 6.76 (t, *J* = 7.3 Hz, 1H), 6.68 (d, *J* = 7.8 Hz, 2H), 4.32 (s, 2H), 4.02 (s, 1H), 2.39 (s, 3H). ^13^C NMR (101 MHz, CDCl_3_) δ 148.3, 139.4, 138.3, 129.3, 128.6, 128.3, 128.0, 124.6, 117.5, 112.9, 48.4, 21.5.

Figure S26 ^1^H NMR and ^13^C NMR spectrum of **2e** (in CDCl_3_, 25 **°**C, TMS, 400 MHz).

#### *N*-(2-methoxybenzyl)aniline (2f)

^1^H NMR (400 MHz, CDCl_3_) δ 7.26 (ddd, *J* = 14.8, 11.4, 4.3 Hz, 3H), 7.00 (d, *J* = 13.3 Hz, 2H), 6.87 (d, *J* = 8.1 Hz, 1H), 6.77 (dd, *J* = 10.4, 4.2 Hz, 1H), 6.68 (d, *J* = 7.5 Hz, 2H), 4.34 (s, 2H), 3.98 (s, 1H), 3.83 (s, 3H). ^13^C NMR (101 MHz, CDCl_3_) δ 160.0, 148.2, 141.3, 129.7, 129.3, 119.8, 117.6, 113.0, 112.7, 55.2, 48.3.

Figure S27 ^1^H NMR and ^13^C NMR spectrum of **2f** (in CDCl_3_, 25 **°**C, TMS, 400 MHz).

#### *N*-(furan-2-ylmethyl)aniline (2g)

^1^H NMR (400 MHz, CDCl_3_) δ 7.40–7.33 (m, 1H), 7.22–7.15 (m, 2H), 6.77–6.72 (m, 1H), 6.68 (ddd, *J* = 4.6, 2.1, 1.1 Hz, 2H), 6.36–6.29 (m, 1H), 6.23 (dd, *J* = 3.2, 0.8 Hz, 1H), 4.32 (s, 2H), 3.95 (s, 1H). ^13^C NMR (101 MHz, CDC_3_) δ 152.7, 147.6, 141.9, 129.2, 118.0, 113.1, 110.3, 106.9, 41.4.

Figure S28 ^1^H NMR and ^13^C NMR spectrum of **2g** (in CDCl_3_, 25 **°**C, TMS, 400 MHz).

#### *N*-(3,5-bis(trifluoromethyl)benzyl)aniline (2h)

^1^H NMR (400 MHz, CDCl_3_) δ 7.84 (s, 2H), 7.79 (s, 1H), 7.18 (td, *J* = 7.8, 0.7 Hz, 2H), 6.77 (td, *J* = 7.4, 0.8 Hz, 1H), 6.60 (dd, *J* = 7.7, 0.8 Hz, 2H), 4.47 (s, 2H), 4.18 (s, 1H). ^13^C NMR (101 MHz, CDCl_3_) δ 147.2, 142.5, 129.4, 118.5, 113.0, 47.7.

Figure S29 ^1^H NMR and ^13^C NMR spectrum of **2h** (in CDCl_3_, 25 **°**C, TMS, 400 MHz).

### Characterizing data of the tested (3a–h) substituted aniline with benzyl alcohol by the complex 1e

#### *N*-benzyl-4-chloroaniline (3a)

^1^H NMR (400 MHz, CDCl_3_) δ 7.34–7.30 (m, 4H), 7.26 (dd, *J* = 8.8, 4.6 Hz, 1H), 7.10–7.03 (m, 2H), 4.26 (d, *J* = 5.5 Hz, 2H), 4.02 (s, 1H). ^13^C NMR (101 MHz, CDCl_3_) δ 147.9, 138.0, 132.9, 129.3, 128.7, 117.8, 112.9, 47.6.

Figure S30 ^1^H NMR and ^13^C NMR spectrum of **3a** (in CDCl_3_, 25 **°**C, TMS, 400 MHz).

#### *N*-benzyl-4-methylaniline (3b)

^1^H NMR (400 MHz, CDCl_3_) δ 7.41–7.19 (m, 5H), 6.96 (d, *J* = 8.3 Hz, 2H), 6.53 (d, *J* = 8.4 Hz, 2H), 4.27 (s, 2H), 3.86 (s, 1H), 2.22 (s, 3H). ^13^C NMR (101 MHz, CDCl_3_) δ 146.0, 139.7, 129.8, 128.6, 127.5, 127.2, 113.0, 48.7, 20.4.

Figure S31 ^1^H NMR and ^13^C NMR spectrum of **3b** (in CDCl_3_, 25 **°**C, TMS, 400 MHz).

#### *N*-benzyl-4-methoxyaniline (3c)

^1^H NMR (400 MHz, CDCl_3_) δ 7.37 (dt, *J* = 20.8, 10.2 Hz, 4H), 7.31–7.24 (m, 1H), 6.81 (d, *J* = 8.8 Hz, 2H), 6.63 (d, *J* = 8.8 Hz, 2H), 4.30 (s, 2H), 3.76 (s, 4H). ^13^C NMR (101 MHz, CDCl_3_) δ 152.2, 142.4, 139.7, 128.6, 127.6, 114.9, 114.2, 55.8, 49.3.

Figure S32 ^1^H NMR and ^13^C NMR spectrum of **3c** (in CDCl_3_, 25 **°**C, TMS, 400 MHz).

#### *N*-benzylpyridin-2-amine (3d)

^1^H NMR (400 MHz, CDCl_3_) δ 8.10–8.00 (m, 1H), 7.46–7.28 (m, 5H), 7.27–7.21 (m, 1H), 6.55 (ddd, *J* = 7.1, 5.0, 0.9 Hz, 1H), 6.33 (d, *J* = 8.4 Hz, 1H), 5.16 (s, 1H), 4.47 (d, *J* = 5.8 Hz, 2H). ^13^C NMR (101 MHz, CDCl_3_) δ 158.7, 148.2, 139.2, 128.6, 127.3, 113.1, 106.7, 46.3.

Figure S33 ^1^H NMR and ^13^C NMR spectrum of **3d** (in CDCl_3_, 25 **°**C, TMS, 400 MHz).

#### *N*-benzylpyridin-3-amine (3e)

^1^H NMR (400 MHz, CDCl_3_) δ 8.04 (d, *J* = 2.9 Hz, 1H), 7.94 (dd, *J* = 4.7, 1.2 Hz, 1H), 7.34 (d, *J* = 4.4 Hz, 4H), 7.30–7.24 (m, 1H), 7.04 (dd, *J* = 8.3, 4.7 Hz, 1H), 6.85 (ddd, *J* = 8.3, 2.8, 1.2 Hz, 1H), 4.32 (s, 2H), 4.25 (s, 1H). ^13^C NMR (101 MHz, CDCl_3_) δ 144.0, 138.8, 138.5, 136.1, 128.7, 127.4, 123.7, 118.5, 47.8.

Figure S34 ^1^H NMR and ^13^C NMR spectrum of **3e** (in CDCl_3_, 25 **°**C, TMS, 400 MHz).

#### *N*-benzylpyrimidin-2-amine (3f)

^1^H NMR (400 MHz, CDCl_3_) δ 8.19 (d, *J* = 3.3 Hz, 2H), 7.33 (q, *J* = 7.9 Hz, 4H), 7.28–7.23 (m, 1H), 6.50 (t, *J* = 4.8 Hz, 1H), 5.96 (s, 1H), 4.62 (d, *J* = 5.9 Hz, 2H). ^13^C NMR (101 MHz, CDCl_3_) δ 158.0, 139.1, 128.6, 127.5, 127.2, 110.7, 45.4.

Figure S35 ^1^H NMR and ^13^C NMR spectrum of **3f** (in CDCl_3_, 25 **°**C, TMS, 400 MHz).

#### *N*-benzylbenzo[1,3]dioxol-5-amine (3g)

^1^H NMR (400 MHz, CDCl_3_) δ 7.40–7.33 (m, 4H), 7.30–7.25 (m, 1H), 6.66 (d, *J* = 8.2 Hz, 1H), 6.27 (d, *J* = 2.3 Hz, 1H), 6.07 (dd, *J* = 8.3, 2.3 Hz, 1H), 5.84 (d, *J* = 0.5 Hz, 2H), 4.26 (s, 2H), 3.67 (s, 1H). ^13^C NMR (101 MHz, CDCl_3_) δ 148.3, 143.9, 139.7, 139.4, 128.6, 127.5, 127.2, 108.6, 104.4, 100.6, 96.0, 49.2.

Figure S36 ^1^H NMR and ^13^C NMR spectrum of **3g** (in CDCl_3_, 25 **°**C, TMS, 400 MHz).

#### *N*-benzyl-3,5-bis(trifluoromethyl)aniline (3h)

^1^H NMR (400 MHz, CDCl_3_) δ 7.43–7.31 (m, 5H), 7.17 (s, 1H), 6.97 (s, 2H), 4.45 (s, 1H), 4.36 (d, *J* = 5.4 Hz, 2H). ^13^C NMR (101 MHz, CDCl_3_) δ 148.6, 137.6, 128.9, 127.8, 127.5, 111.9, 111.0, 48.0.

Figure S37 ^1^H NMR and ^13^C NMR spectrum of **3h** (in CDCl_3_, 25 **°**C, TMS, 400 MHz).

### Characterizing data of the tested (4a–e) substituted aniline with methanol by the complex 1e

#### *N*-methylaniline (4a)

^1^H NMR (400 MHz, CDCl_3_) δ 6.72 (t, *J* = 7.8 Hz, 2H), 6.24 (t, *J* = 7.3 Hz, 1H), 6.15 (d, *J* = 7.8 Hz, 2H), 3.21 (s, 1H), 2.36 (s, 3H).

Figure S38 ^1^H NMR and ^13^C NMR spectrum of **4a** (in CDCl_3_, 25 **°**C, TMS, 400 MHz).

#### *N*,4-dimethylaniline (4b)

^1^H NMR (400 MHz, CDCl_3_) δ 7.02 (d, *J* = 8.5 Hz, 2H), 6.56 (d, *J* = 8.3 Hz, 2H), 3.44 (s, 1H), 2.82 (s, 3H), 2.26 (s, 3H).

Figure S39 ^1^H NMR spectrum of **4b** (in CDCl_3_, 25 **°**C, TMS, 400 MHz).

#### 4-Methoxy-*N*-methylaniline (4c)

^1^H NMR (400 MHz, CDCl_3_) δ 6.82–6.74 (m, 2H), 6.63–6.55 (m, 2H), 3.74 (s, 3H), 2.79 (s, 3H), 0.82 (s, 1H).

Figure S40 ^1^H NMR spectrum of **4c** (in CDCl_3_, 25 **°**C, TMS, 400 MHz).

#### Chloro-*N*-methylaniline (4d)

^1^H NMR (400 MHz, CDCl_3_) δ 6.11 (dd, *J* = 4.5, 3.8 Hz, 2H), 5.50 (d, *J* = 8.1 Hz, 2H), 2.62 (s, 1H), 1.78 (s, 3H).

Figure S41 ^1^H NMR spectrum of **4d** (in CDCl_3_, 25 **°**C, TMS, 400 MHz).

#### *N*-methylpyridin-2-amine (4e)

^1^H NMR (400 MHz, CDCl_3_) δ 7.98 (d, *J* = 2.8 Hz, 1H), 7.91 (dd, *J* = 4.7, 1.1 Hz, 1H), 7.05 (dd, *J* = 8.3, 4.7 Hz, 1H), 6.82 (ddd, *J* = 8.3, 2.8, 1.3 Hz, 1H), 3.80 (dd, *J* = 14.9, 10.1 Hz, 1H), 2.80 (s, 3H).

Figure S42 ^1^H NMR spectrum of **4e** (in CDCl_3_, 25 **°**C, TMS, 400 MHz).

#### *N*-methyl-3,5-bis(trifluoromethyl)aniline (4f)

^1^H NMR (400 MHz, CDCl_3_) δ 7.13 (s, 1H), 6.91 (s, 2H), 4.16 (s, 1H), 2.89 (d, *J* = 5.0 Hz, 3H).

Figure S43 ^1^H NMR spectrum of **4f** (in CDCl_3_, 25 **°**C, TMS, 400 MHz).

## Figures and Tables

**Figure:**
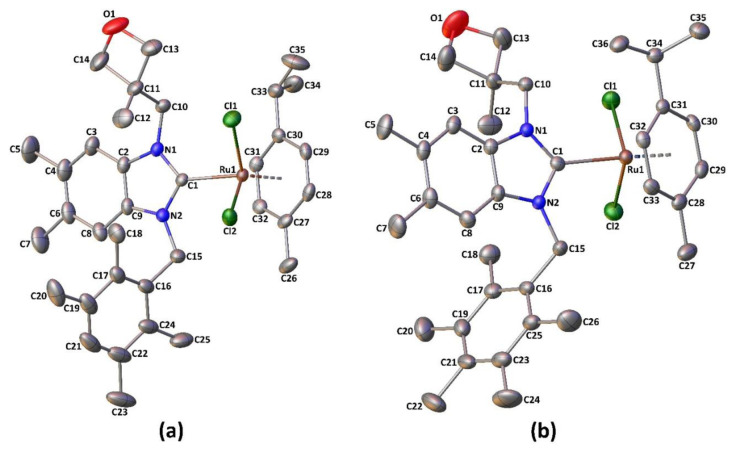
Molecular structures of **1f** (a) and **1g** (b) drawn at the 30% probability level. H atoms have been omitted for clarity.

**Table 1:** Selected ^1^H NMR data for **1**.

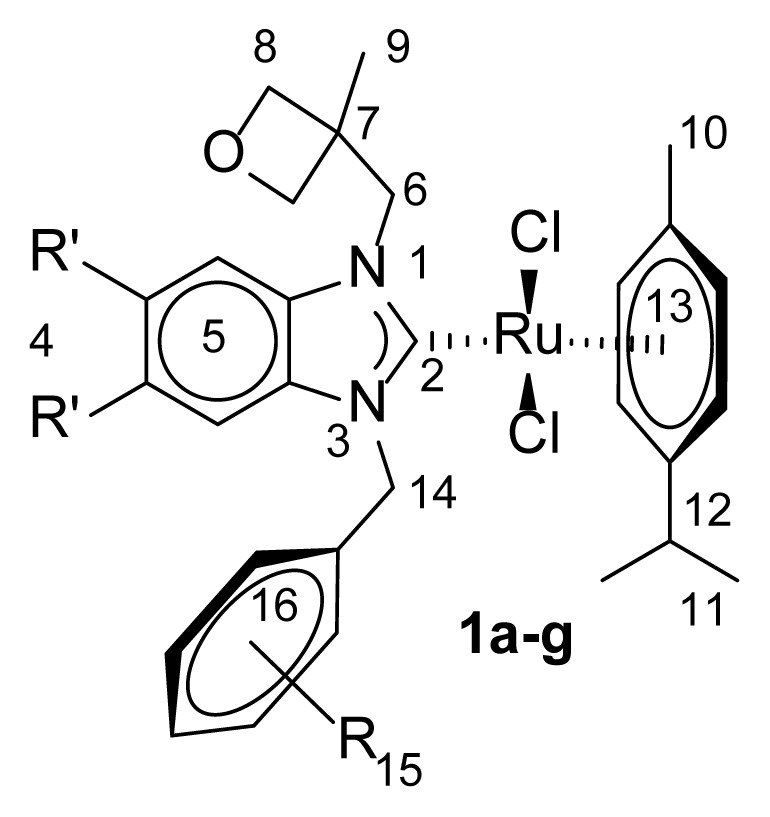												
Comp.	4	5	6	8	9	10	11	12	13	14	15	16
**1a**	-	7.09 (t) 7.18 (td), 7.24– 7.40 (m)	4.50 (s)	4.74–5.05 (m)	1.98 (s)	1.15 (s)	1.25 (d)	2.93 (hept)	5.86–5.72 (m)	5.41 and 5.27 (s)	2.21 (s)	6.98 (d), 6.80 (d) 6.39(d)
**1b**	-	7.19 (dd), 7.04 (d) 7.01–6.93 (m)	5.13 (s)	4.73 and 4.39 (d)	1.88 (s)	1.22 (s)	1.32 (d)	3.03 (hept)	6.68 (s), 6.57 (d), 5.72 (d), 5.43–5.31 (m)	5.52 (d)	2.49(s), 2.25(s) 1.80 and 1.71 (s)	6.57 (s) 6.68 (s)
**1c**	-	7.16 (t) 7.02 (d) 6.97 (s) 6.91 (t)	5.17 (d)	4.75 and 4.39 (d)	1.79 (s)	1.21 (s)	1.32 (d)	3.02 (hept)	6.36 and 5.53 (d) 5.71 (s)	5.44 and 5.53 (s)	2.34, 2.06, and 1.71 (s)	7.22 (s)
**1d**		7.21–7.07 (m) 6.99 (d), 6.94–6.83 (m)	5.18 (d)	4.76 and 4.40 (d)	1.95 (s)	1.19 (s)	1.32 (d)	3.02 (hept)	6.35 (d), 5.70 (s) 5.44 (s), 4.44 (s)	5.53 (d)	2.47–1.77 (m)	–
**1e**		7.32–7.23 (m), 7.22–7.15 (m), 7.06 (d)	5.03 (d)	4.79 and 4.44 (d)	1.91 (s)	1.15 (s)	1.28 (d)	3.02 (hept)	6.26 (d), 5.78 (m), 4.54 (d)	5.43 (d)	3.80 and 3.70 (s)	6.43 (s)
**1f**	2.28 and 2.02 (s)	7.16 and 6.98 (s)	5.13 (d)	4.74 and 4.37 (d)	1.90 (s)	1.21 (s)	1.31 (d)	3.02 (hept)	6.05, 5.63 (s), 5.36 (d)	5.51 (d)	2.45–2.21 (m), 1.16–1.81(m)	6.77 (s)
**1g**	2.27 and 2.28 (s)	7.22–6.92 (m), 6.74 (s)	5.14 (d)	4.75 and 4.38 (d)	2.00 (s)	1.19 (s)	1.31 (d)	3.01 (hept)	6.01, 5.63 (s), 5.46–5.31 (m)	5.51 (d)	2.44–2.29 (m) 2.16–2.01 (m) 1.92 (s)	–

**Table 2:** Selected ^13^C NMR data for **1**.

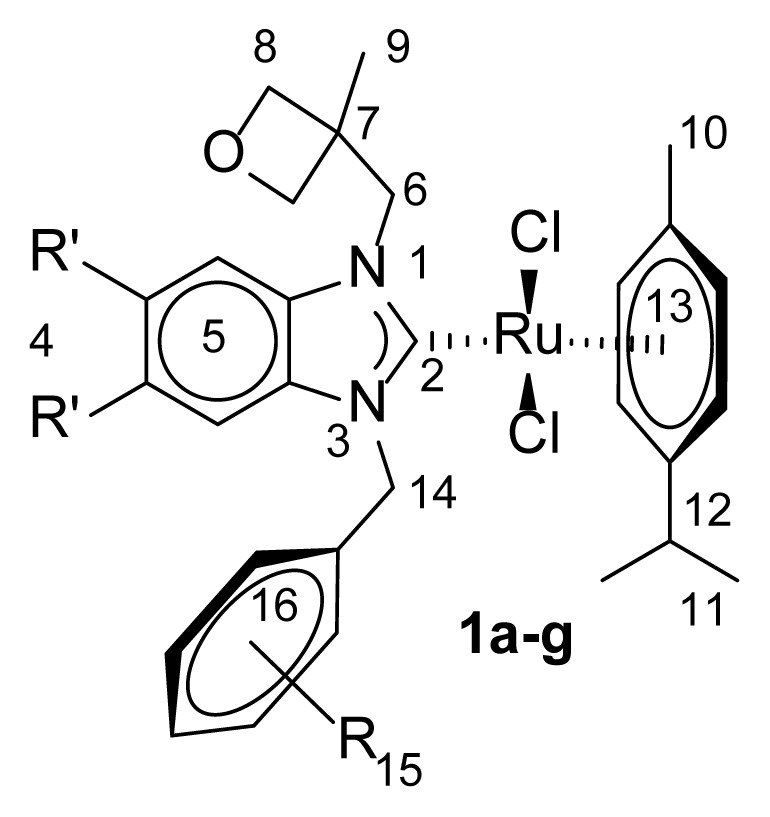												
Comp.	2	4	5,13,16	6	7	8	9	10	11	12	14	15
**1a**	190.3	-	138.7, 137.2, 135.6, 135.5, 128.8, 128.3, 126.7, 122.9, 112.0, 109.3	52.7	40.3	98.9	21.5	18.5	21.7	30.7	55.5	20.6
**1b**	187.7	-	137.4, 135.9, 135.4, 128.5, 111.5, 109.9, 109.6	50.0	40.8	98.6	21.3	18.5	21.6	30.7	54.2	20.9
**1c**	187.5	-	135.9, 135.4, 131.9, 131.5, 122.9, 122.8, 111.8, 109.7, 109.5	50.8	40.8	98.5	21.2	18.6	23.2	30.7	54.2	20.9, 20.5, 16.2
**1d**	187.5	-	135.9, 135.5, 135.2, 128.9, 122.9, 122.6, 112.0, 109.6, 109.5	51.4	40.8	98.5	21.1	18.6	21.1	30.8	54.4	17.2
**1e**	189.7	-	153.4, 137.3, 135.7, 135.4, 132.4, 123.4, 112.0, 110.4, 109.9, 104.0	53.3	40.7	98.6	20.7	18.6	21.3	30.7	54.6	60.9, 56.1
**1f**	184.9	20.4, 20.3	134.6, 134.1, 131.8, 131.7, 109.8, 109.6, 98.6	50.6	40.9	98.6	21.3	18.5	21.2	30.6	53.9	20.4, 20.3
**1g**	184.9	20.4, 20.3	135.1, 134.6, 134.2, 131.6, 131.6, 129.0, 112.5, 109.8, 109.5	51.1	40.8	98.6	21.2	18.5	21.3	30.7	54.1	20.4, 20.3, 17.2

**Table 3:** Crystal data and structure refinement parameters for **1f** and **1g**.

Parameters	1f	1g
CCDC depository	2085163	2173756
Color/shape	Dark red/prism	Light brown/prism
Chemical formula	[RuCl_2_(C_10_H_14_)(C_25_H_32_N_2_O)]	[RuCl_2_(C_10_H_14_)(C_26_H_34_N_2_O)]
Formula weight	682.71	696.73
Temperature (K)	296(2)	296(2)
Wavelength (Å)	0.71073 Mo K*α*	0.71073 Mo K*α*
Crystal system	Triclinic	Orthorhombic
Space group	*P*–1 (No. 2)	*Pbca* (No. 61)
Unit cell parameters		
*a*, *b*, *c* (Å)	7.1553(5), 15.4318(12), 15.5762(12)	7.2829(2), 21.3438(5), 43.0193(13)
*α*, *β*, *γ* (°)	86.525(6), 85.527(6), 77.055(6)	90, 90, 90
Volume (Å^3^)	1669.4(2)	6687.1(3)
*Z*	2	8
*D*_calc._ (g/cm^3^)	1.358	1.384
μ (mm^−1^)	0.659	0.659
Absorption correction	Integration	Integration
*T*_min._, *T*_max._	0.7919, 0.9579	0.8533, 0.9667
*F*_000_	712	2912
Crystal size (mm^3^)	0.48 × 0.23 × 0.09	0.40 × 0.09 × 0.05
Diffractometer/measurement method	STOE IPDS II/ω scan	STOE IPDS II/ω scan
Index ranges	−9 ≤ *h* ≤ 9, −20 ≤ *k* ≤ 20, −20 ≤ *l* ≤ 20	−8 ≤ *h* ≤ 8, −25 ≤ *k* ≤ 25, −52 ≤ *l* ≤ 52
*θ* range for data collection (°)	1.928 ≤ *θ* ≤ 27.676	1.894 ≤ *θ* ≤ 25.646
Reflections collected	26,948	48,873
Independent/observed reflections	7728/6314	6304/3620
*R*_int._	0.0768	0.0927
Refinement method	Full-matrix least-squares on *F*^2^	Full-matrix least-squares on *F*^2^
Data/restraints/parameters	7728/0/380	6304/0/385
Goodness-of-fit on *F*^2^	0.999	0.906
Final *R* indices [*I* > 2*σ*(*I*)]	*R*_1_ = 0.0362, *wR*_2_ = 0.0727	*R*_1_ = 0.0417, *wR*_2_ = 0.0708
*R* indices (all data)	*R*_1_ = 0.0519, *wR*_2_ = 0.0768	*R*_1_ = 0.1006, *wR*_2_ = 0.0833
Δρ_max._, Δρ_min._ (e/Å^3^)	0.56, −0.34	0.37, −0.40

**Table 4:** Selected geometric parameters for **1f** and **1g**.

Parameters	1f	1g	Parameters	1f	1g
***Bond lengths (Å)***			***Bond angles (°)***		
Ru1–Cg	1.7098(11)	1.7058(17)	Cl1–Ru1–Cl2	84.35(3)	84.05(4)
Ru1–Cl1	2.4122(7)	2.4193(11)	Cl1–Ru1–C1	95.53(7)	95.76(10)
Ru1–Cl2	2.4288(7)	2.4307(11)	Cl1–Ru1–Cg	124.38(4)	122.90(7)
Ru1–C1	2.074(2)	2.096(4)	Cl1–Ru1–C_arene_	88.20(7)–158.11(7)	85.23(11)–159.05(10)
Ru1–C_arene_	2.161(2)–2.249(2)	2.194(4)–2.240(4)	Cl2–Ru1–C1	90.89(7)	91.24(10)
N1–C1	1.361(3)	1.364(5)	Cl2–Ru1–Cg	127.60(4)	126.94(7)
N2–C1	1.370(3)	1.366(4)	Cl2–Ru1–C_arene_	91.24(7)–156.33(6)	89.88(11)–157.66(11)
			C1–Ru1–Cg	123.23(7)	124.93(12)
			C1–Ru1–C_arene_	86.78(9)–153.24(10)	87.70(14)–157.11(15)
			N1–C1–N2	105.29(18)	104.7(3)

Note: Cg represents the centroid of the arene ring.

**Table 5:** The use of benzyl alcohol to optimize the *N*-alkylation of 4-methoxyaniline.

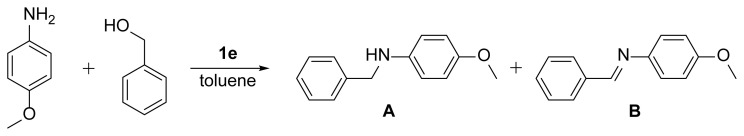						
Entry	Cat (mol %)	Base (75 mol %)	Temp (°C)	Time (h)	Conversion (%)	**A**/**B**
1	**1e** (1.0)	KO*^t^*Bu	120	12	> 99	>99/0
2	**1e** (1.0)	KO*^t^*Bu	70	12	98	92/8
3	**1e** (1.0)	KO*^t^*Bu	50	12	96	88/12
4	**1e** (0.5)	KO*^t^*Bu	70	12	96	65/35
5	**1e** (1.0)	KO*^t^*Bu	70	5	98	87/13
6	**1f** (1.0)	KO*^t^*Bu	70	12	94	85/15
7	**1g** (1.0)	KO*^t^*Bu	70	12	92	78/22
8^a^	**1e** (1.0)	KO*^t^*Bu	70	12	26	-b
9^c^	**1e** (1.0)	KO*^t^*Bu	70	12	5	-b

Reaction conditions: All reactions were conducted in 2 mL of toluene and conversion is based on ^1^H NMR spectroscopy.

a An open-air environment.

b Mixture of products.

c Reaction was conducted in water.

**Table 6:**
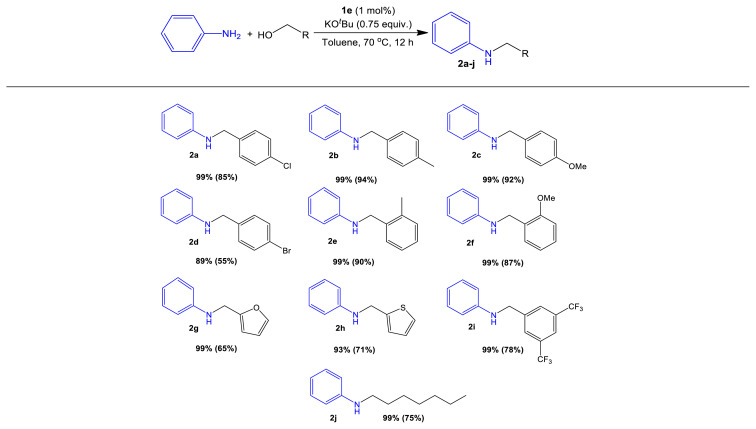
Aniline alkylation using a variety of primary alcohols.

**Table 7:**
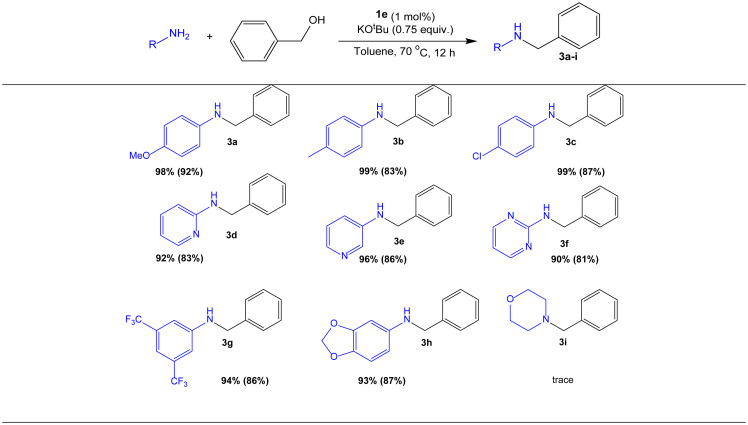
The use of benzyl alcohol to alkylate a variety of primary amines.

**Table 8:**
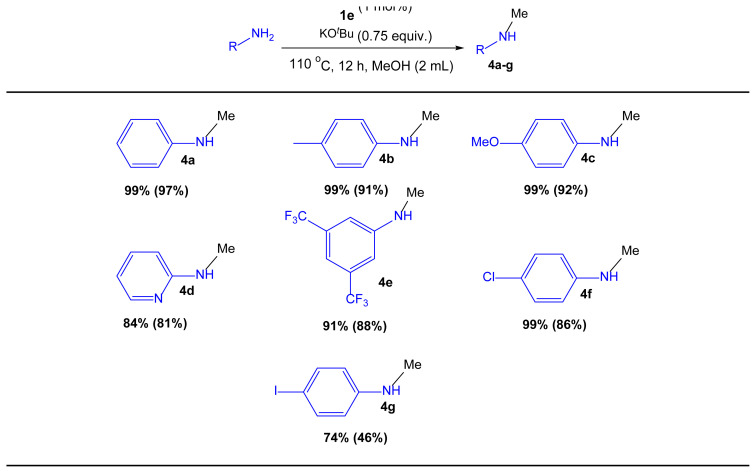
Methylation of aromatic amines.

**Table 9:** Comparison of Ru–NHC catalyst (**1e)** with reported NHC systems.

S/NO	Cat (mol %)	Substrate 1 Alcohol	Substrate 2 Primary amine	Temp (°C)	Time (h)	Yield (%)	Reference
1	1	aniline	Substituted alcohol	70	12	92	This work
2	2.5	-	-	120	24	97	[38]
3^a^	1.0	Substituted aniline	MeOH	150	24	84	[67]
4	0.5	-	-	130	24	85	[68]
5	1.0	-	-	135	36	95	[69]

a For the same reaction we use **1e** (1 mol %) at 110 **°C** for 12 h and we get 97% selective yield.

## Appendix A Supplementary data

CCDC 2085163 and 2173756 contain the supplementary crystallographic data for the compounds reported in this article. These data can be obtained free of charge on application to CCDC, 12 Union Road, Cambridge CB2 1EZ, UK.
