# Supplementary figures and images for: Benzimidazol-2-ylidene ruthenium complexes for C–N bond formation through alcohol dehydrogenation
Source: Turk J Chem. 2023 Sep 30;47(5):1209–23. doi: 10.55730/1300-0527.3606 (PMC10760900; doi:10.55730/1300-0527.3606)

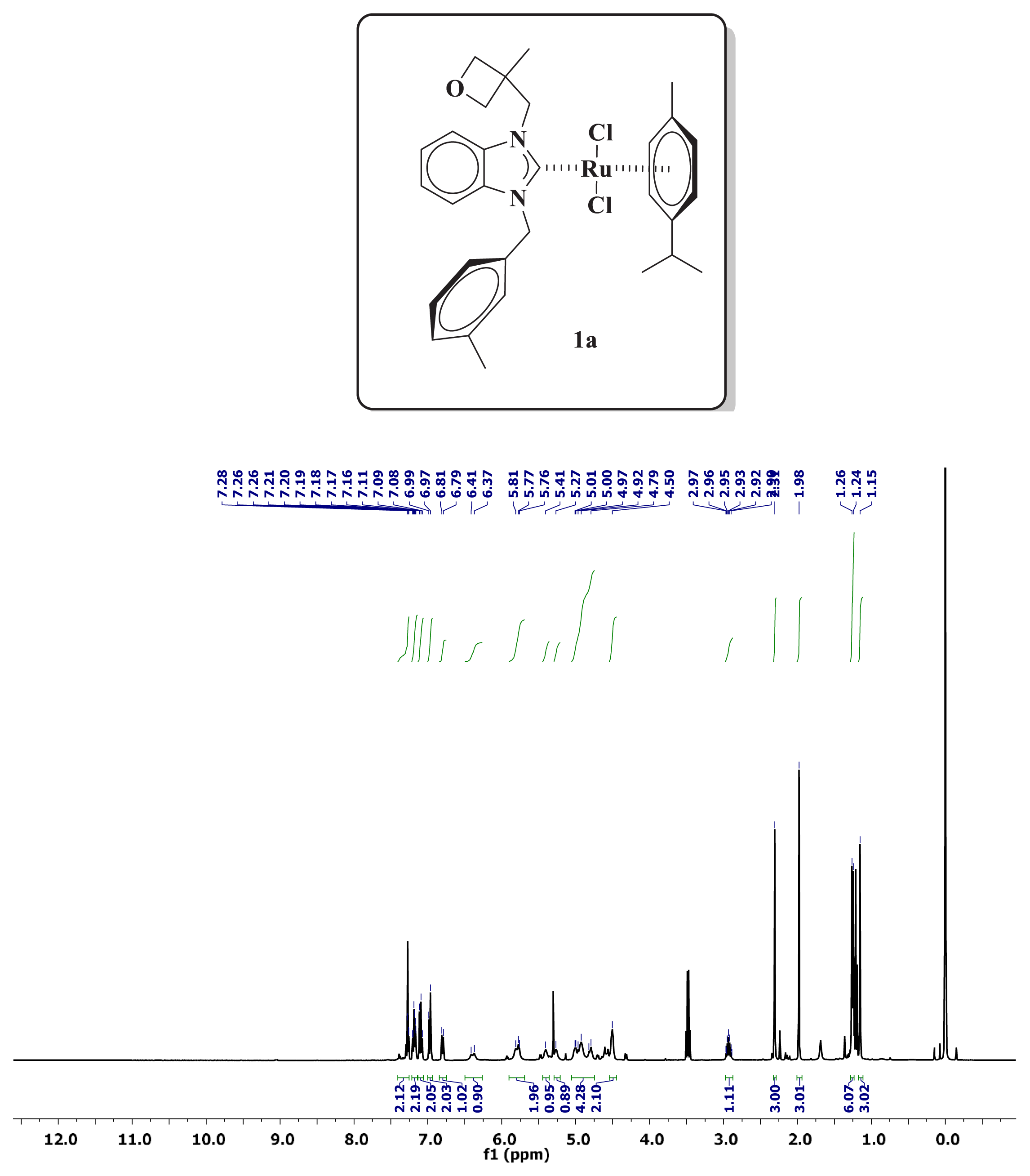

Supplement: Figure S1 — 1H NMR spectrum of ruthenium–BNHC complex 1a (in CDCl3, 25 °C, TMS, 400 MHz). [file turkjchem-47-5-1209s1.tif]

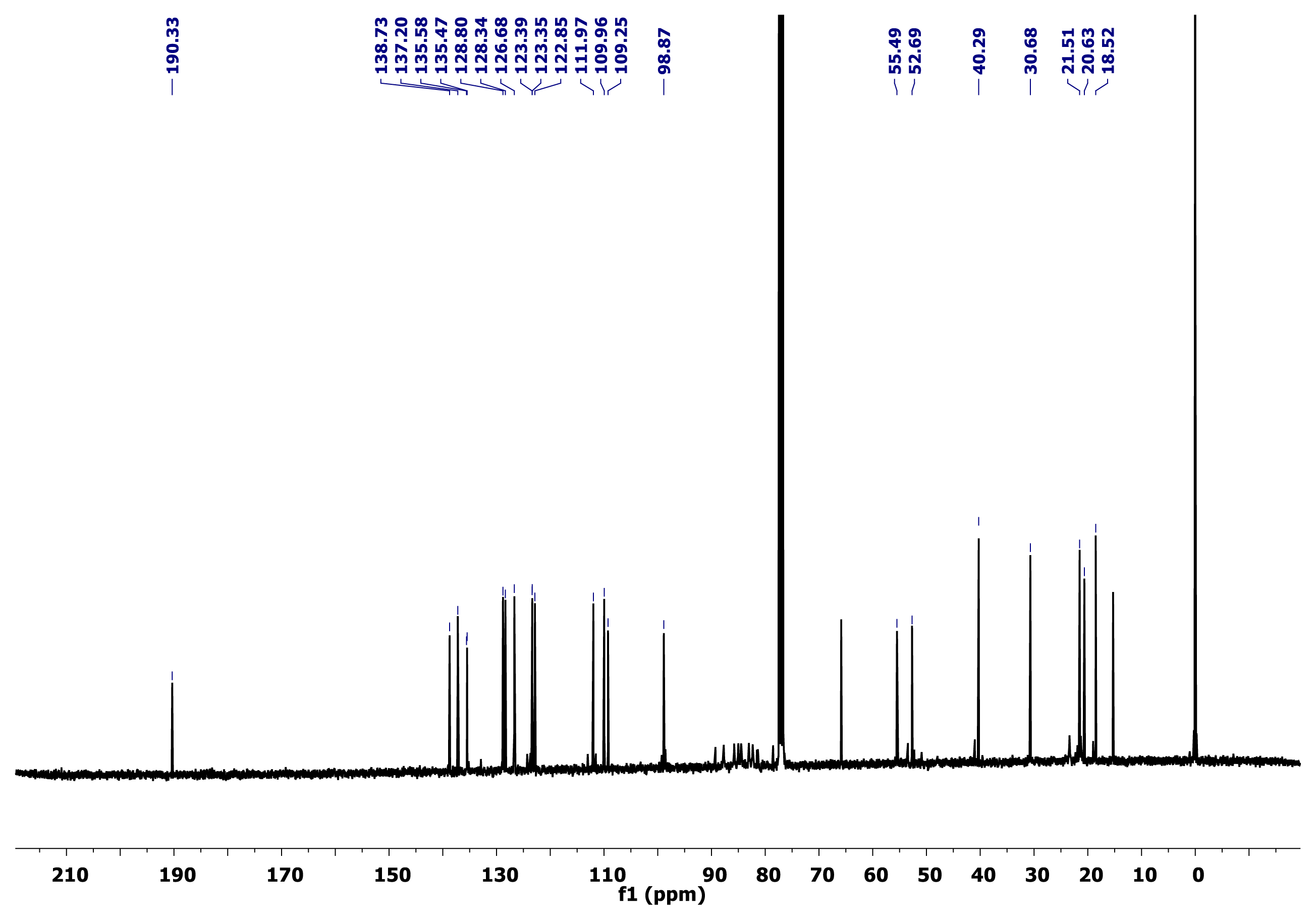

Supplement: Figure S2 — 13C NMR spectrum of ruthenium–BNHC complex 1a (in CDCl3, 25 °C, TMS, 101 MHz). [file turkjchem-47-5-1209s2.tif]

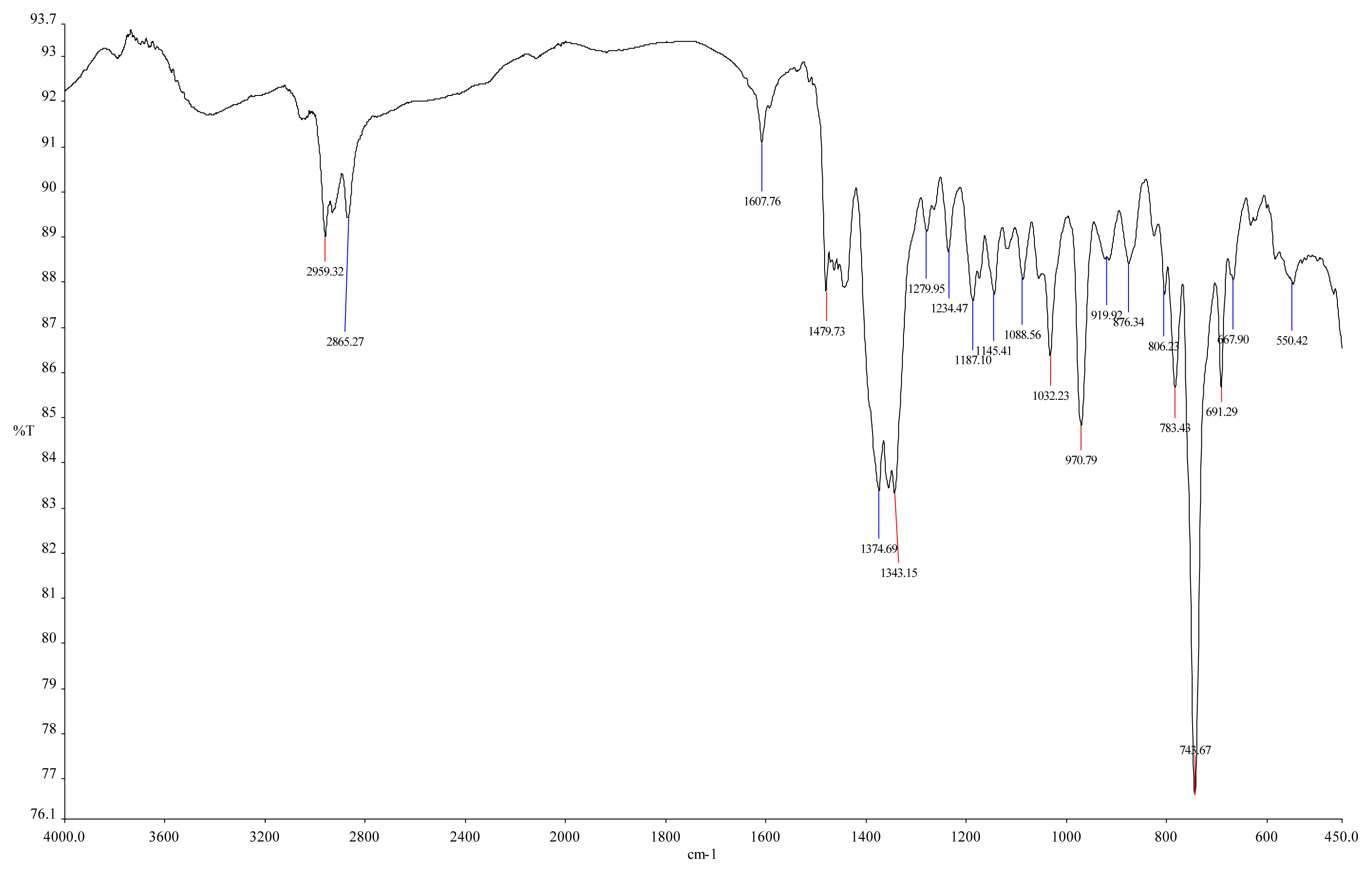

Supplement: Figure S3 — FT-IR spectrum of ruthenium–BNHC complex 1a. [file turkjchem-47-5-1209s3.tif]

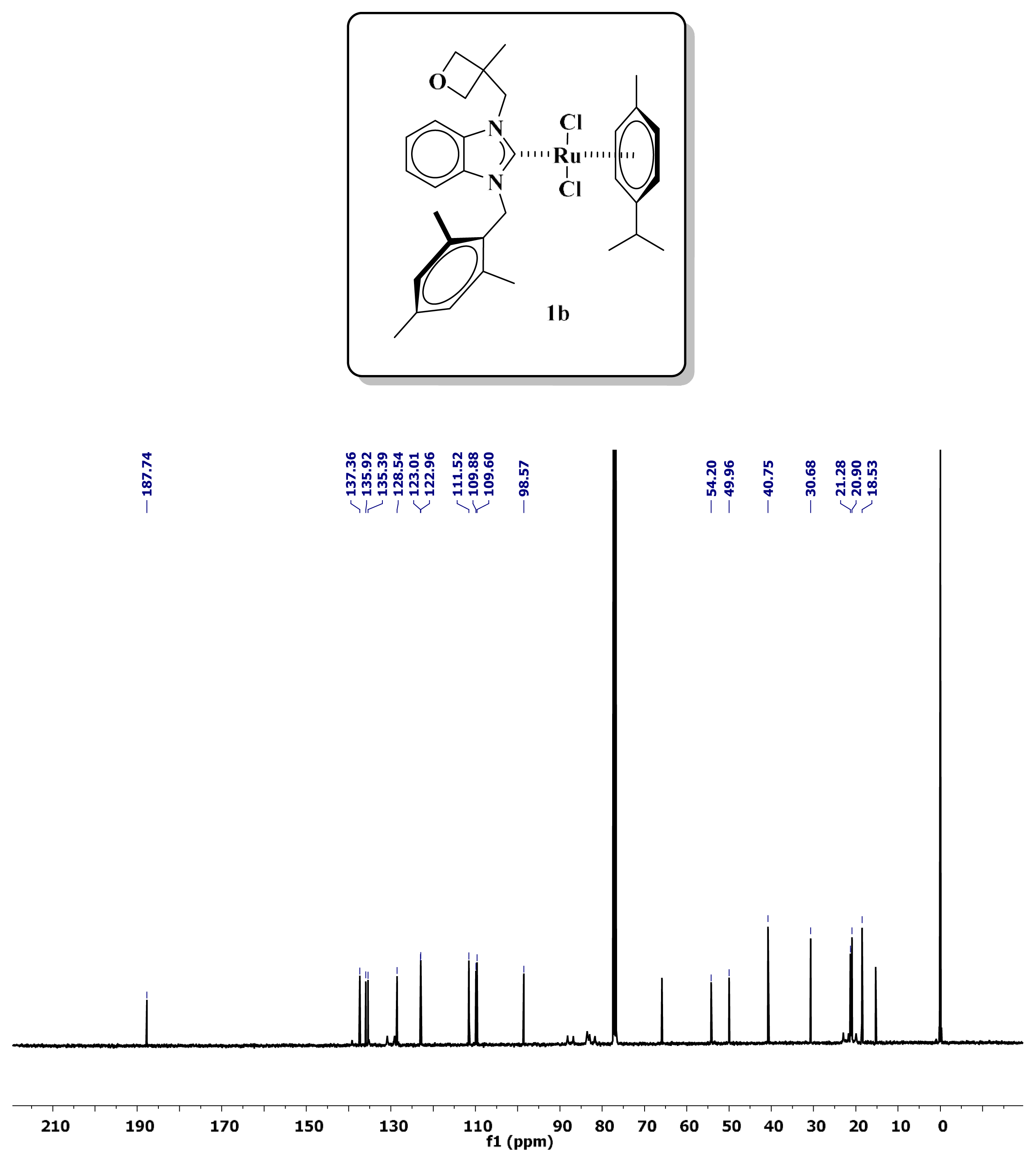

Supplement: Figure S4 — 1H NMR spectrum of ruthenium–BNHC complex 1b (in CDCl3, 25 °C, TMS, 400 MHz). [file turkjchem-47-5-1209s4.tif]

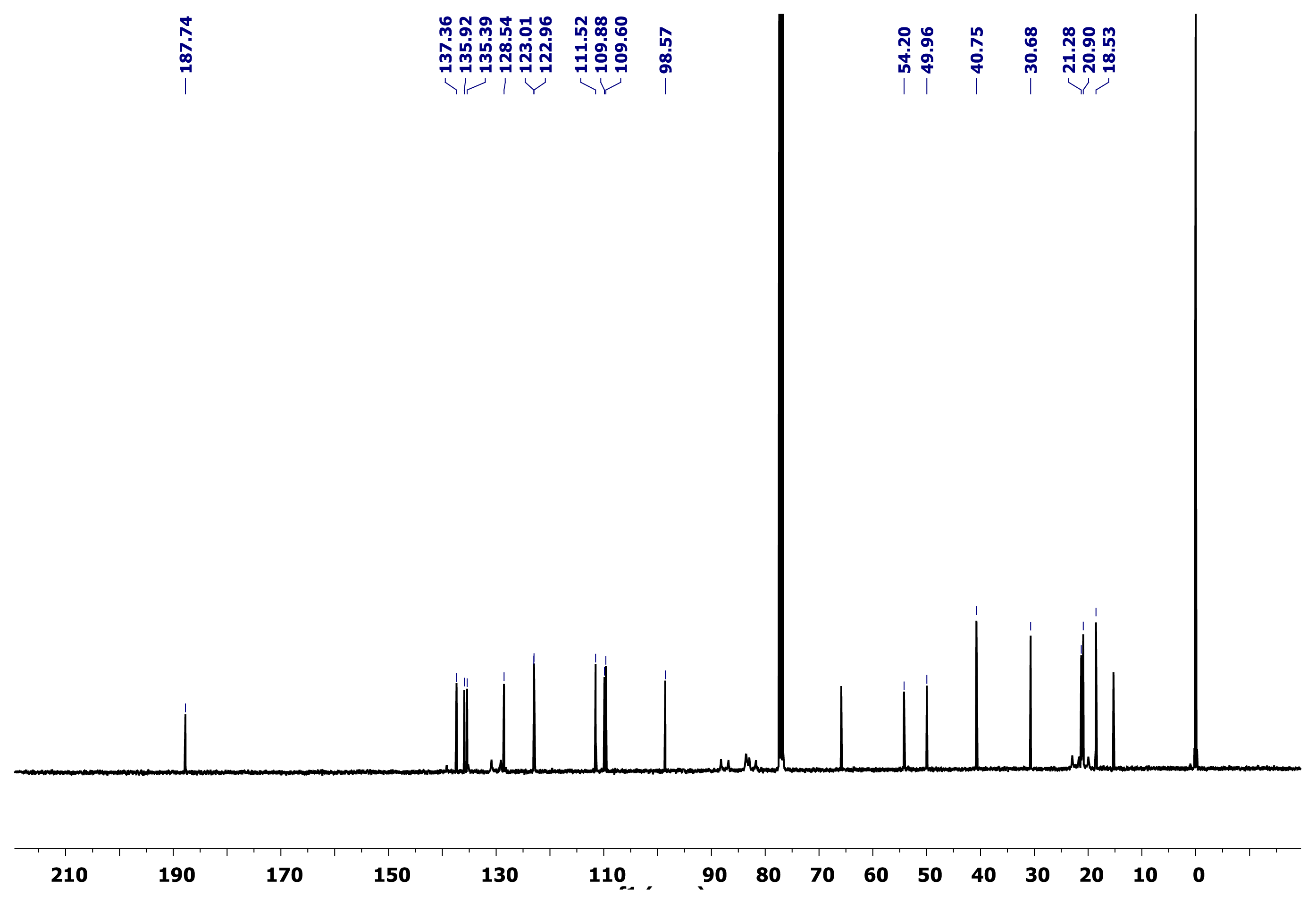

Supplement: Figure S5 — 13C NMR spectrum of ruthenium–BNHC complex 1b (in CDCl3, 25 °C, TMS, 101 MHz). [file turkjchem-47-5-1209s5.tif]

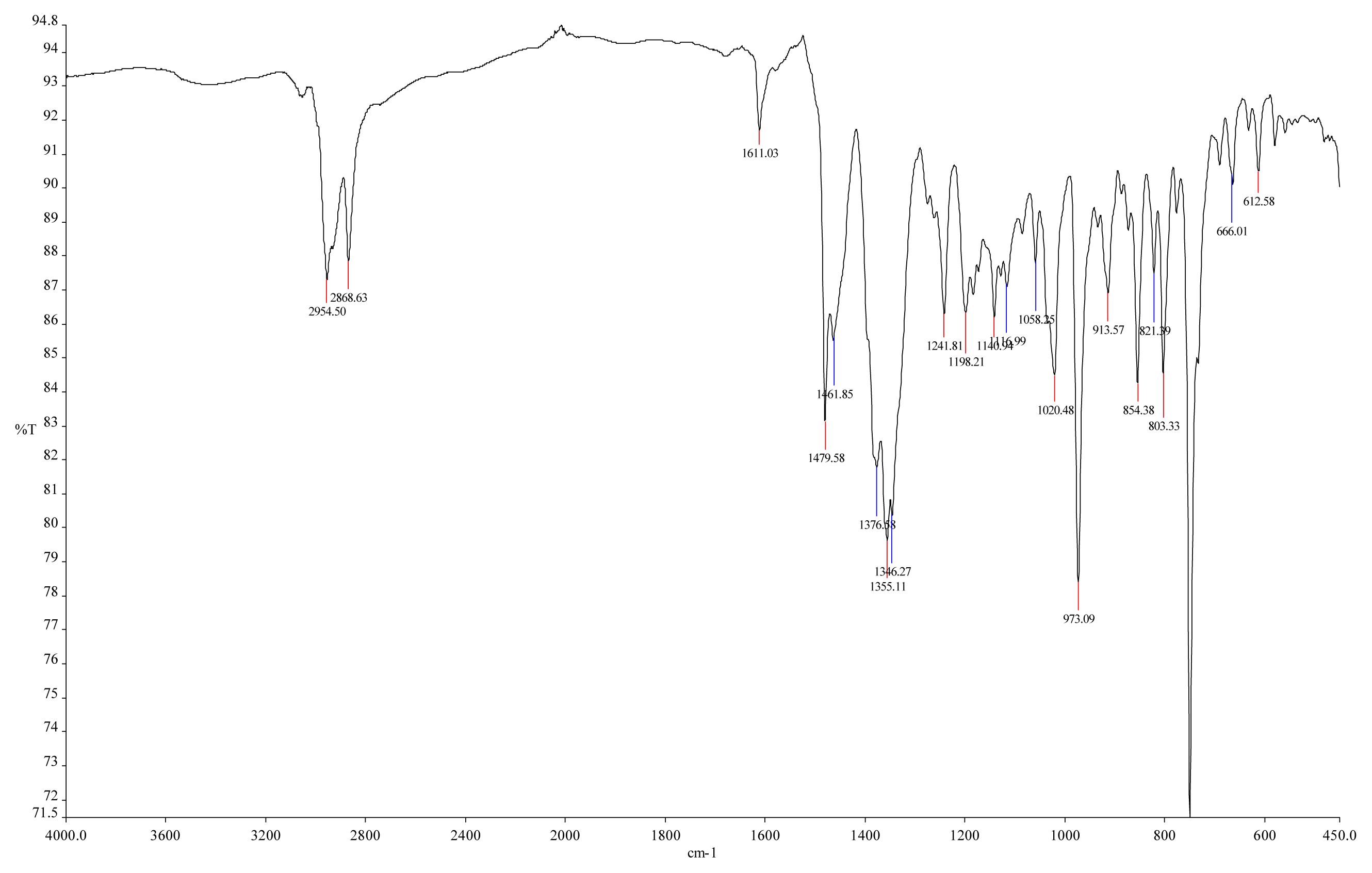

Supplement: Figure S6 — FT-IR spectrum of ruthenium–BNHC complex 1b. [file turkjchem-47-5-1209s6.tif]

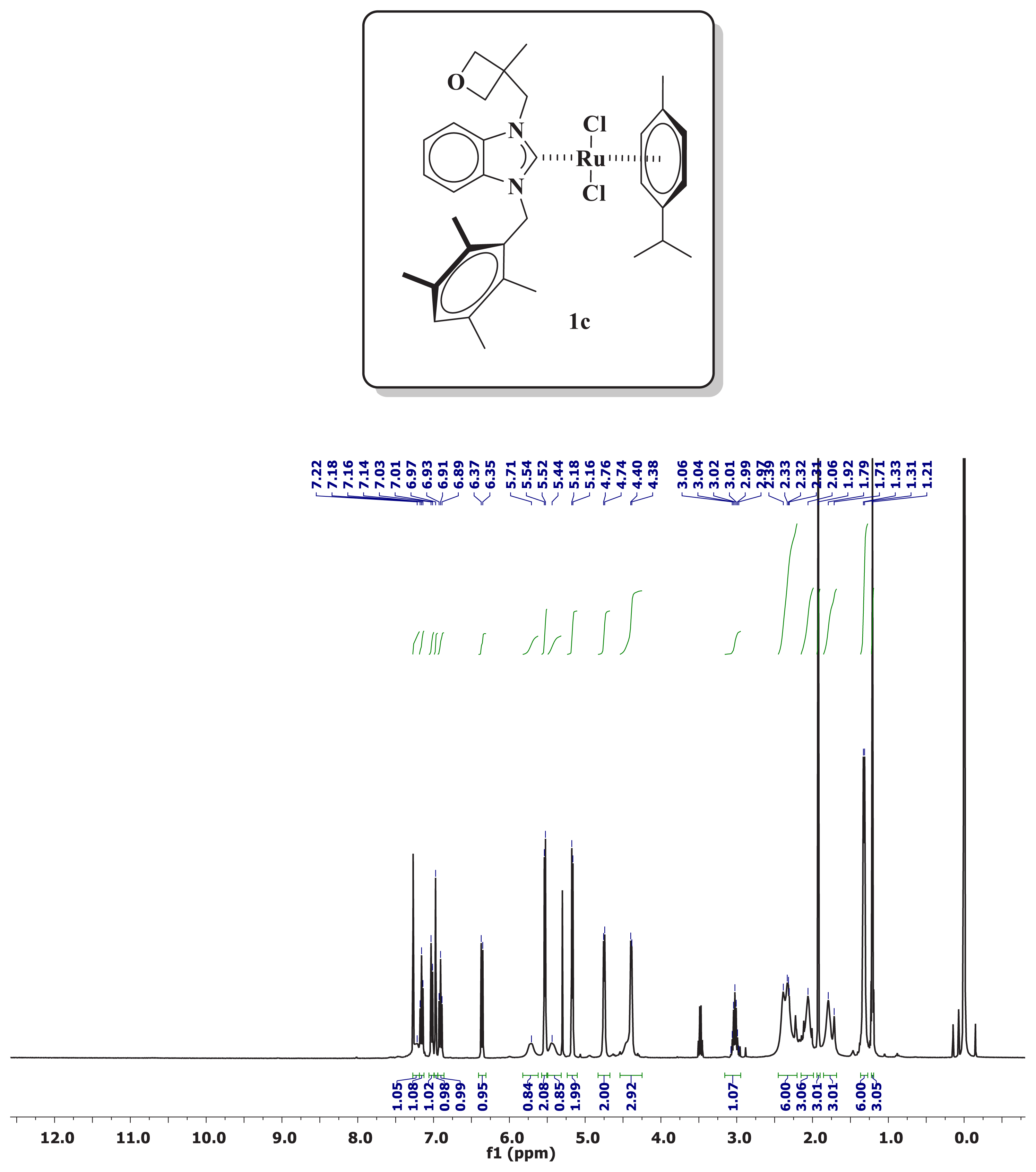

Supplement: Figure S7 — 1H NMR spectrum of ruthenium–BNHC complex 1c (in CDCl3, 25 °C, TMS, 400 MHz). [file turkjchem-47-5-1209s7.tif]

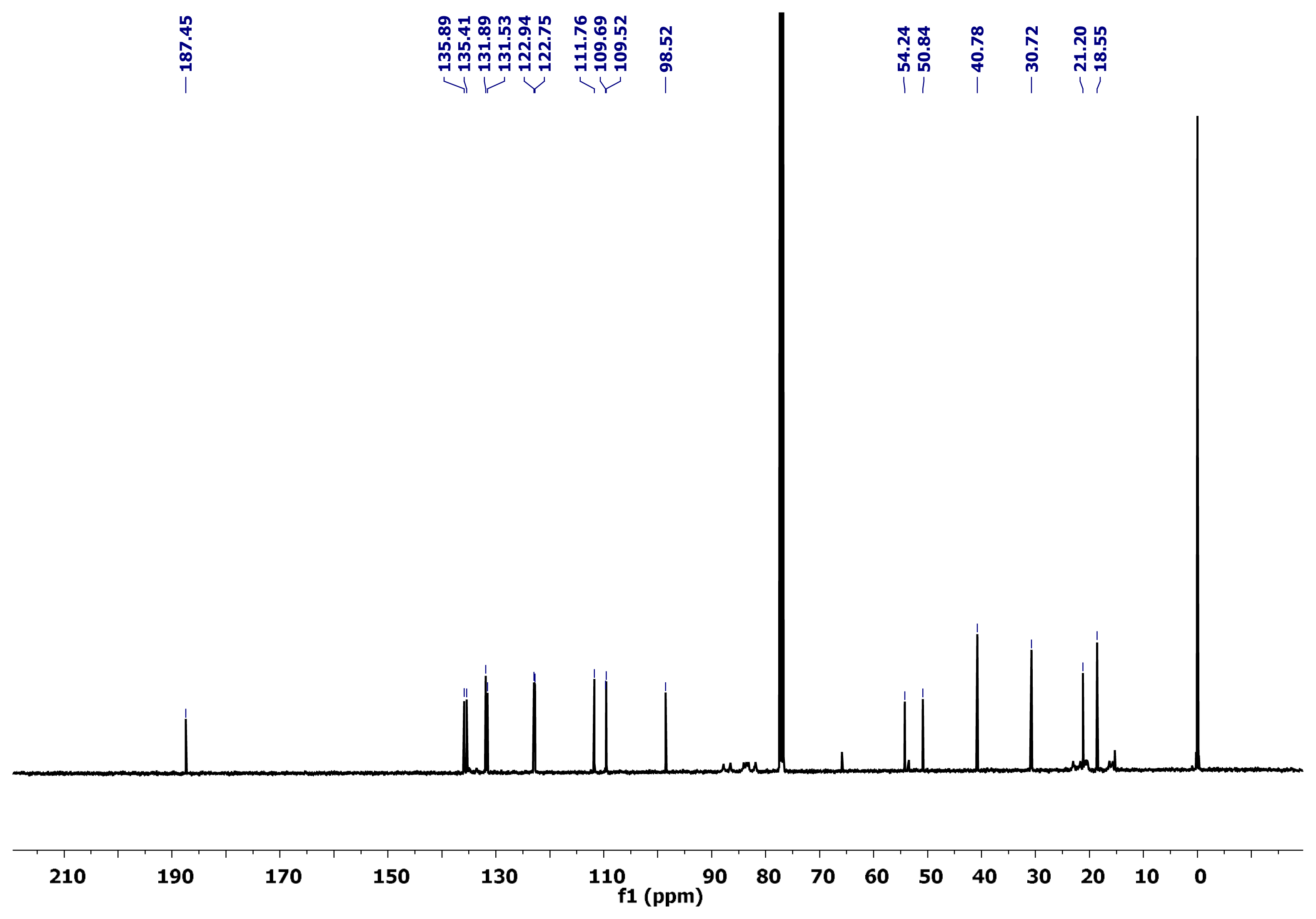

Supplement: Figure S8 — 13C NMR spectrum of ruthenium–BNHC complex 1c (in CDCl3, 25 °C, TMS, 101 MHz). [file turkjchem-47-5-1209s8.tif]

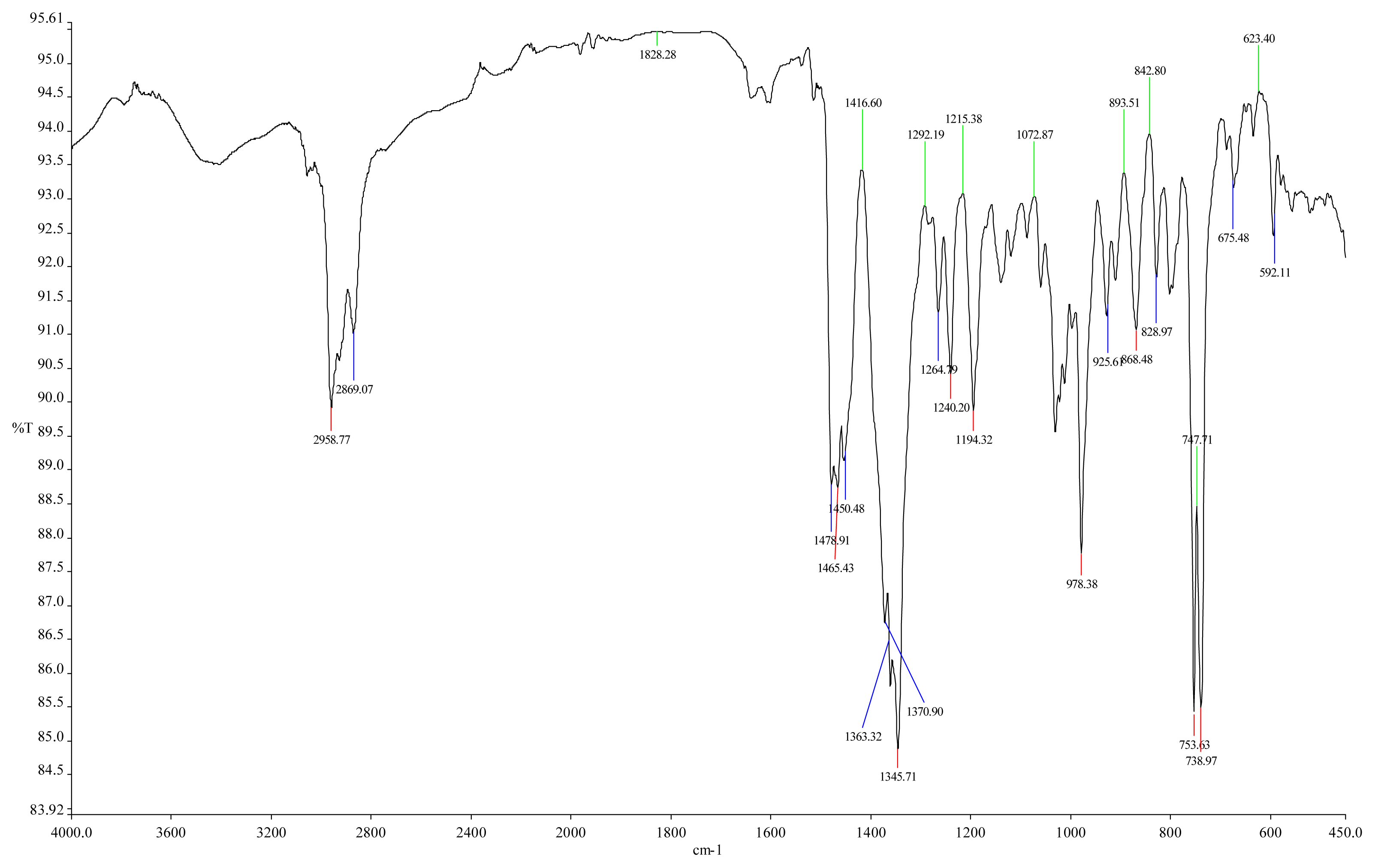

Supplement: Figure S9 — FT-IR spectrum of ruthenium–BNHC complex 1c. [file turkjchem-47-5-1209s9.tif]

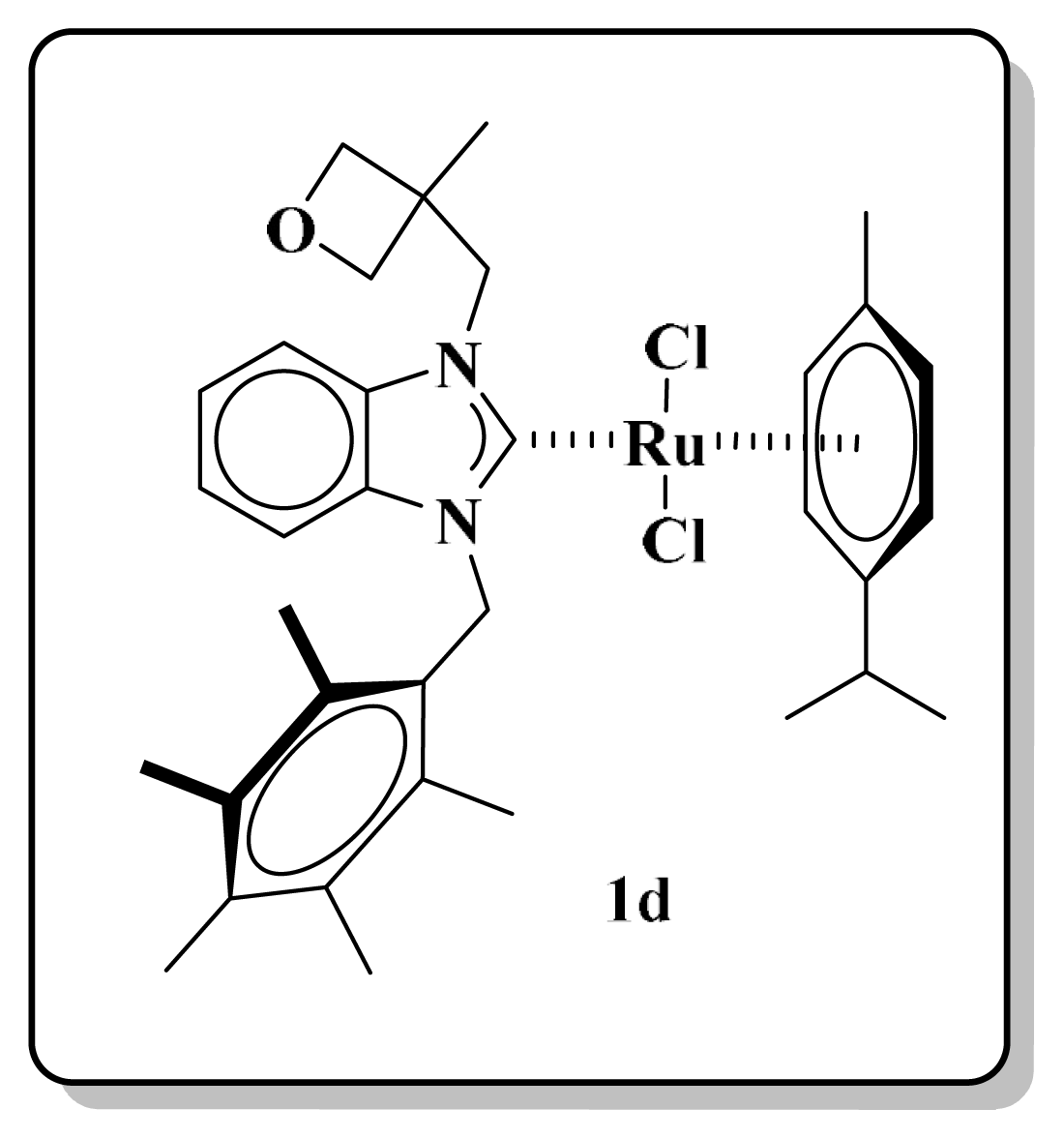

Supplement: Figure S10 — 1H NMR spectrum of ruthenium–BNHC complex 1d (in CDCl3, 25 °C, TMS, 400 MHz). [file turkjchem-47-5-1209s10a.tif]

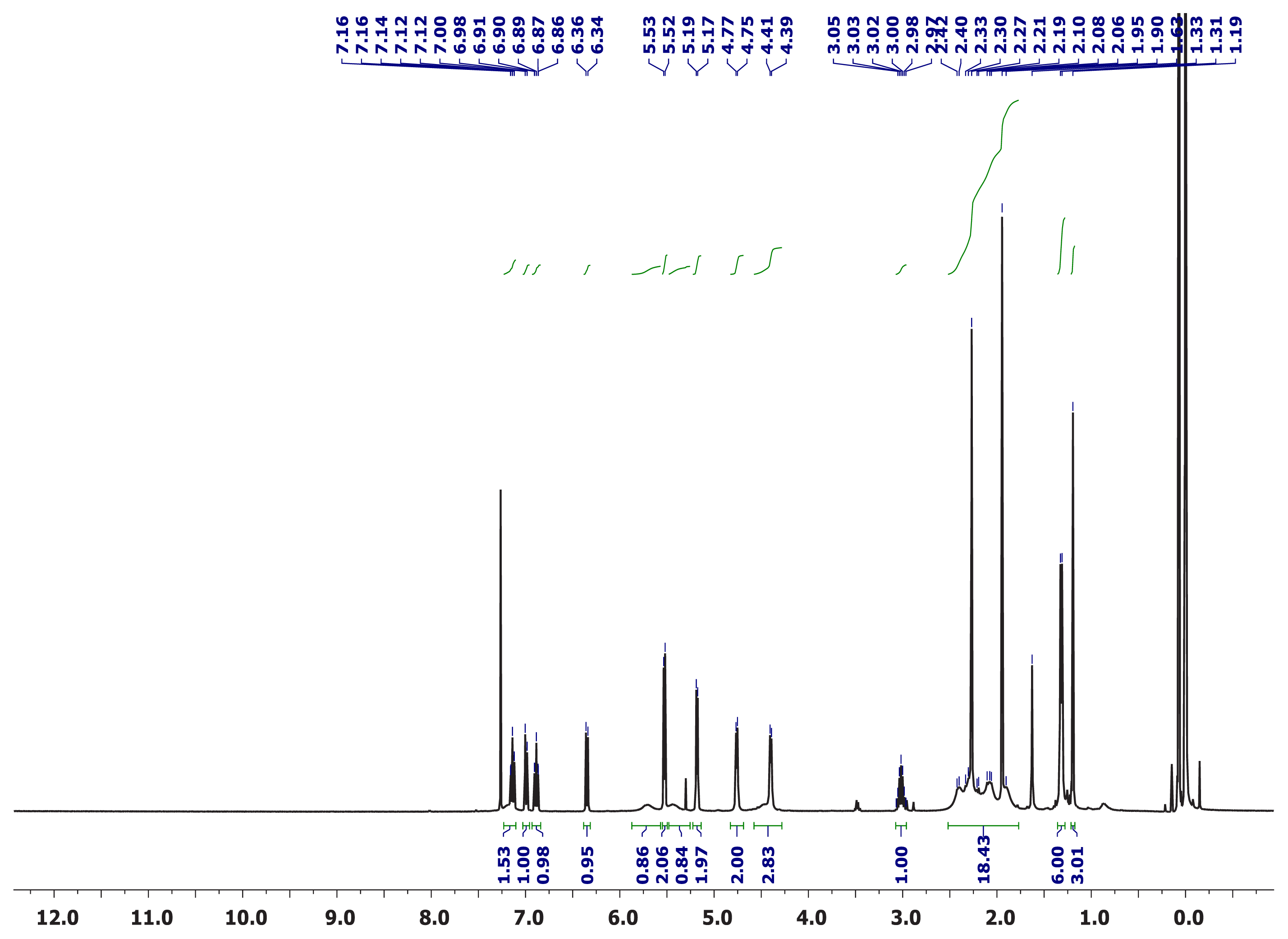

Supplement: Figure S10 — 1H NMR spectrum of ruthenium–BNHC complex 1d (in CDCl3, 25 °C, TMS, 400 MHz). [file turkjchem-47-5-1209s10b.tif]

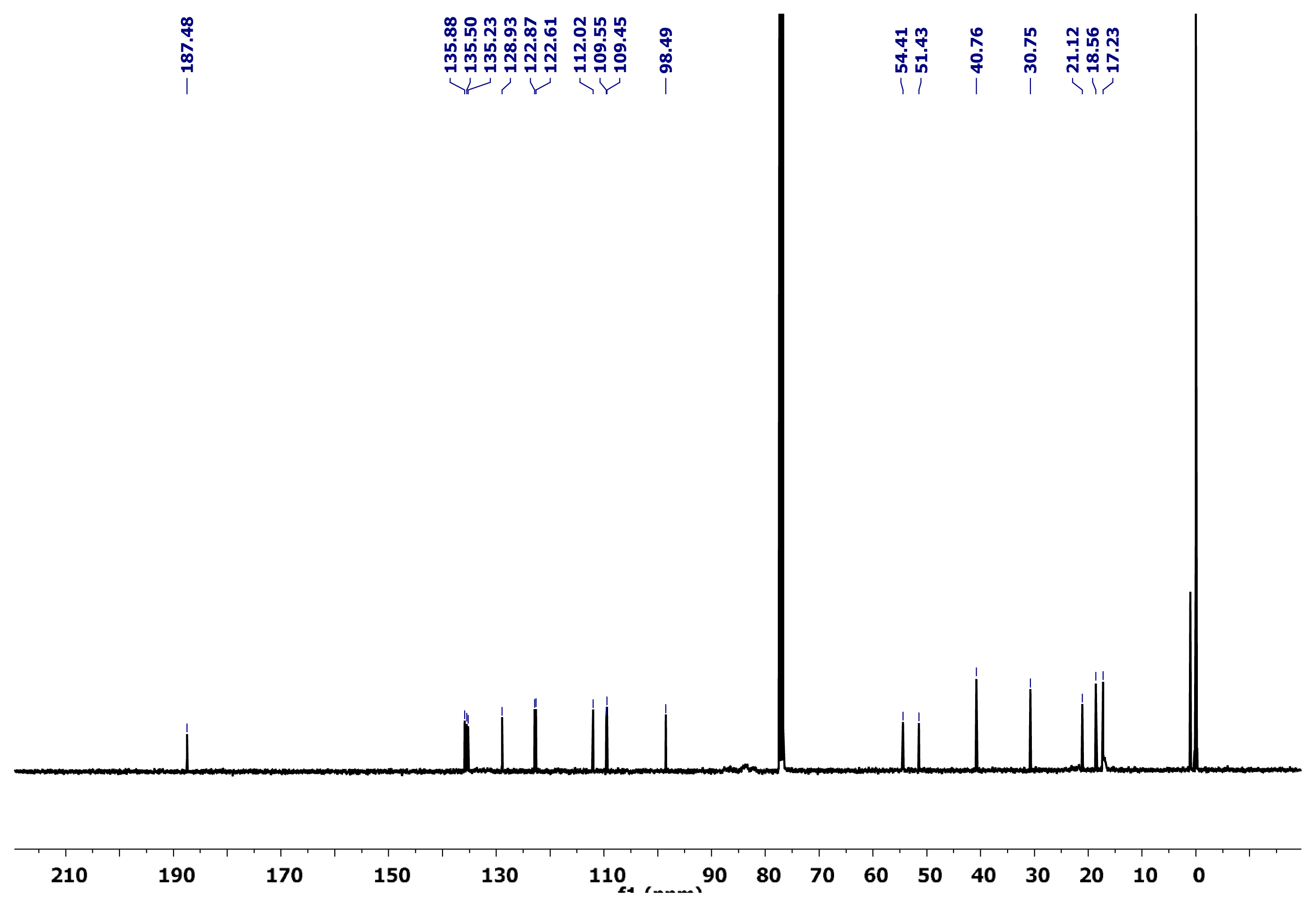

Supplement: Figure S11 — 13C NMR spectrum of ruthenium–BNHC complex 1d (in CDCl3, 25 °C, TMS, 101 MHz). [file turkjchem-47-5-1209s11.tif]

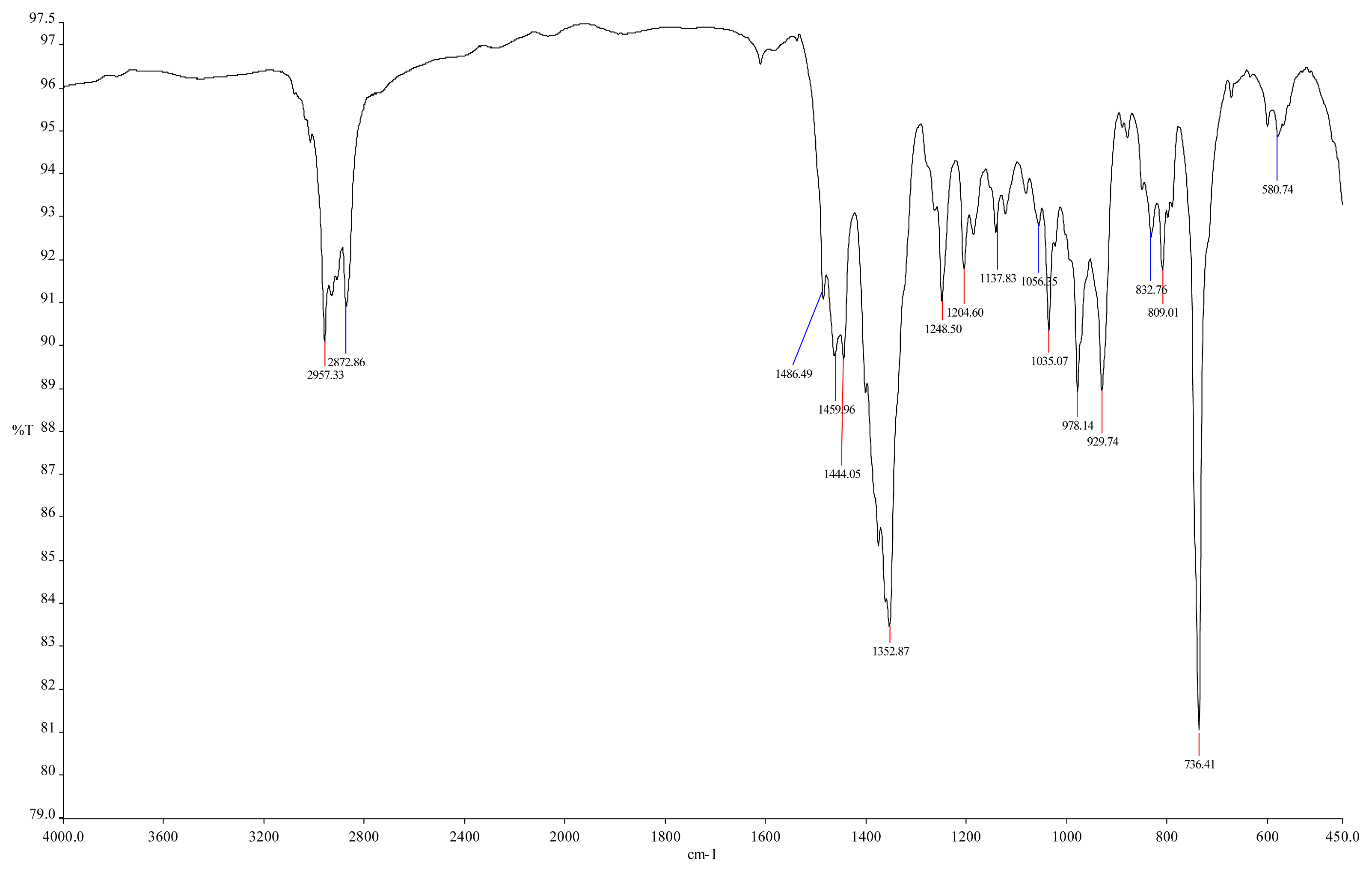

Supplement: Figure S12 — FT-IR spectrum of ruthenium–BNHC complex 1d. [file turkjchem-47-5-1209s12.tif]

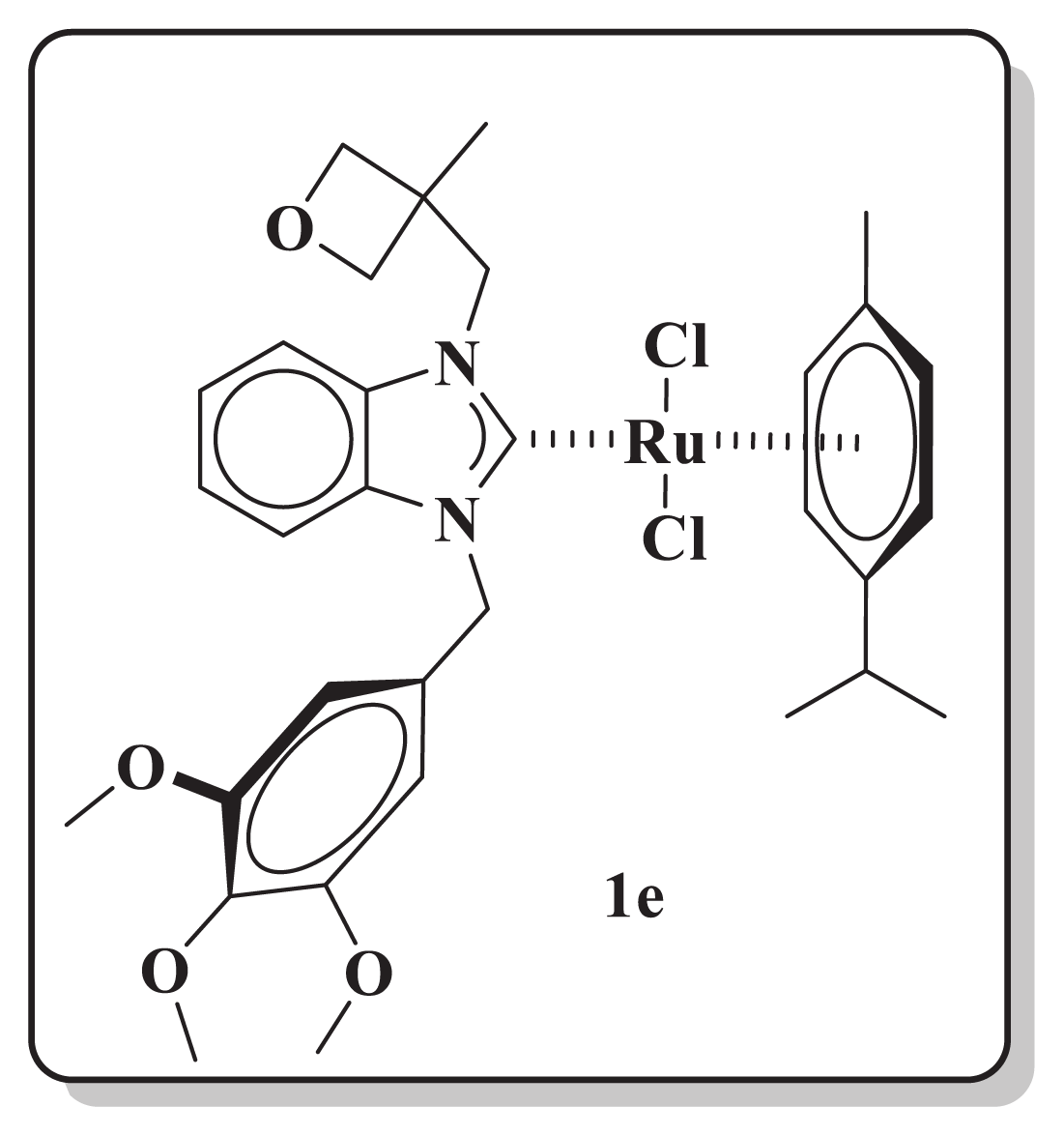

Supplement: Figure S13 — 1H NMR spectrum of ruthenium–BNHC complex 1e (in CDCl3, 25 °C, TMS, 400 MHz). [file turkjchem-47-5-1209s13a.tif]

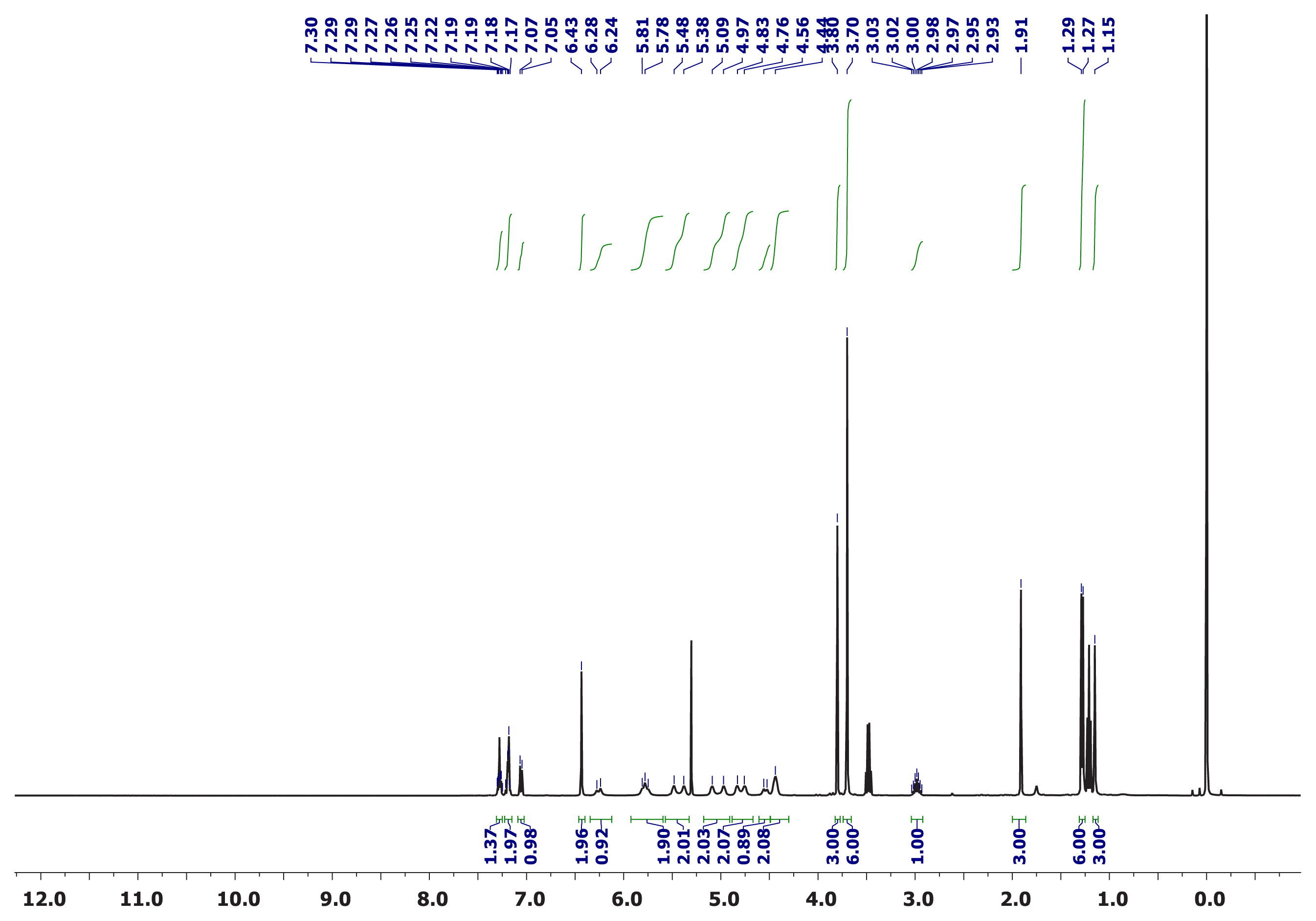

Supplement: Figure S13 — 1H NMR spectrum of ruthenium–BNHC complex 1e (in CDCl3, 25 °C, TMS, 400 MHz). [file turkjchem-47-5-1209s13b.tif]

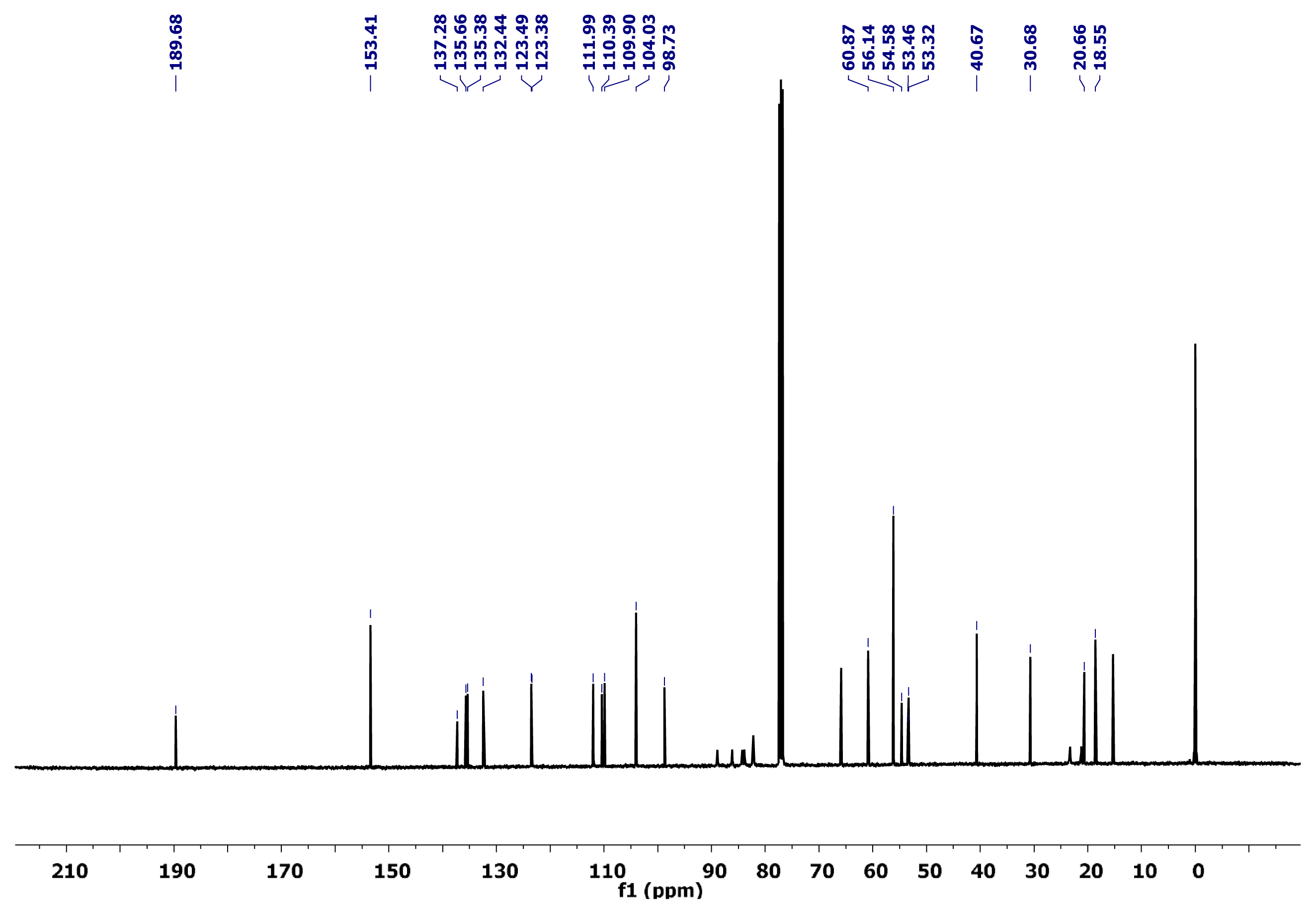

Supplement: Figure S14 — 13C NMR spectrum of ruthenium–BNHC complex 1e (in CDCl3, 25 °C, TMS, 101 MHz). [file turkjchem-47-5-1209s14.tif]

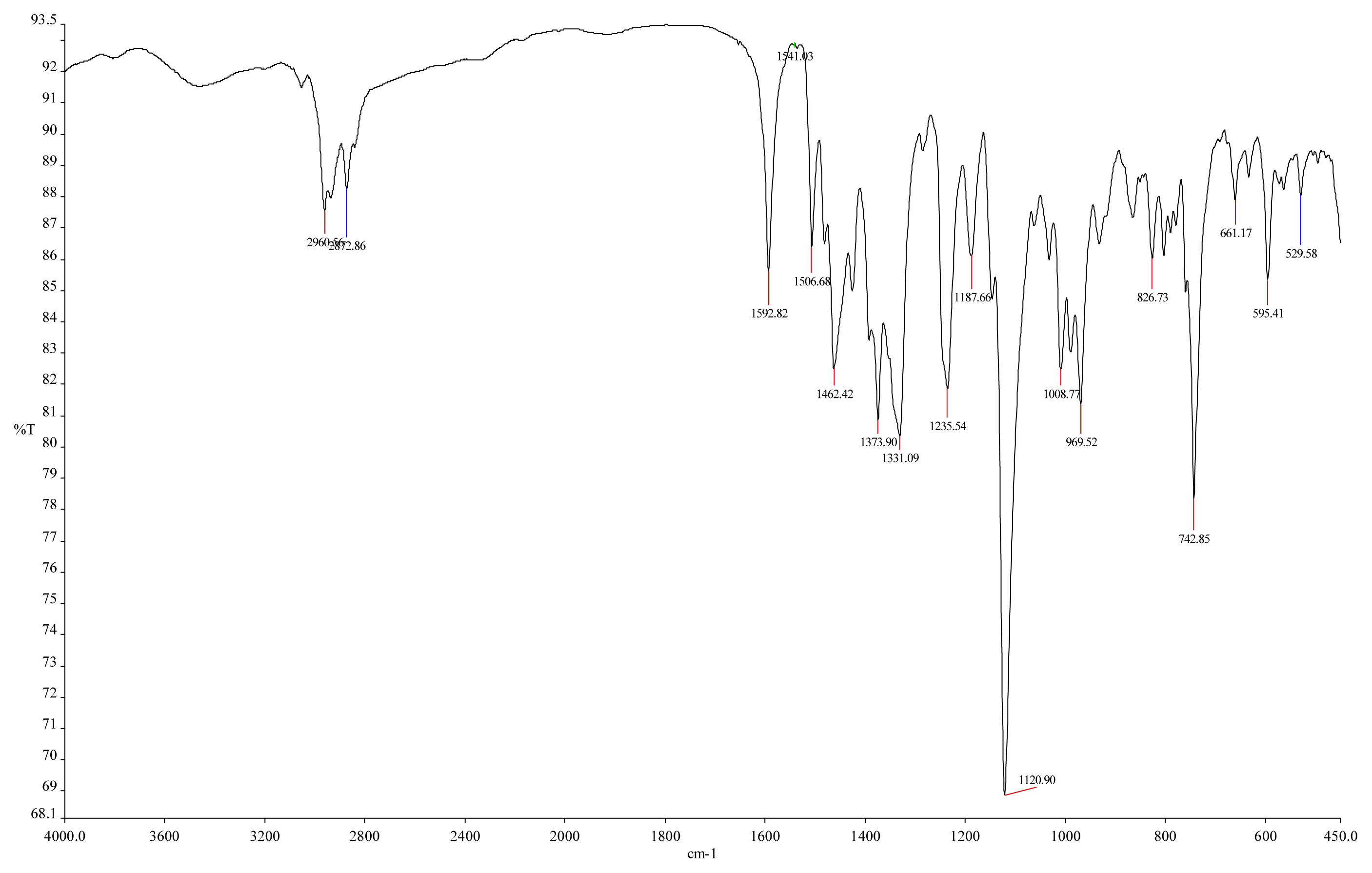

Supplement: Figure S15 — FT-IR spectrum of ruthenium–BNHC complex 1e. [file turkjchem-47-5-1209s15.tif]

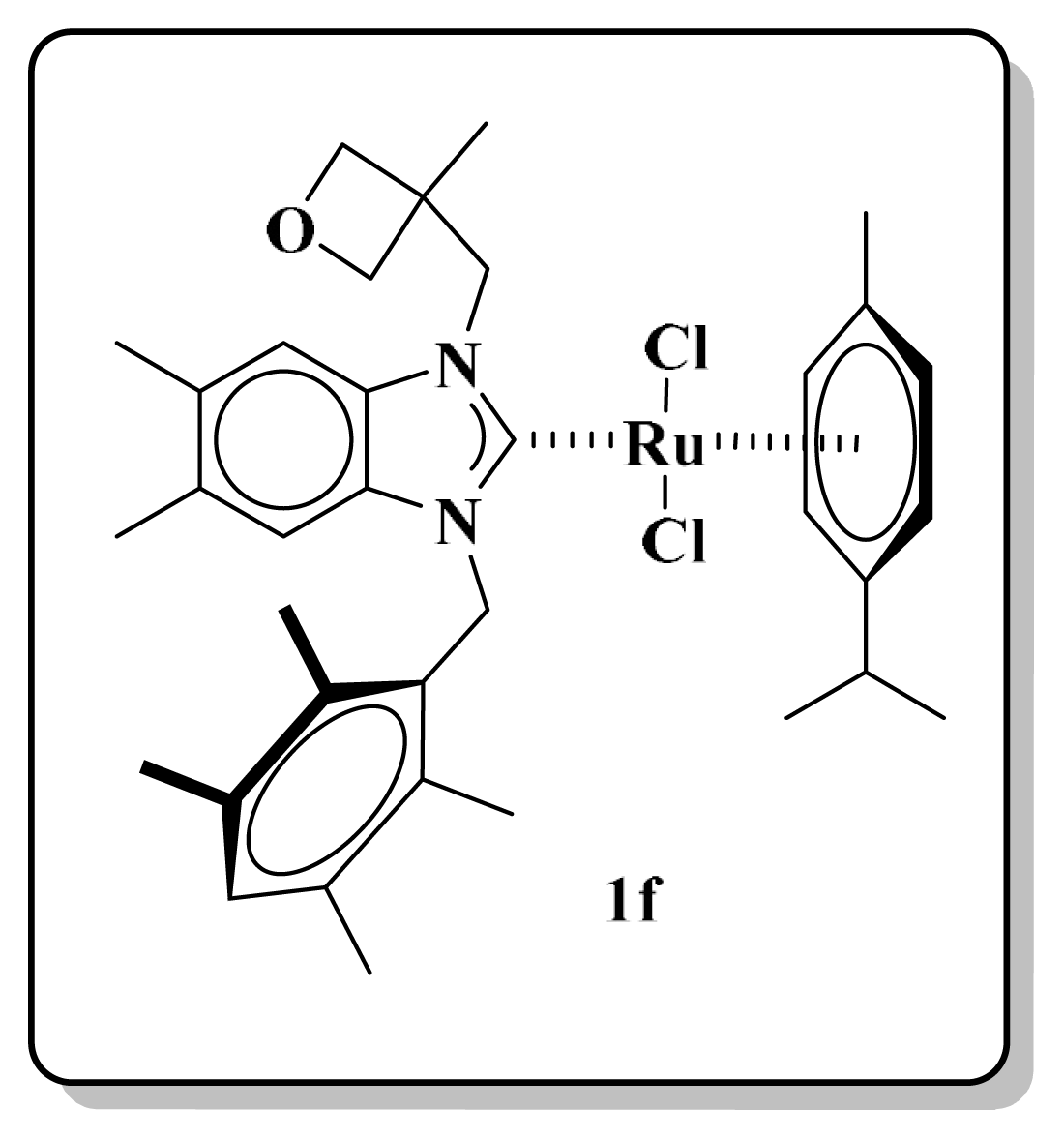

Supplement: Figure S16 — 1H NMR spectrum of ruthenium–BNHC complex 1f (in CDCl3, 25 °C, TMS, 400 MHz). [file turkjchem-47-5-1209s16a.tif]

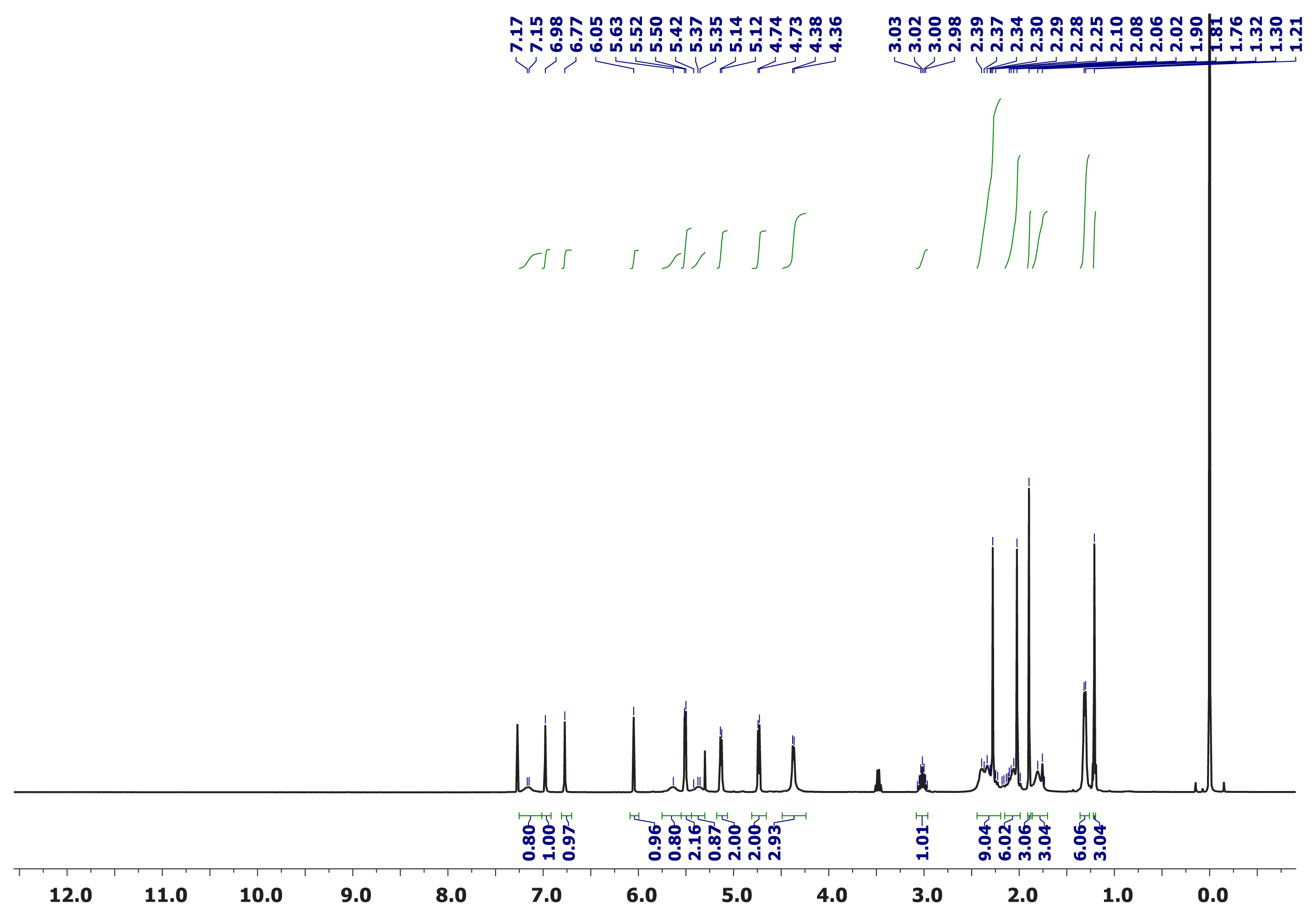

Supplement: Figure S16 — 1H NMR spectrum of ruthenium–BNHC complex 1f (in CDCl3, 25 °C, TMS, 400 MHz). [file turkjchem-47-5-1209s16b.tif]

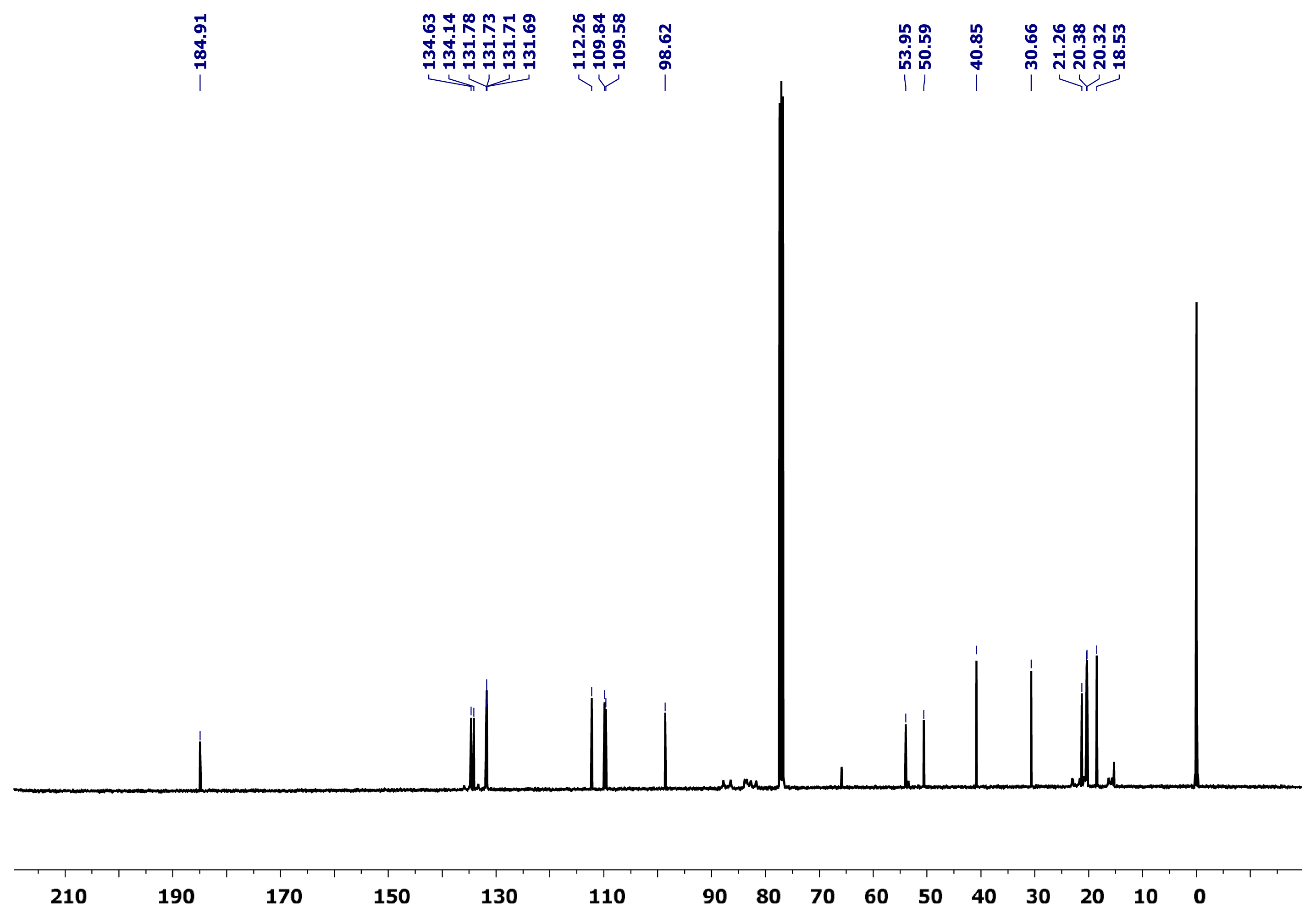

Supplement: Figure S17 — 13C NMR spectrum of ruthenium–BNHC complex 1f (in CDCl3, 25 °C, TMS, 101 MHz). [file turkjchem-47-5-1209s17.tif]

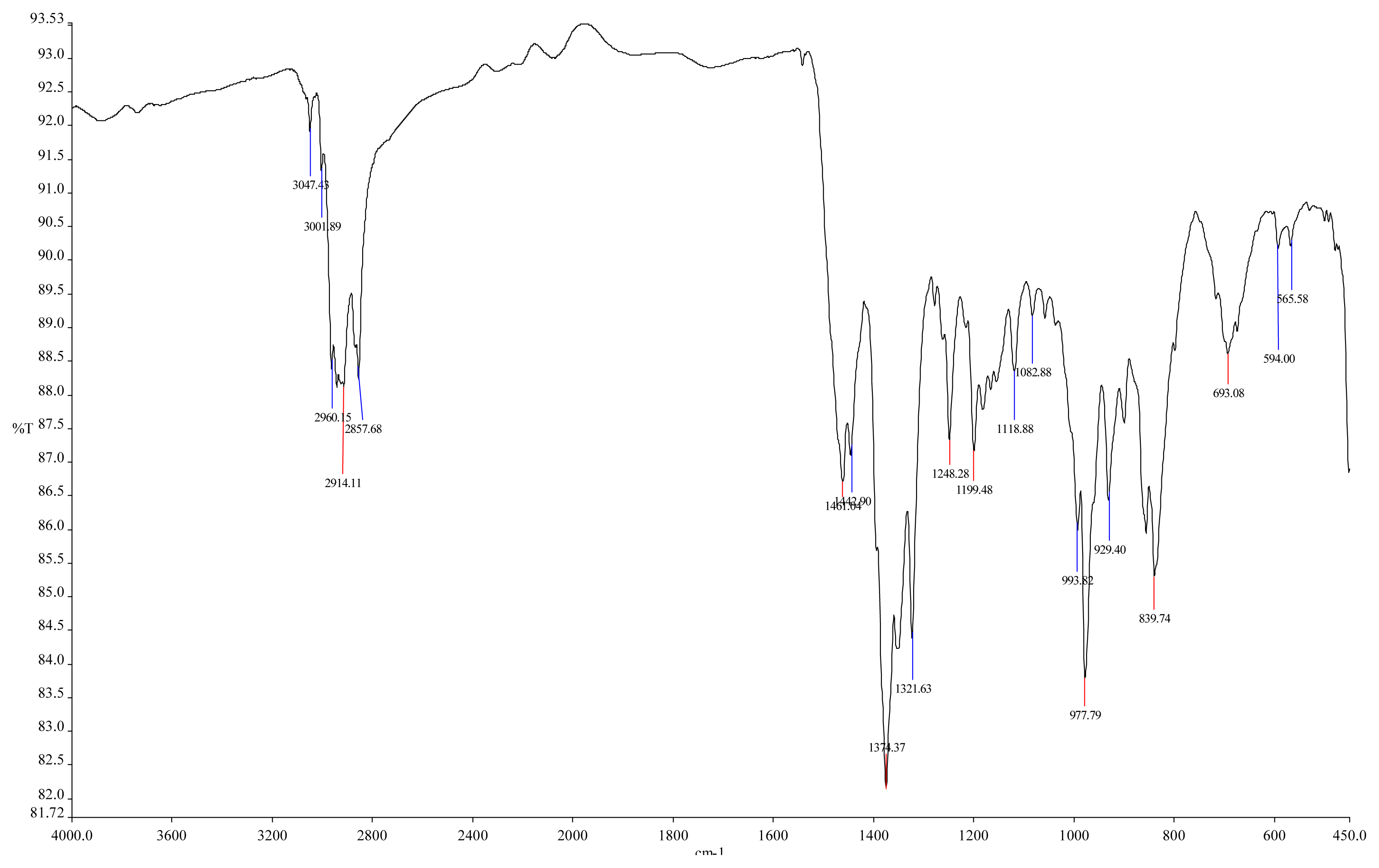

Supplement: Figure S18 — FT-IR spectrum of ruthenium–BNHC complex 1f. [file turkjchem-47-5-1209s18.tif]

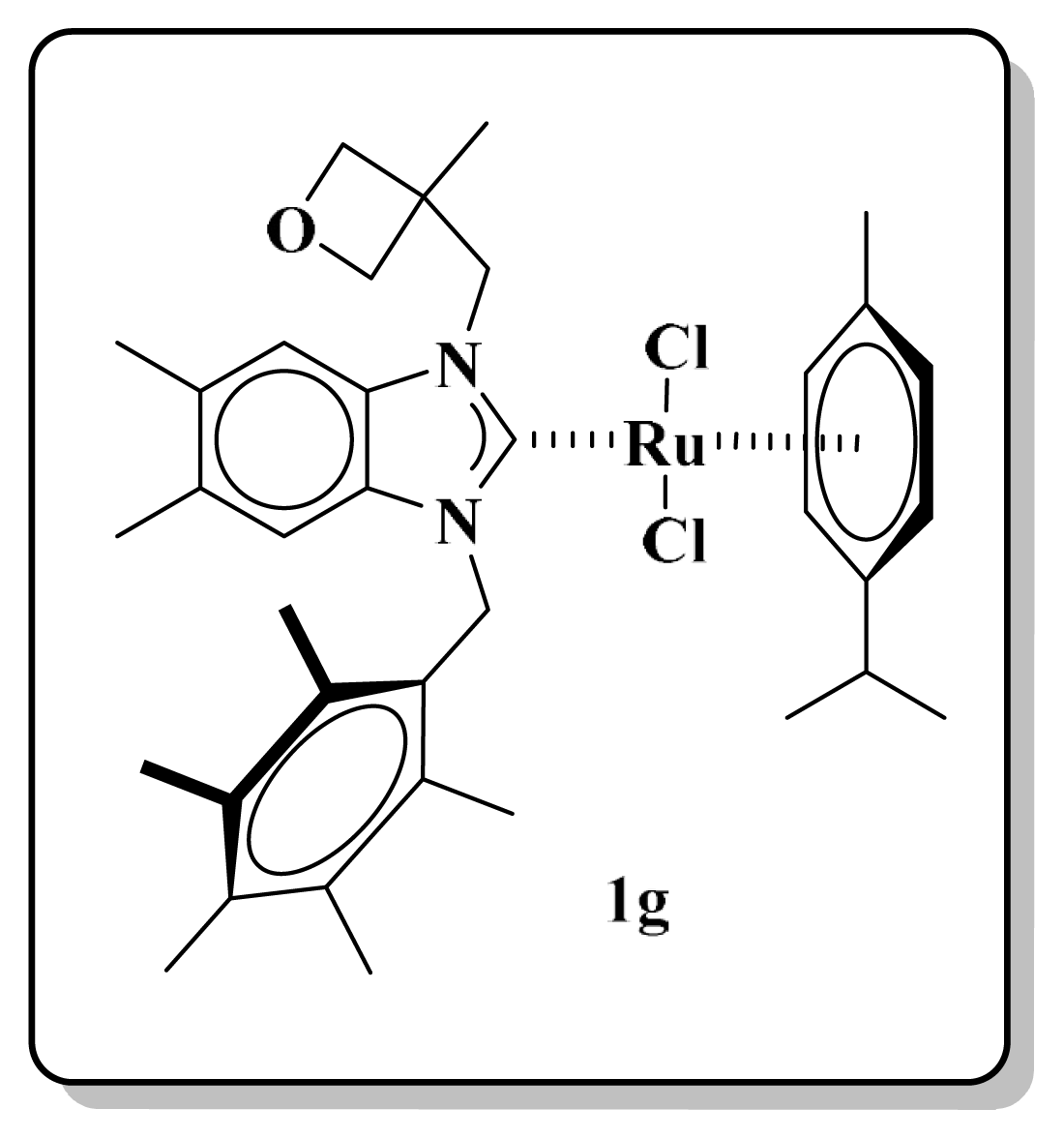

Supplement: Figure S19 — 1H NMR spectrum of ruthenium–BNHC complex 1g (in CDCl3, 25 °C, TMS, 400 MHz). [file turkjchem-47-5-1209s19a.tif]

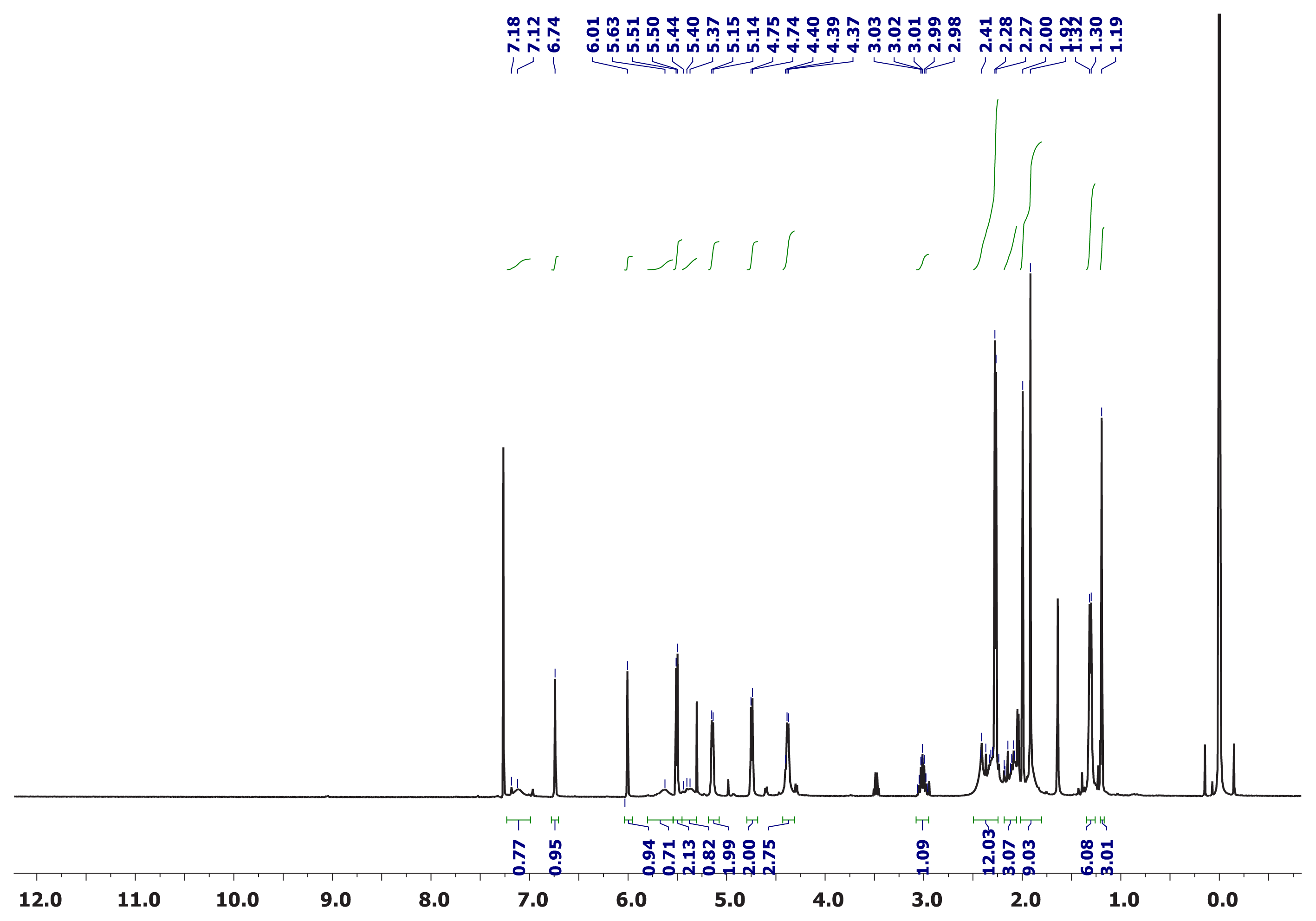

Supplement: Figure S19 — 1H NMR spectrum of ruthenium–BNHC complex 1g (in CDCl3, 25 °C, TMS, 400 MHz). [file turkjchem-47-5-1209s19b.tif]

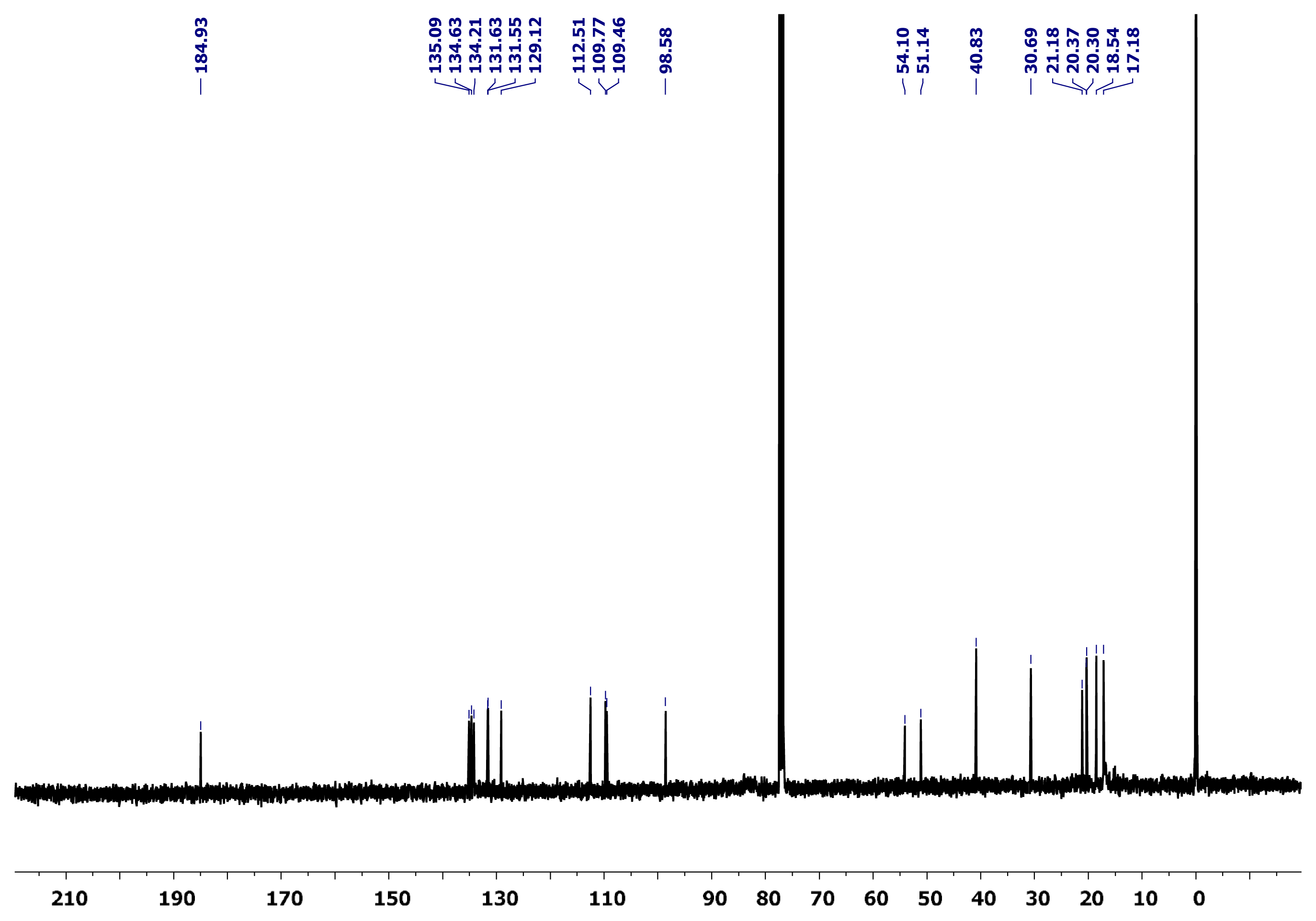

Supplement: Figure S20 — 13C NMR spectrum of ruthenium–BNHC complex 1g (in CDCl3, 25 °C, TMS, 101 MHz). [file turkjchem-47-5-1209s20.tif]

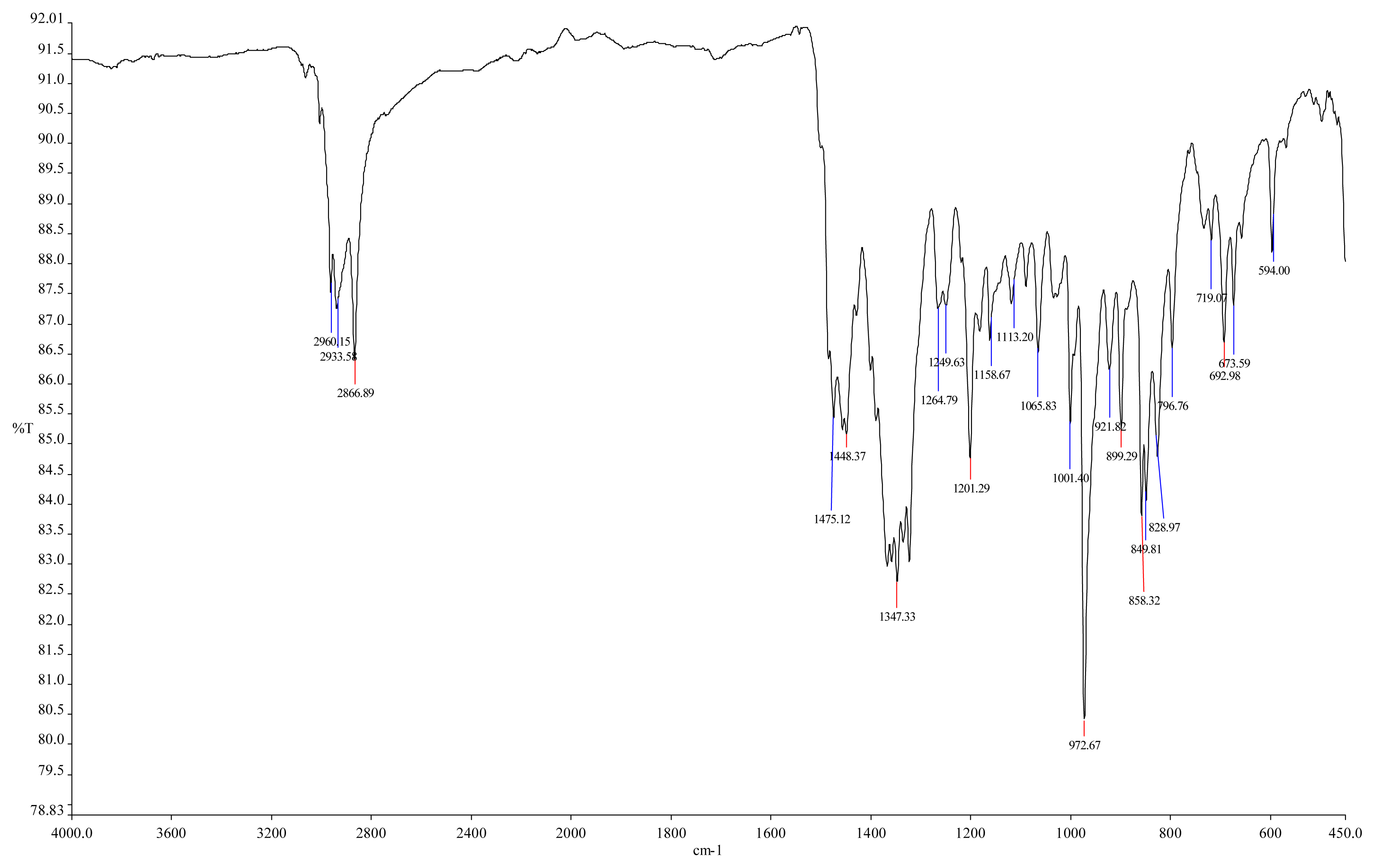

Supplement: Figure S21 — FT-IR spectrum of ruthenium–BNHC complex 1g. [file turkjchem-47-5-1209s21.tif]

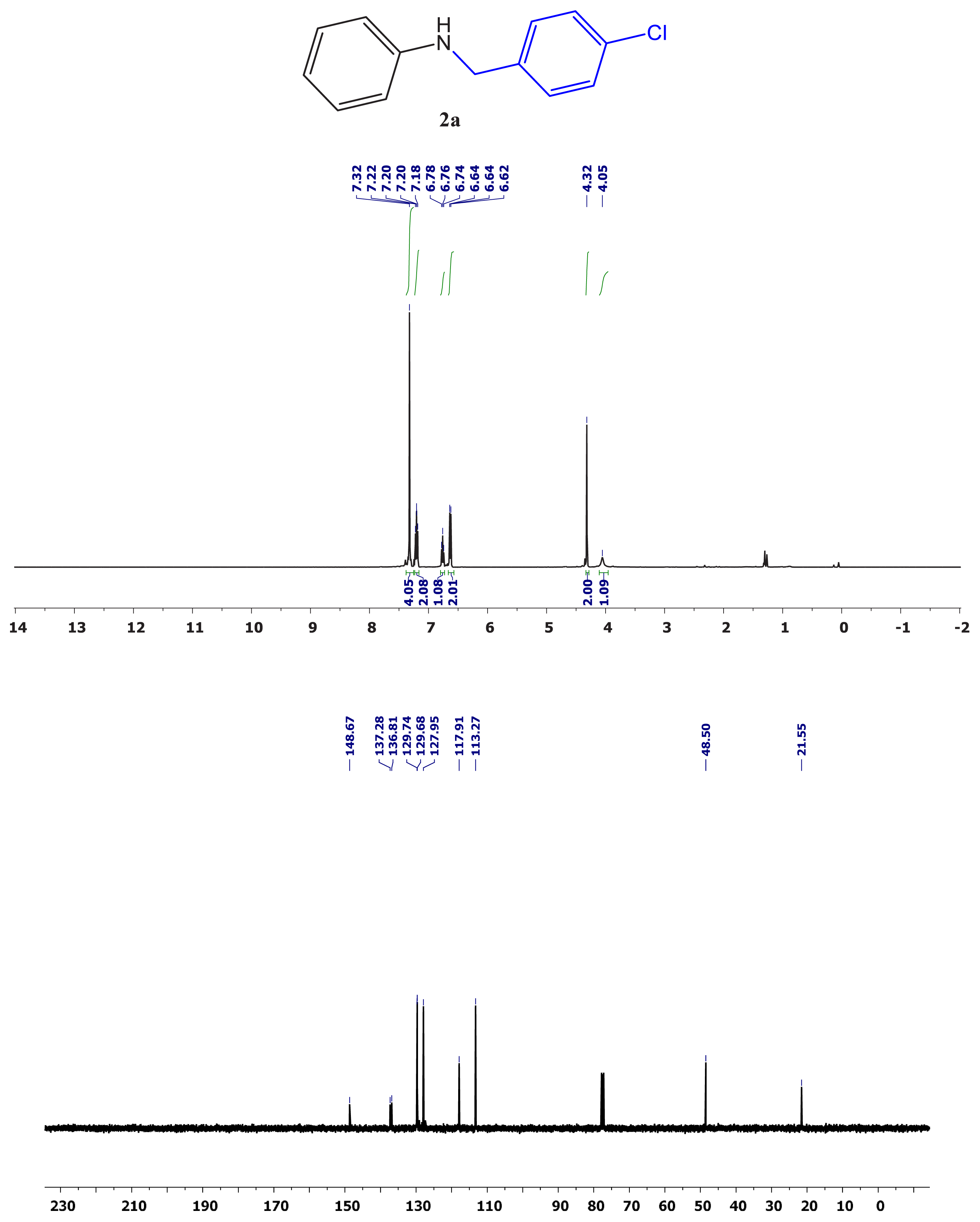

Supplement: Figure S22 — 1H NMR and 13C NMR spectrum of 2a (in CDCl3, 25 °C, TMS, 400 MHz). [file turkjchem-47-5-1209s22.tif]

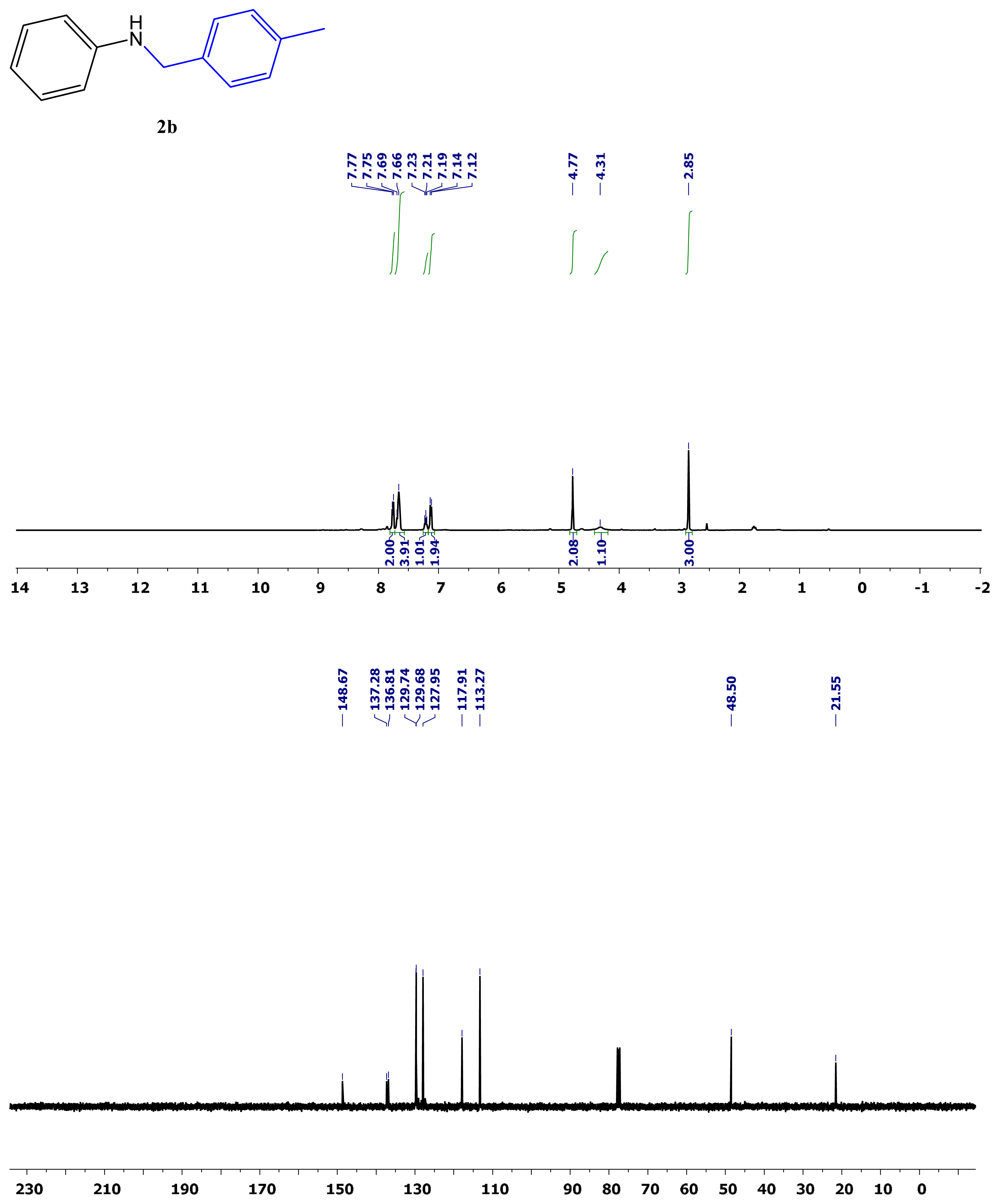

Supplement: Figure S23 — 1H NMR and 13C NMR spectrum of 2b (in CDCl3, 25 °C, TMS, 400 MHz). [file turkjchem-47-5-1209s23.tif]

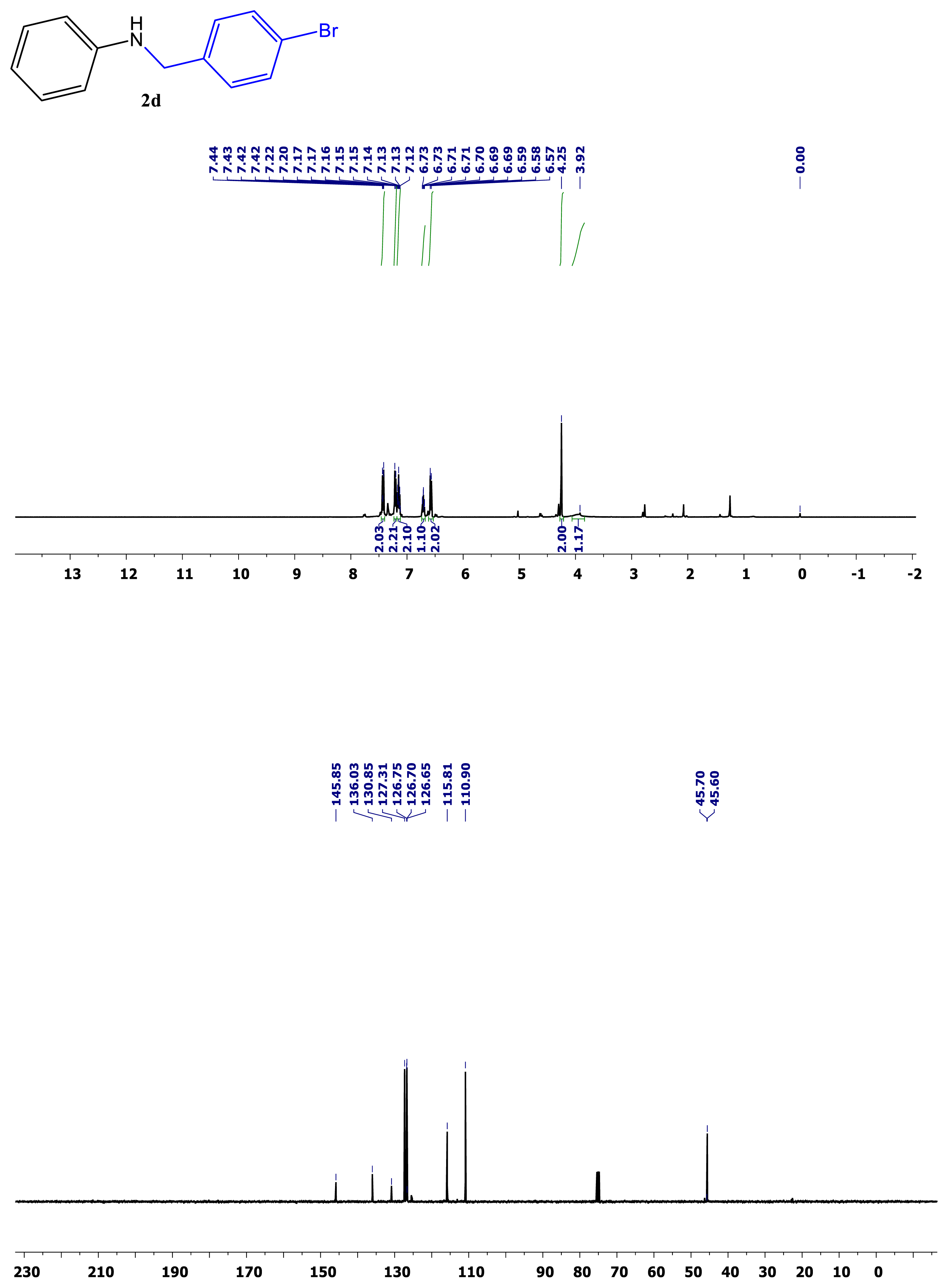

Supplement: Figure S24 — 1H NMR and 13C NMR spectrum of 2c (in CDCl3, 25 °C, TMS, 400 MHz). [file turkjchem-47-5-1209s24.tif]

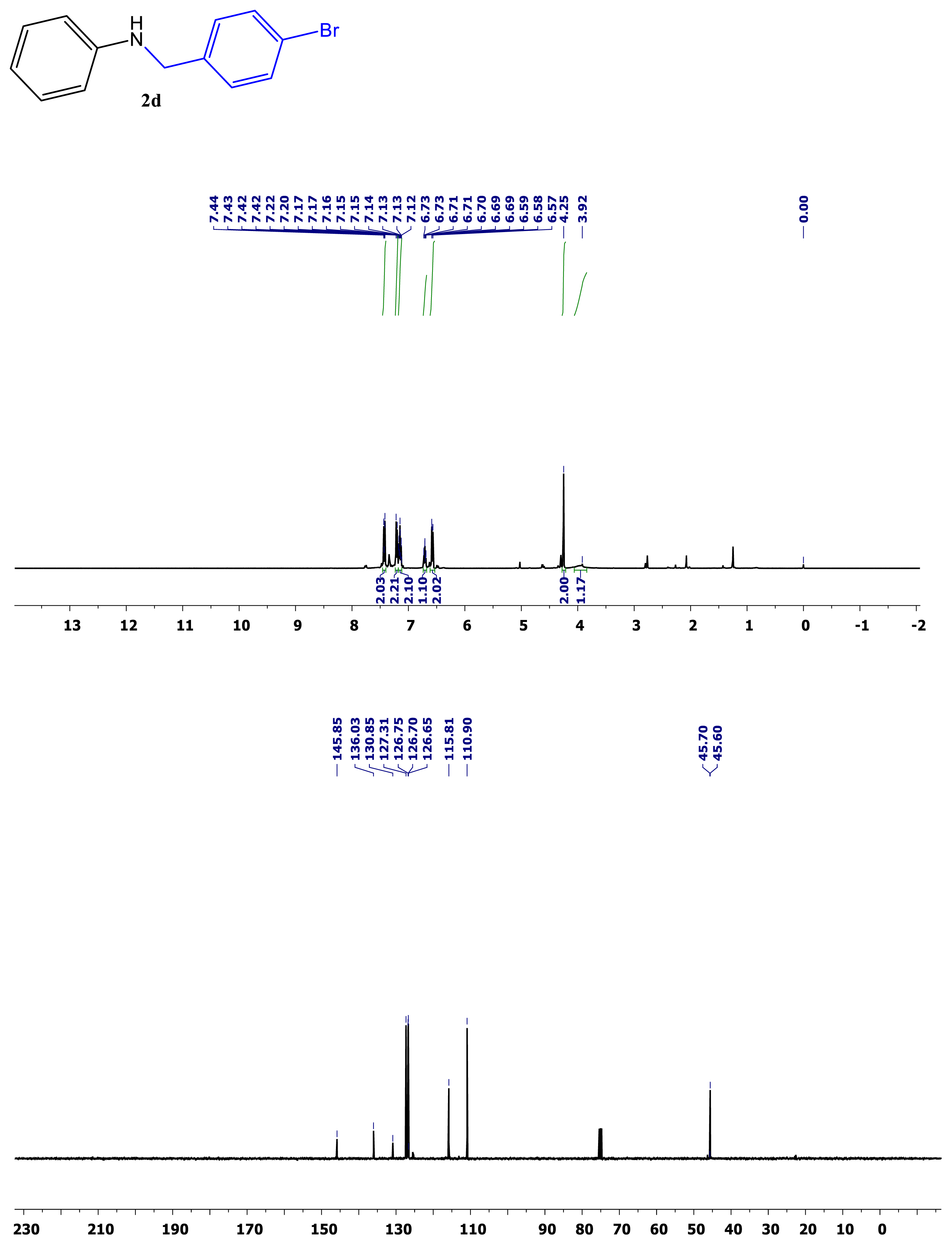

Supplement: Figure S25 — 1H NMR and 13C NMR spectrum of 2d (in CDCl3, 25 °C, TMS, 400 MHz). [file turkjchem-47-5-1209s25.tif]

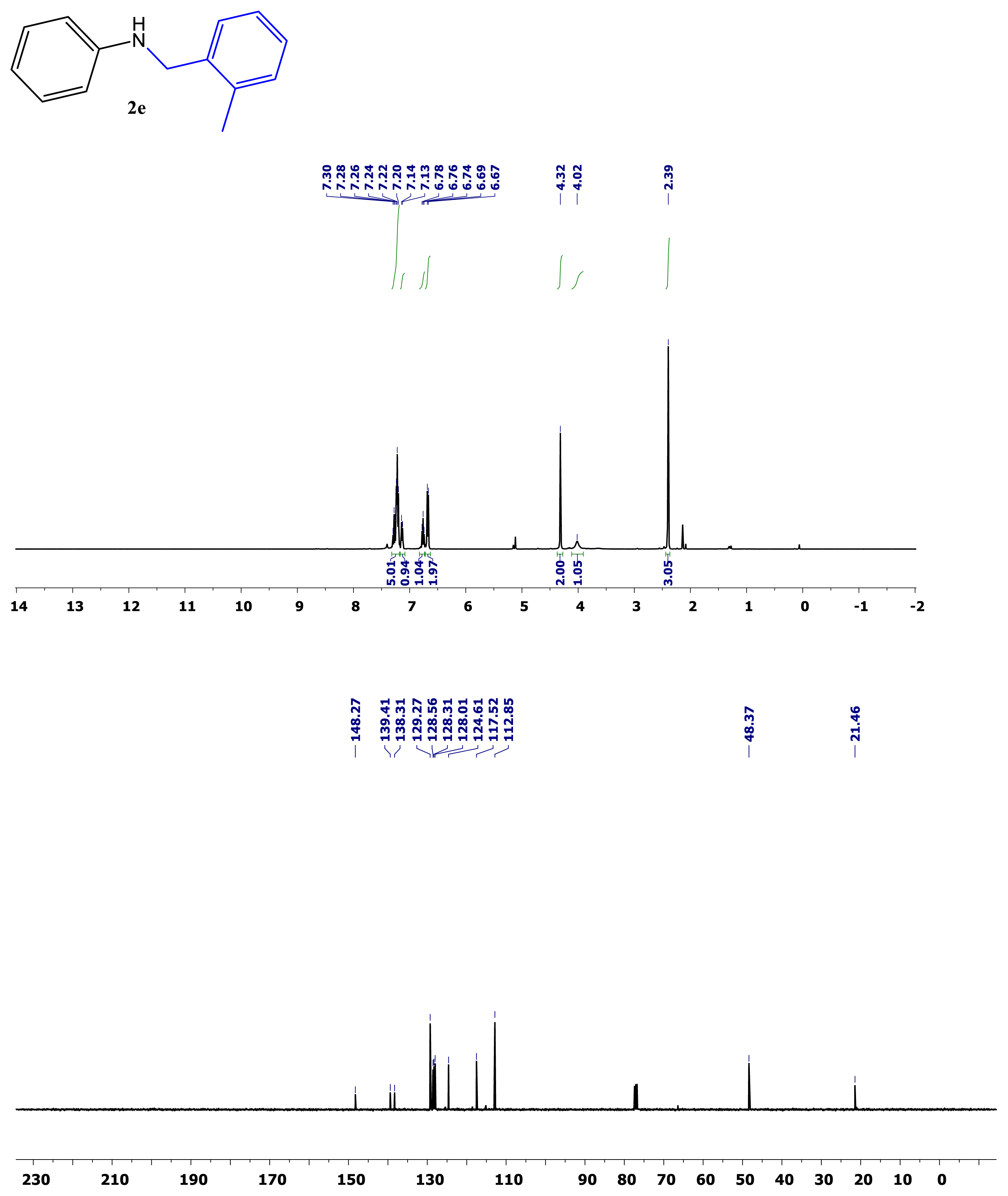

Supplement: Figure S26 — 1H NMR and 13C NMR spectrum of 2e (in CDCl3, 25 °C, TMS, 400 MHz). [file turkjchem-47-5-1209s26.tif]

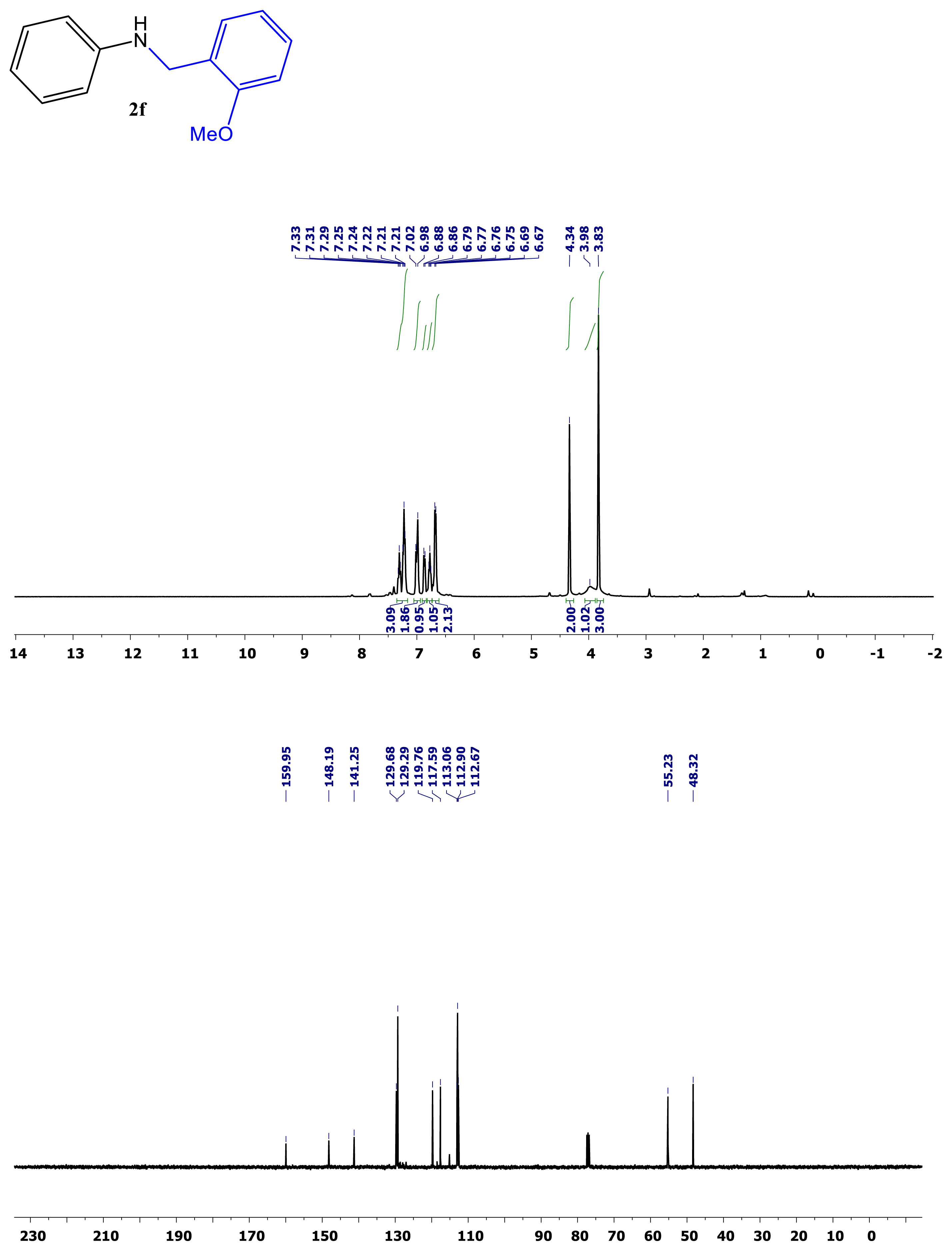

Supplement: Figure S27 — 1H NMR and 13C NMR spectrum of 2f (in CDCl3, 25 °C, TMS, 400 MHz). [file turkjchem-47-5-1209s27.tif]

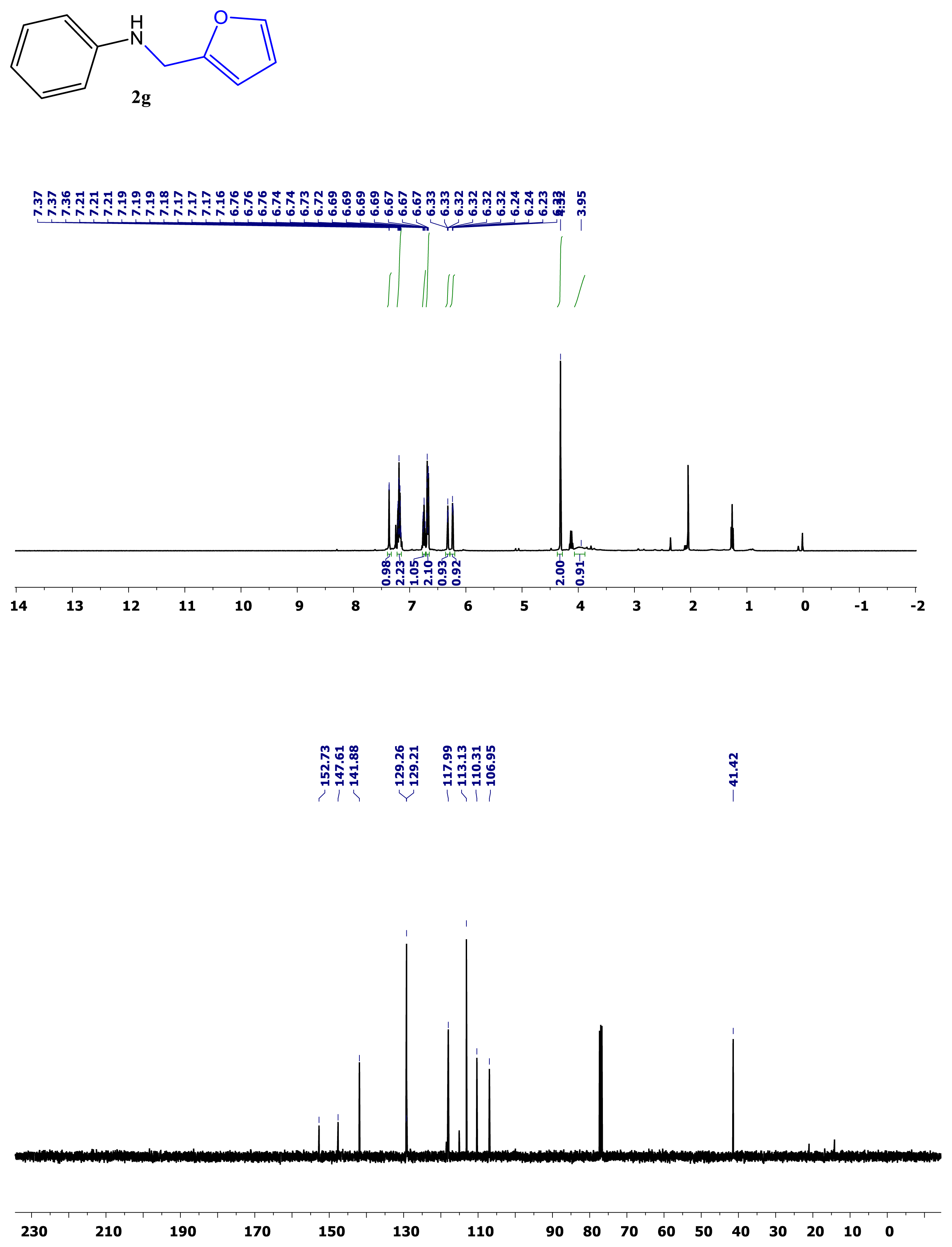

Supplement: Figure S28 — 1H NMR and 13C NMR spectrum of 2g (in CDCl3, 25 °C, TMS, 400 MHz). [file turkjchem-47-5-1209s28.tif]

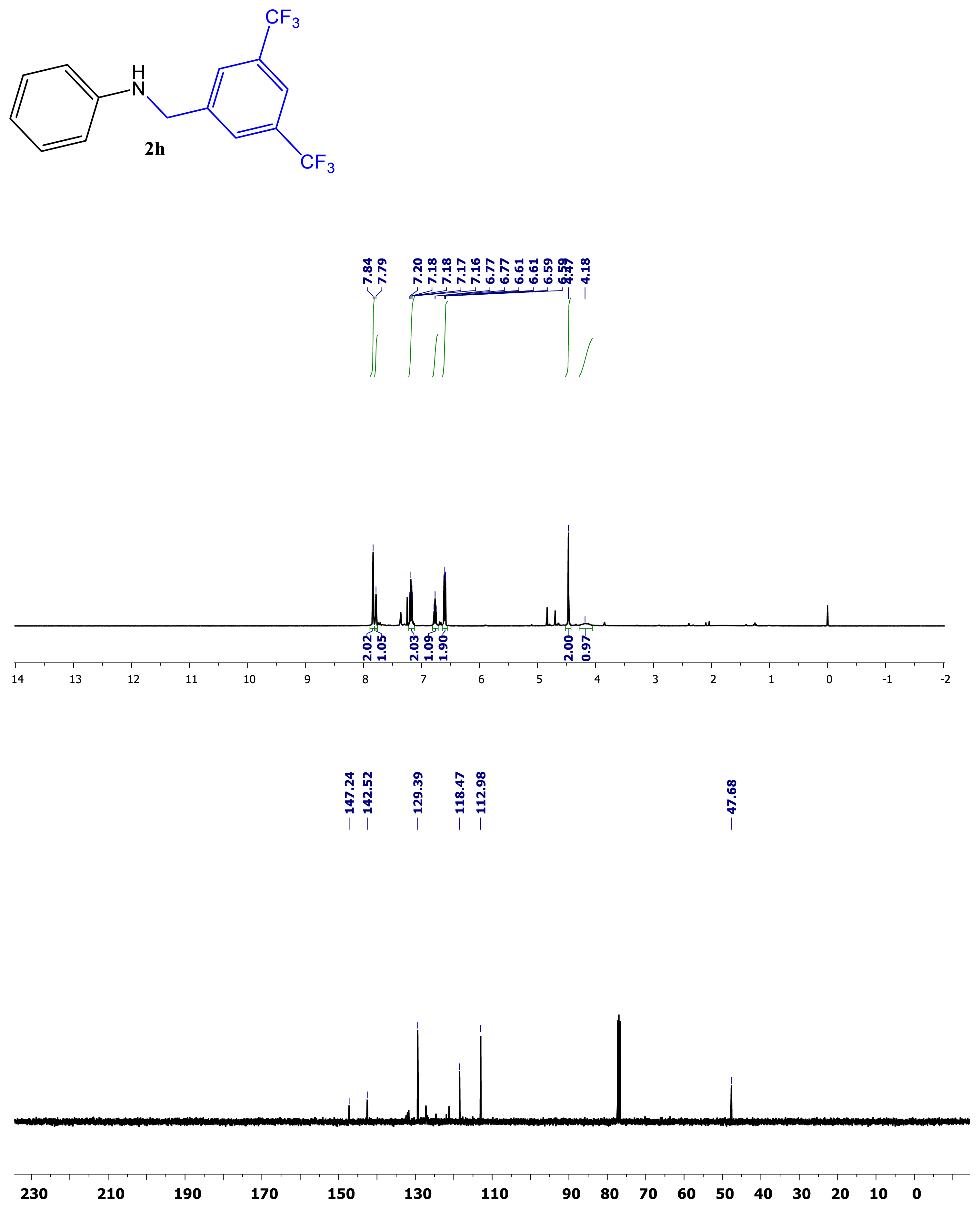

Supplement: Figure S29 — 1H NMR and 13C NMR spectrum of 2h (in CDCl3, 25 °C, TMS, 400 MHz). [file turkjchem-47-5-1209s29.tif]

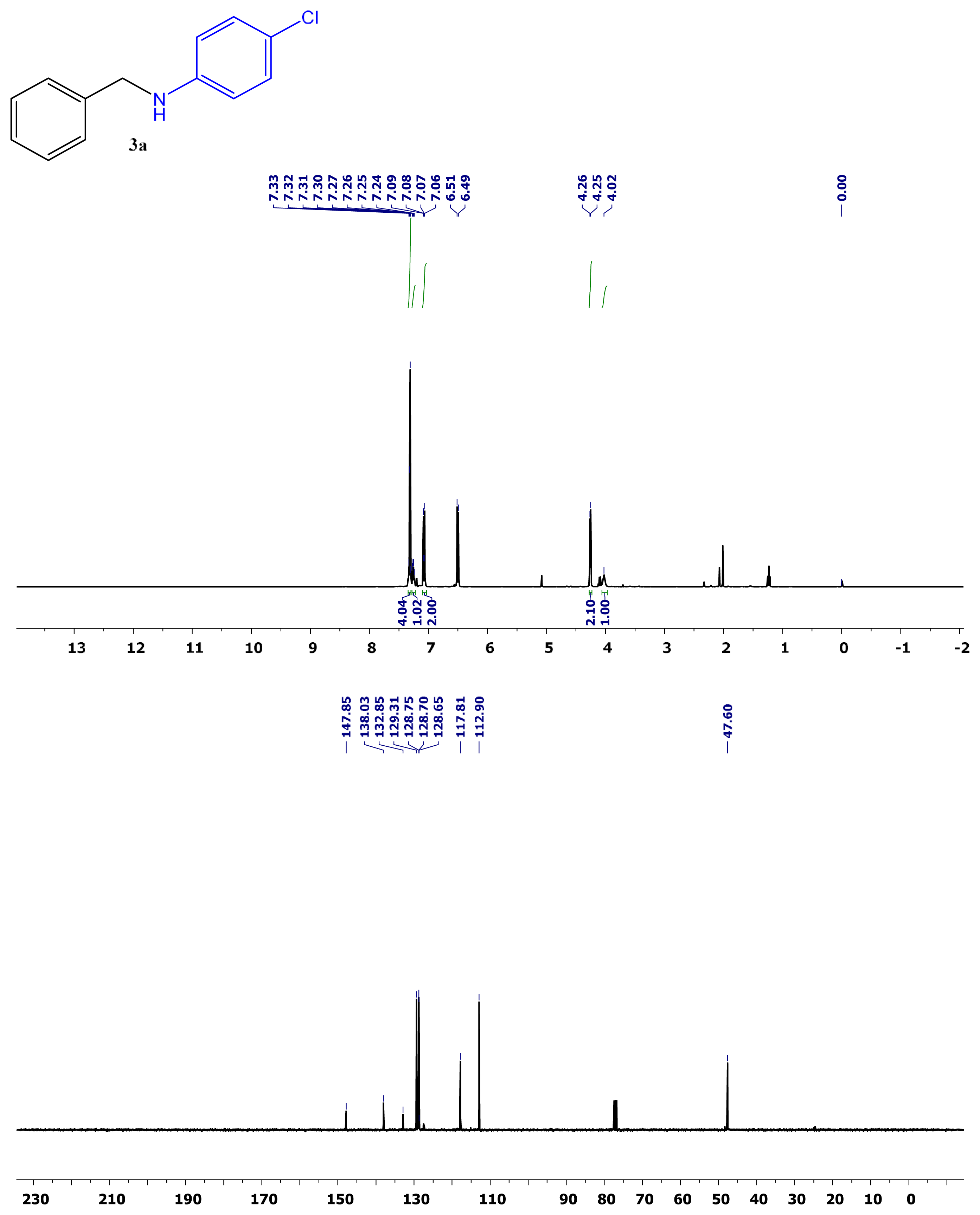

Supplement: Figure S30 — 1H NMR and 13C NMR spectrum of 3a (in CDCl3, 25 °C, TMS, 400 MHz). [file turkjchem-47-5-1209s30.tif]

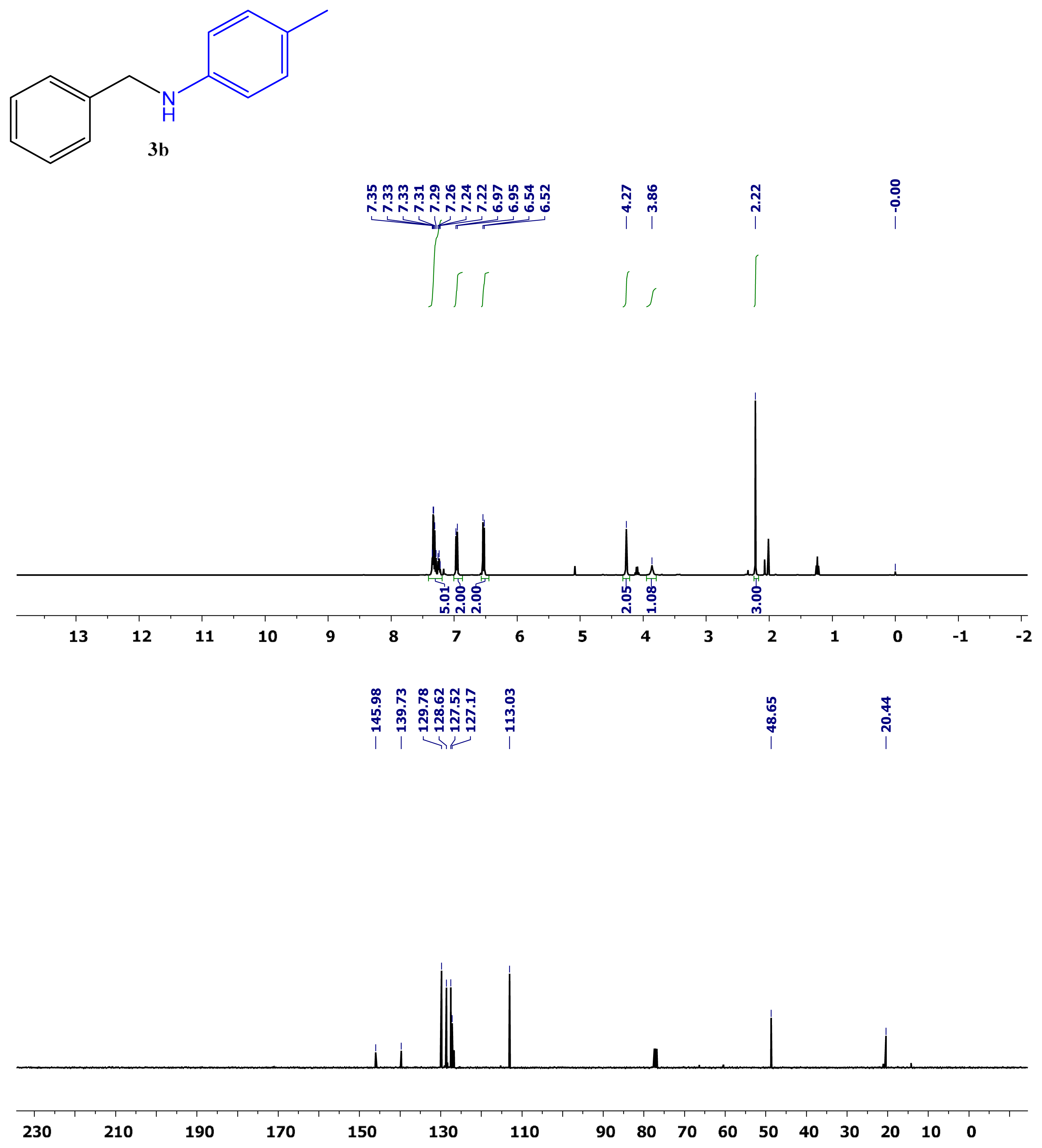

Supplement: Figure S31 — 1H NMR and 13C NMR spectrum of 3b (in CDCl3, 25 °C, TMS, 400 MHz). [file turkjchem-47-5-1209s31.tif]

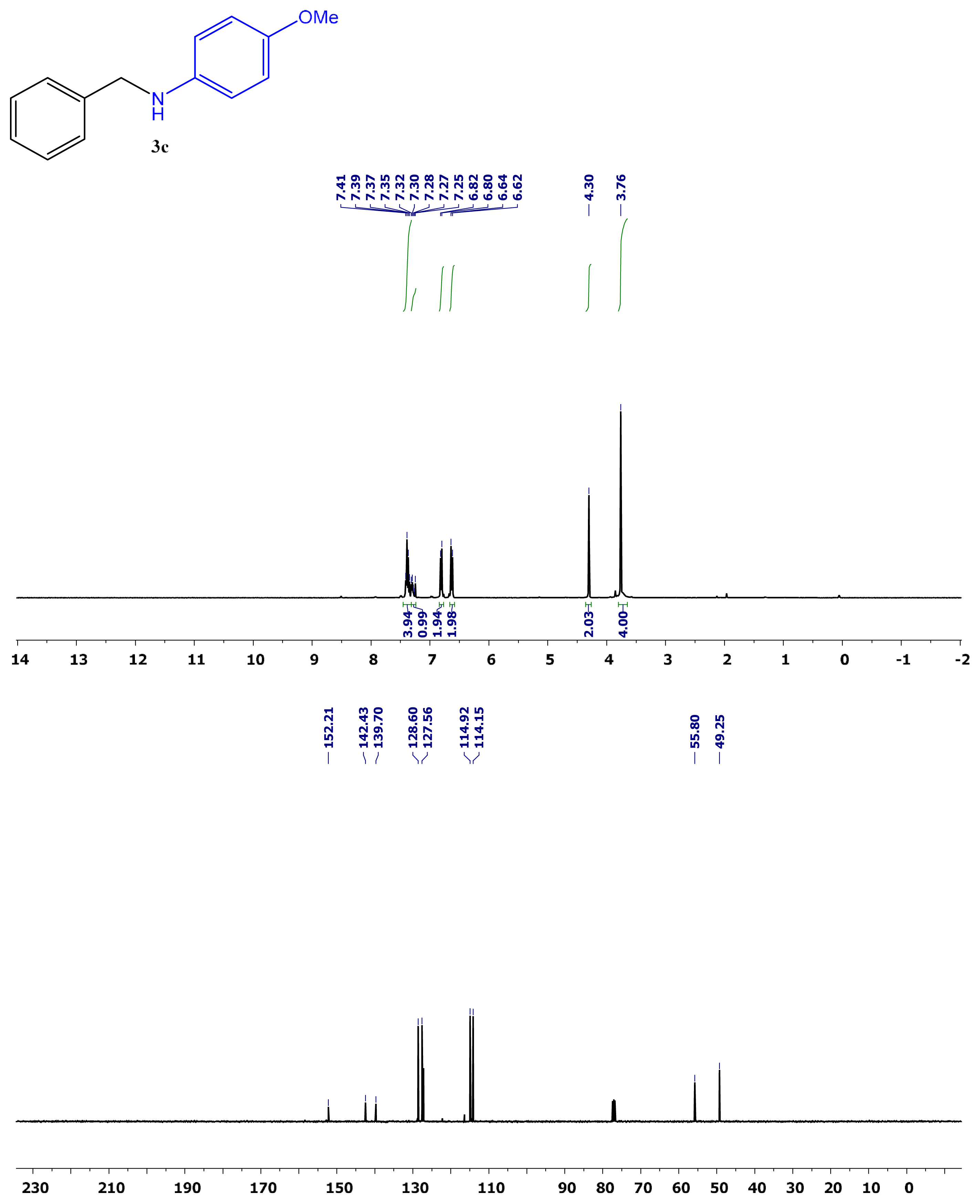

Supplement: Figure S32 — 1H NMR and 13C NMR spectrum of 3c (in CDCl3, 25 °C, TMS, 400 MHz). [file turkjchem-47-5-1209s32.tif]

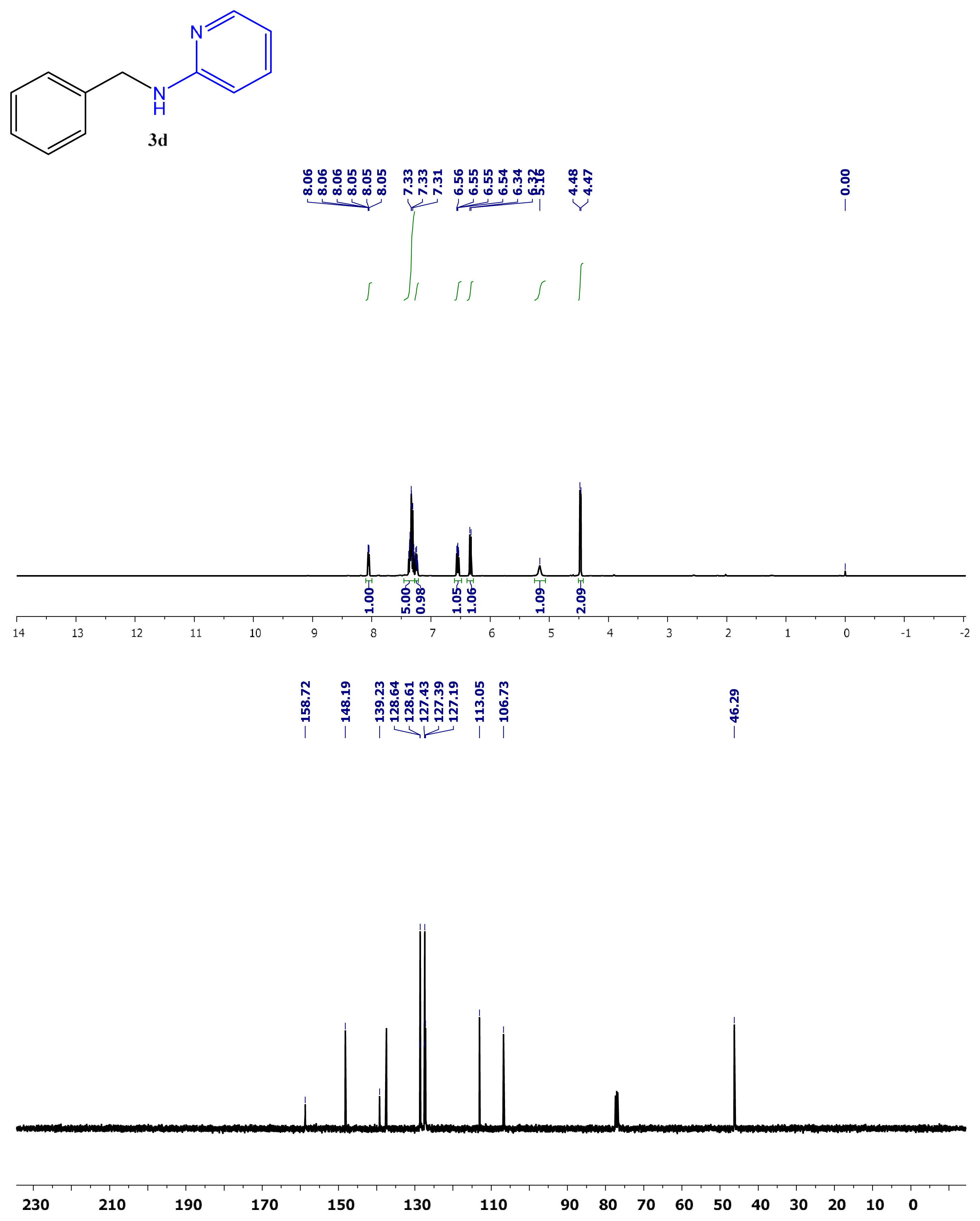

Supplement: Figure S33 — 1H NMR and 13C NMR spectrum of 3d (in CDCl3, 25 °C, TMS, 400 MHz). [file turkjchem-47-5-1209s33.tif]

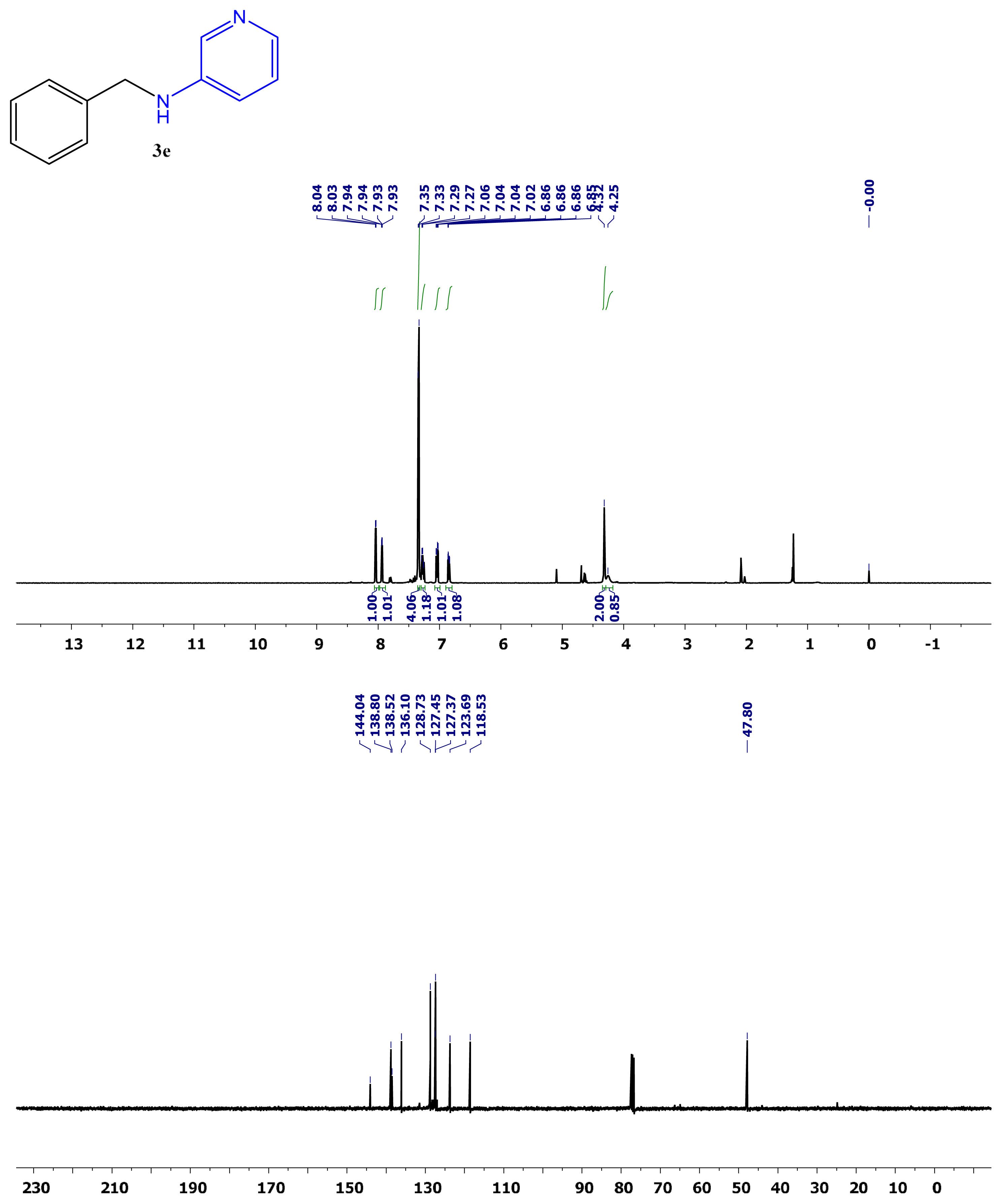

Supplement: Figure S34 — 1H NMR and 13C NMR spectrum of 3e (in CDCl3, 25 °C, TMS, 400 MHz). [file turkjchem-47-5-1209s34.tif]

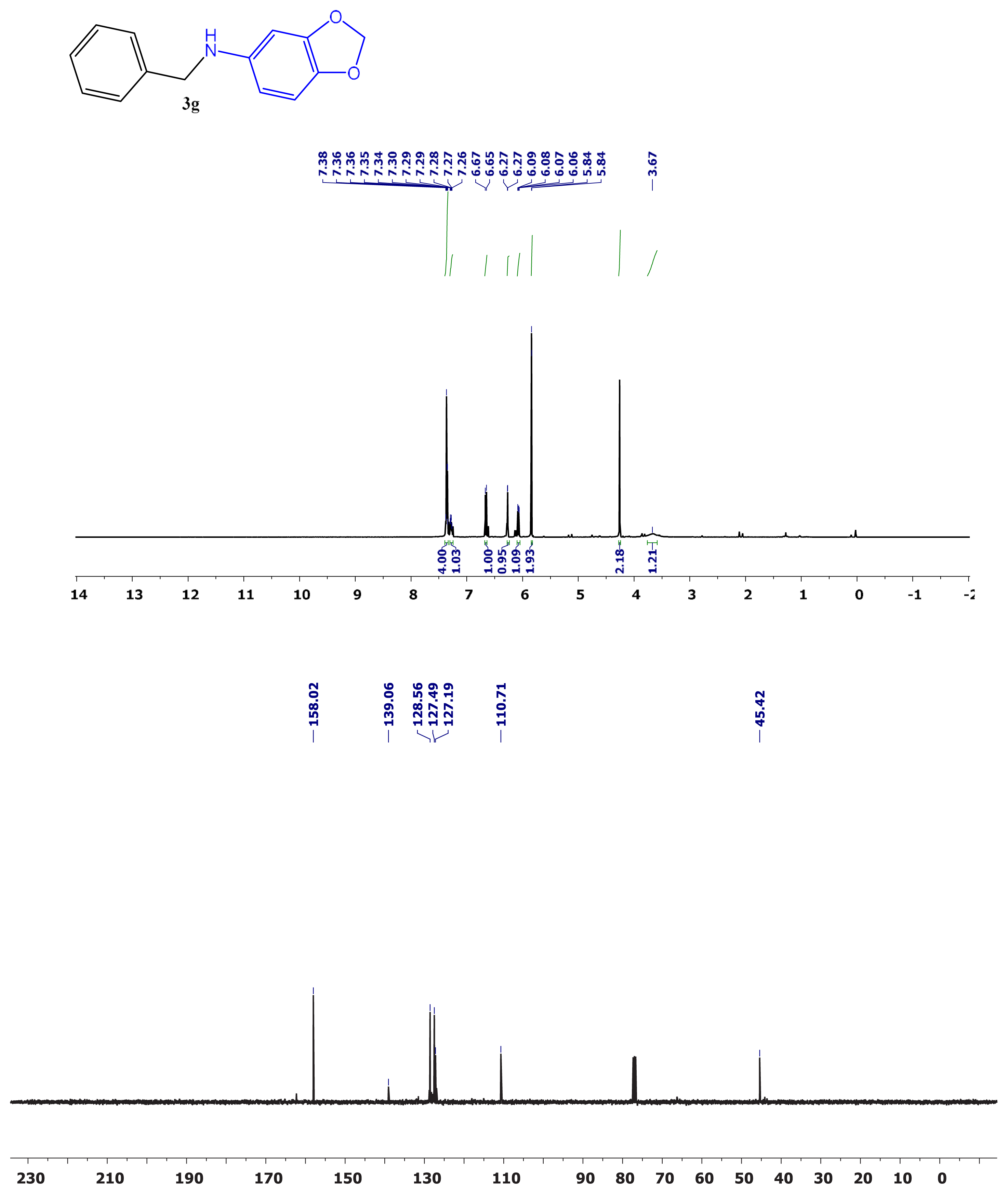

Supplement: Figure S35 — 1H NMR and 13C NMR spectrum of 3f (in CDCl3, 25 °C, TMS, 400 MHz). [file turkjchem-47-5-1209s35.tif]

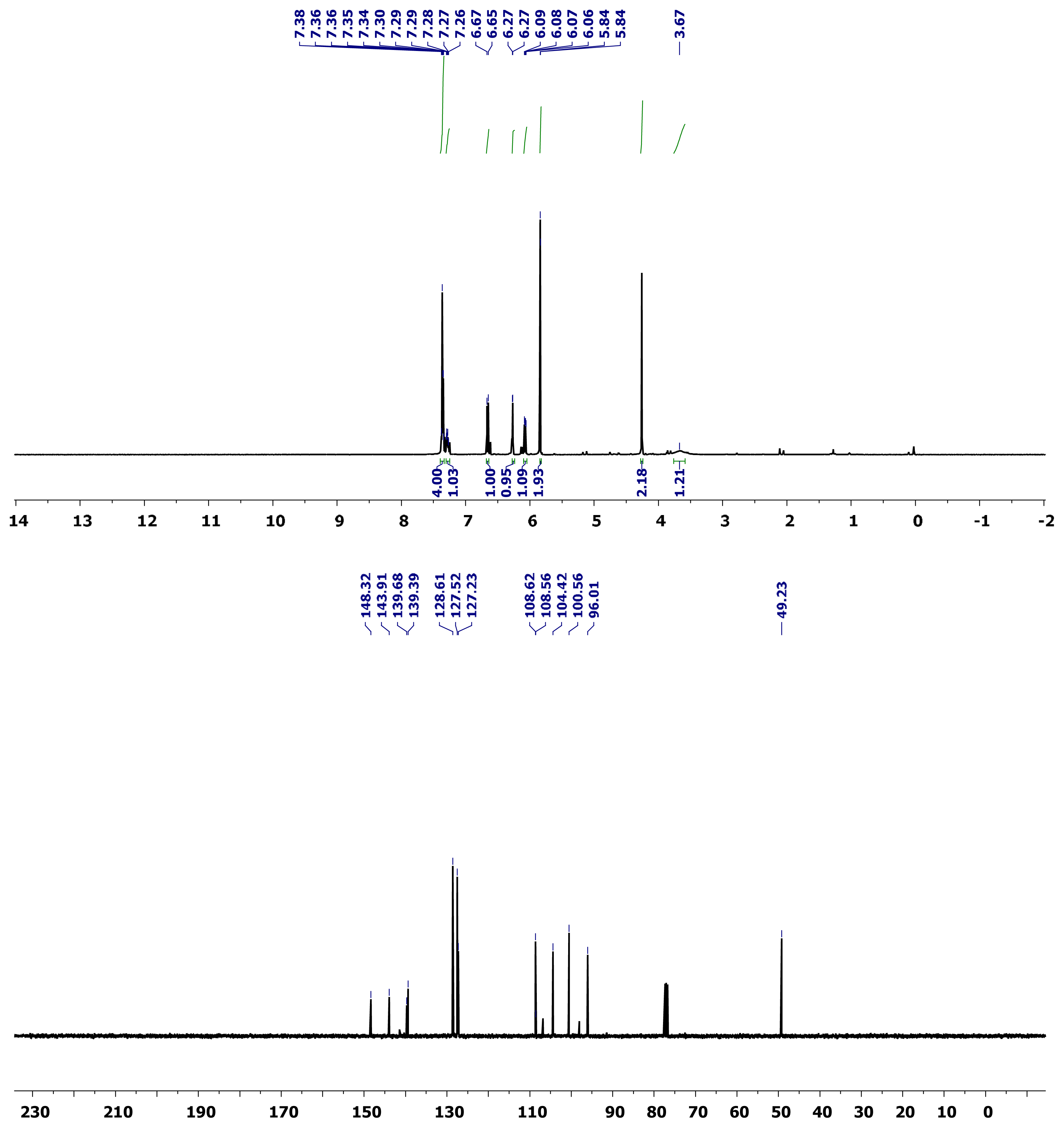

Supplement: Figure S36 — 1H NMR and 13C NMR spectrum of 3g (in CDCl3, 25 °C, TMS, 400 MHz). [file turkjchem-47-5-1209s36.tif]

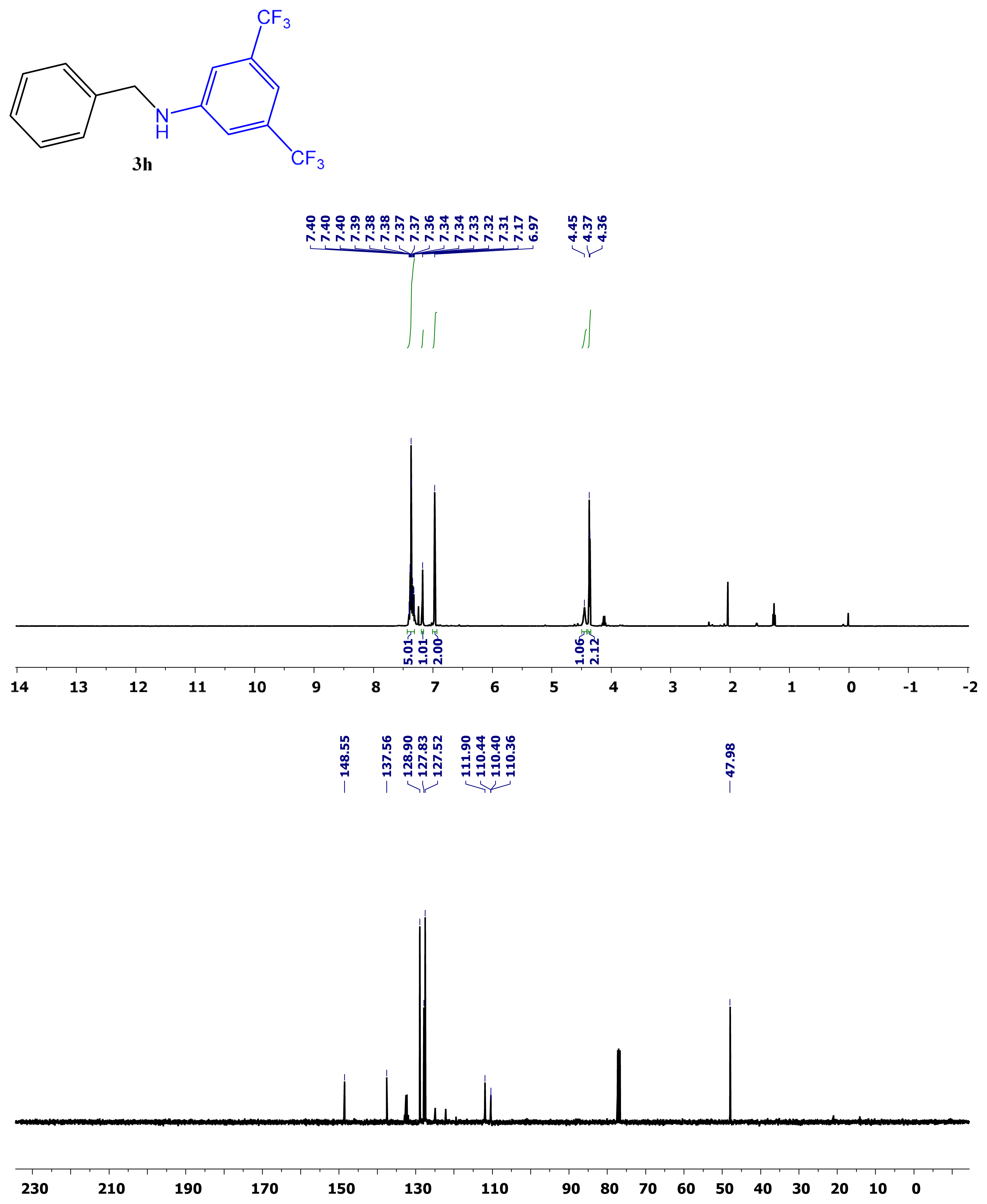

Supplement: Figure S37 — 1H NMR and 13C NMR spectrum of 3h (in CDCl3, 25 °C, TMS, 400 MHz). [file turkjchem-47-5-1209s37.tif]

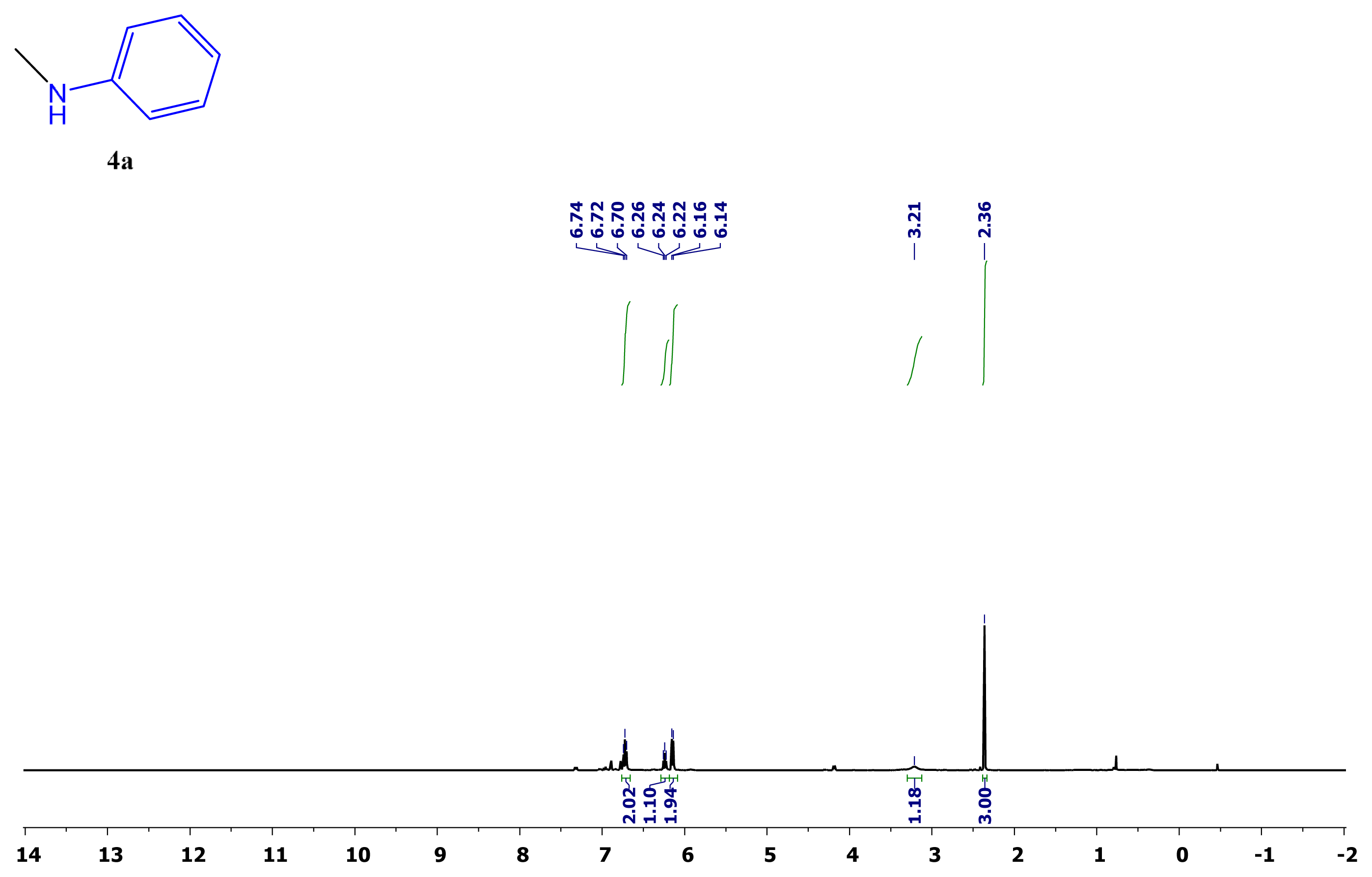

Supplement: Figure S38 — 1H NMR and 13C NMR spectrum of 4a (in CDCl3, 25 °C, TMS, 400 MHz). [file turkjchem-47-5-1209s38.tif]

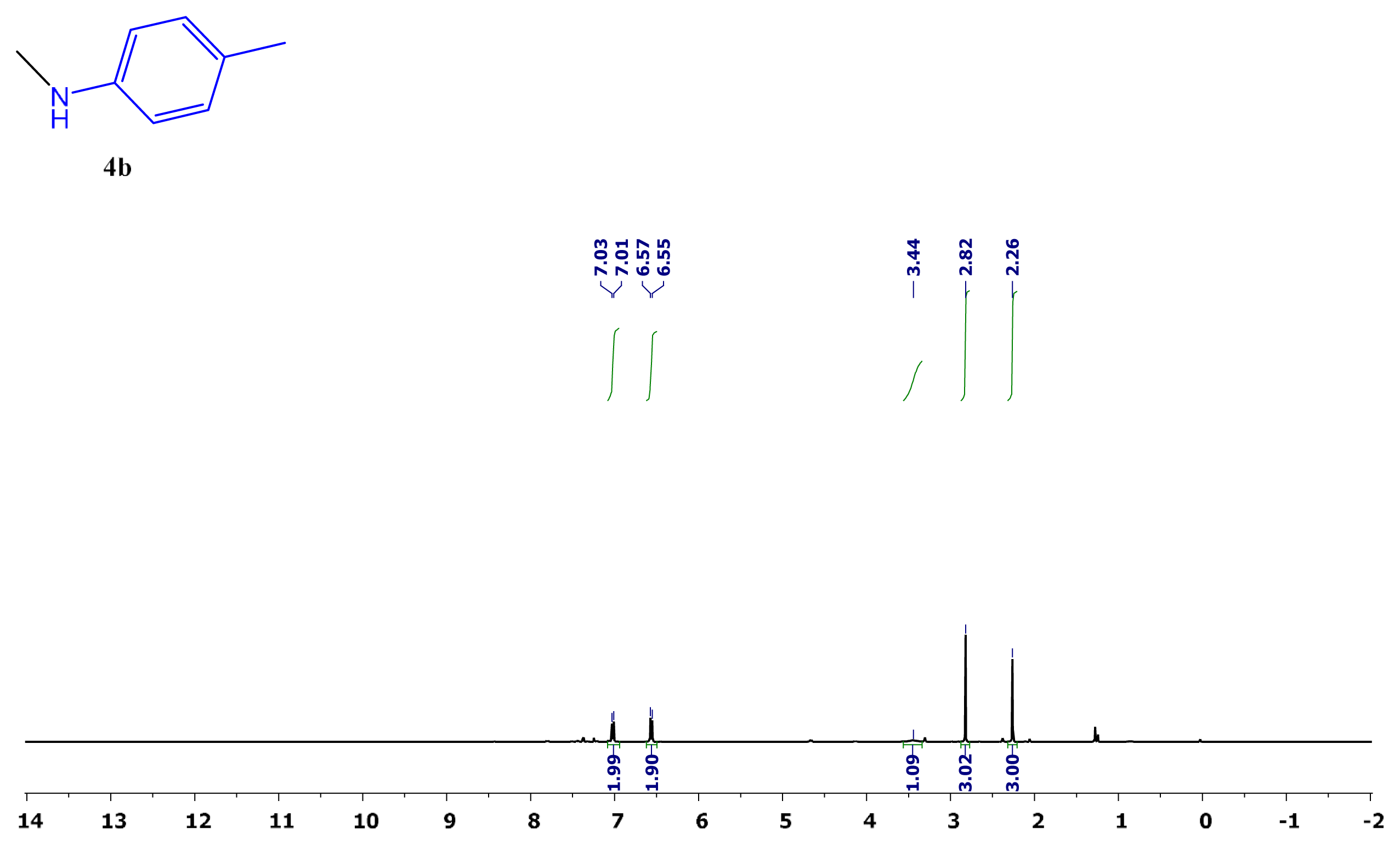

Supplement: Figure S39 — 1H NMR spectrum of 4b (in CDCl3, 25 °C, TMS, 400 MHz). [file turkjchem-47-5-1209s39.tif]

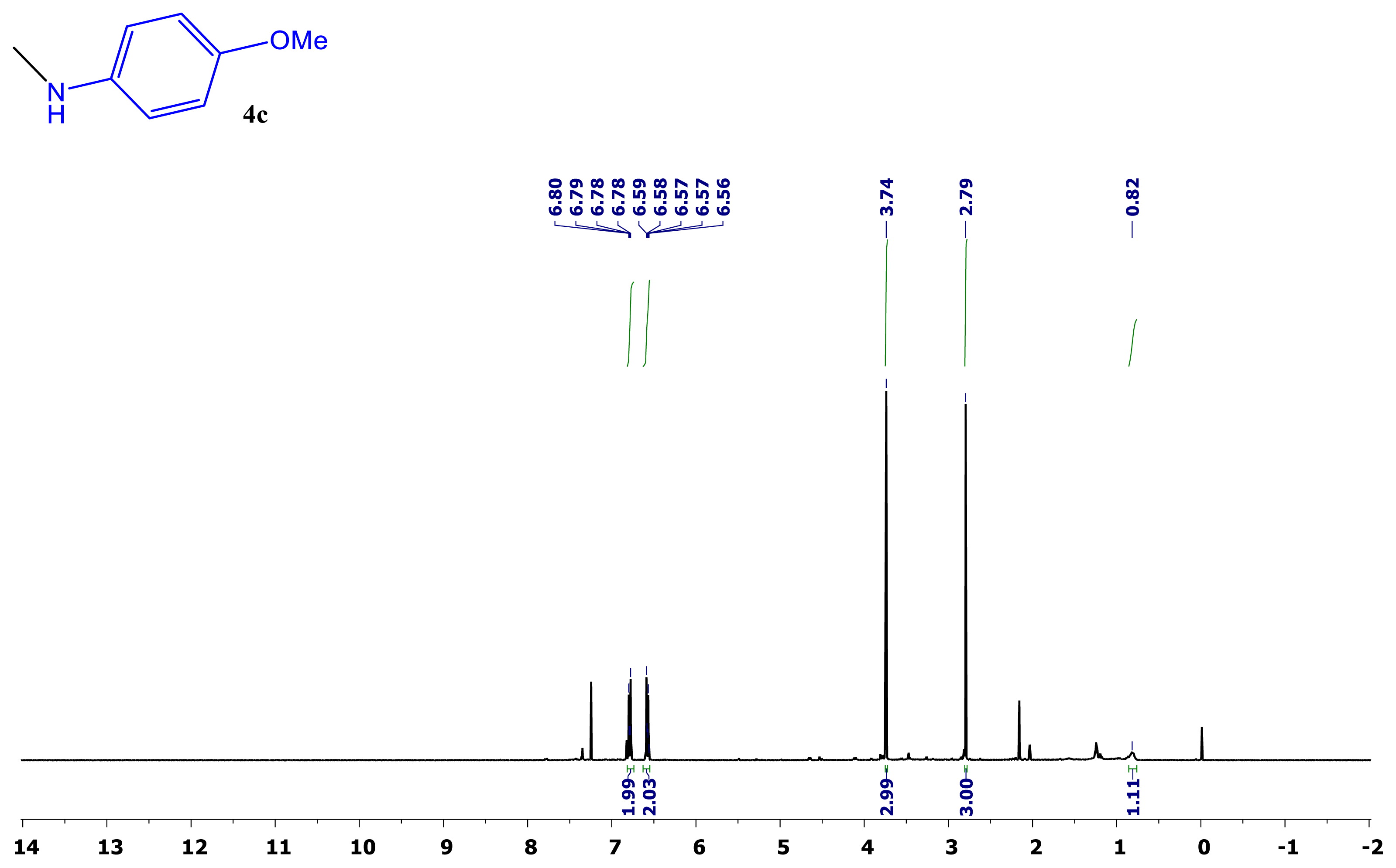

Supplement: Figure S40 — 1H NMR spectrum of 4c (in CDCl3, 25 °C, TMS, 400 MHz). [file turkjchem-47-5-1209s40.tif]

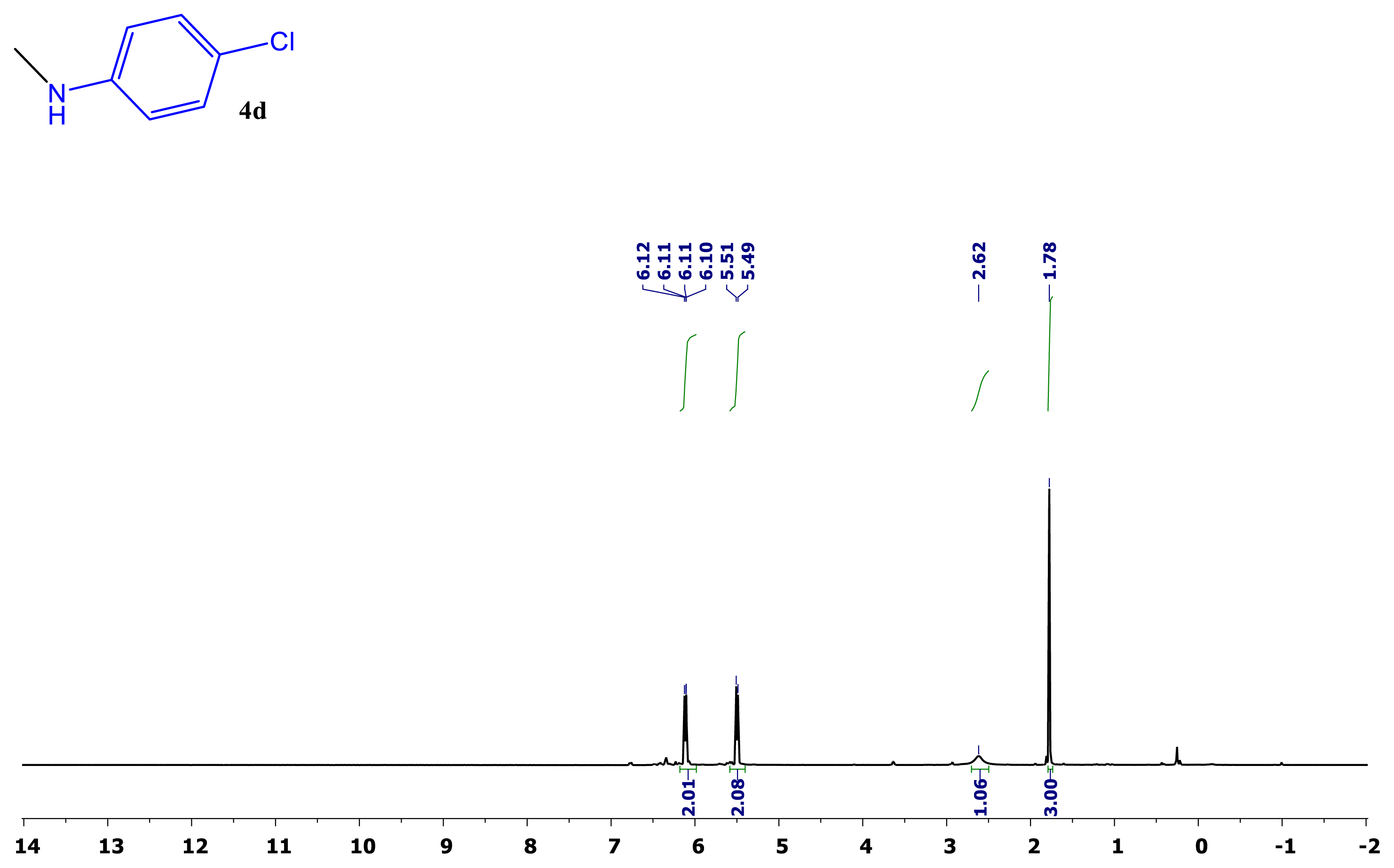

Supplement: Figure S41 — 1H NMR spectrum of 4d (in CDCl3, 25 °C, TMS, 400 MHz). [file turkjchem-47-5-1209s41.tif]

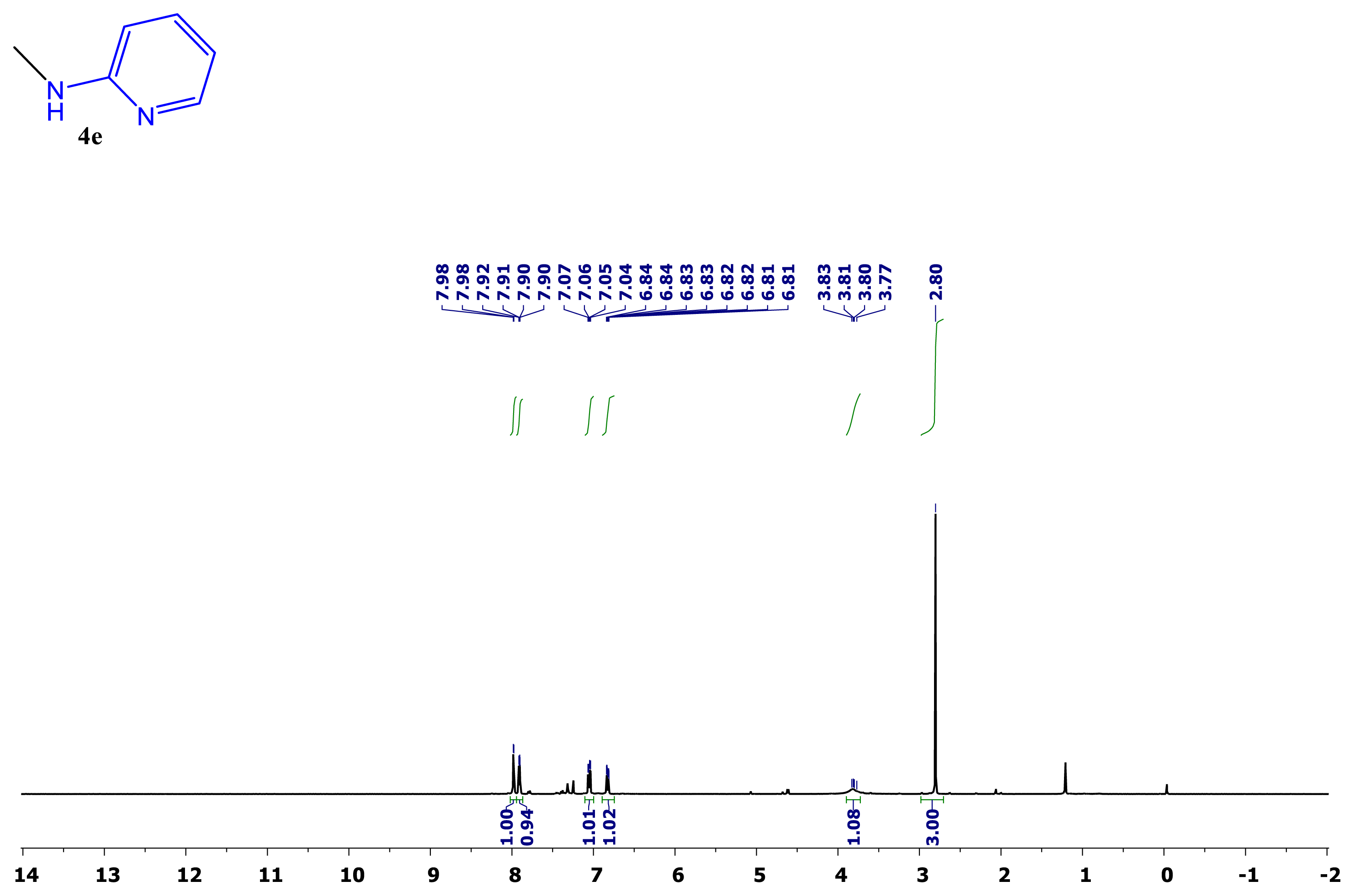

Supplement: Figure S42 — 1H NMR spectrum of 4e (in CDCl3, 25 °C, TMS, 400 MHz). [file turkjchem-47-5-1209s42.tif]

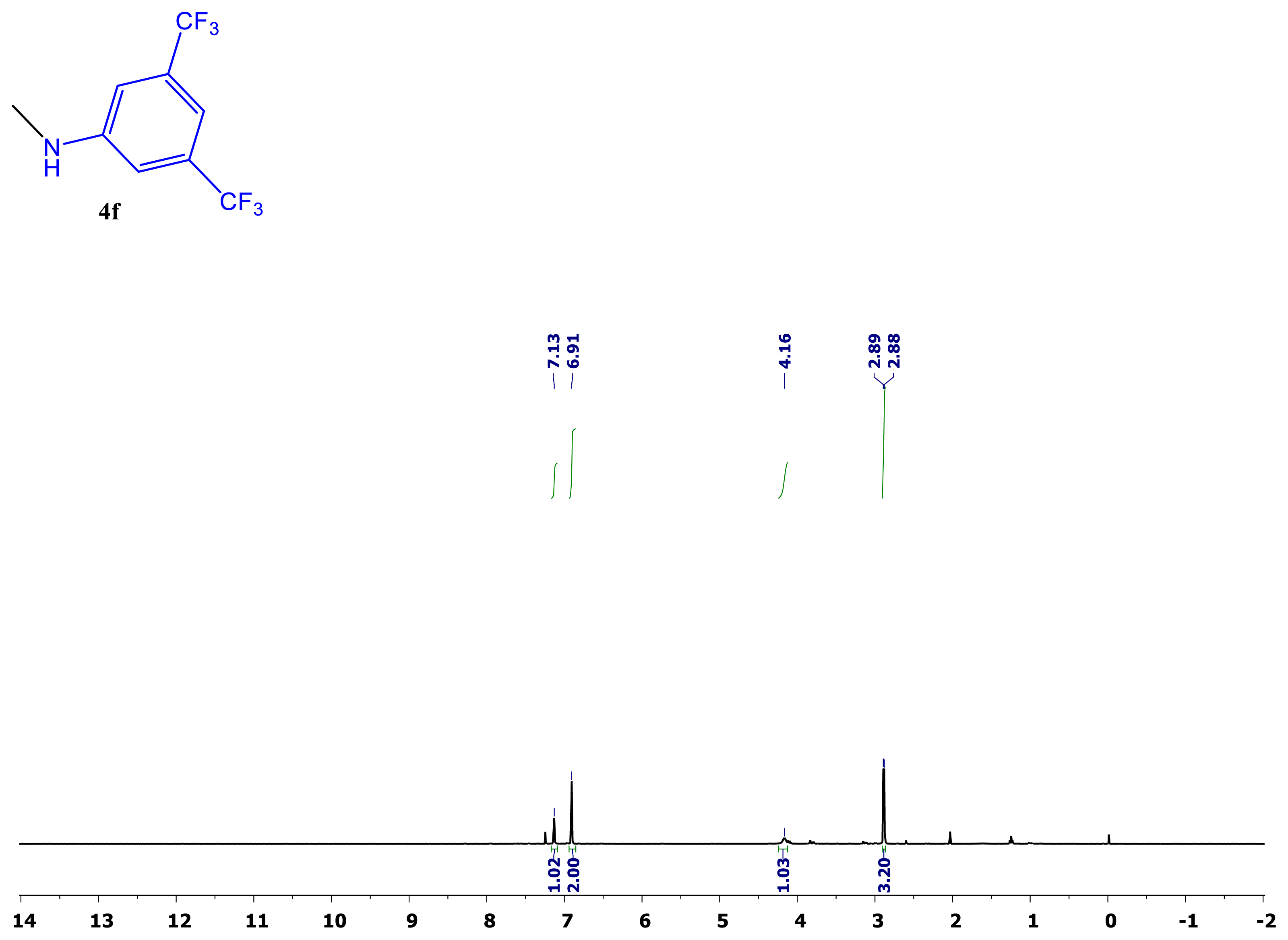

Supplement: Figure S43 — 1H NMR spectrum of 4f (in CDCl3, 25 °C, TMS, 400 MHz). [file turkjchem-47-5-1209s43.tif]
